# Author Correction: Religion and educational mobility in Africa

**DOI:** 10.1038/s41586-023-06886-9

**Published:** 2023-12-12

**Authors:** Alberto Alesina, Sebastian Hohmann, Stelios Michalopoulos, Elias Papaioannou

**Affiliations:** 1 Department of Economics, Harvard University, Cambridge, MA USA; 2 Sihlquai 10, Adliswil, Switzerland; 3 Department of Economics, Brown University, Providence, RI USA; 4 London Business School, London, UK

**Keywords:** Economics, Society

Correction to: *Nature* 10.1038/s41586-023-06051-2 Published online 17 May 2023

In the version of the article initially published, there were errors in the numbers on the x axis and the corresponding values in the bars in Fig. 2. The original and corrected versions of Fig. 2 appear as Fig. 1 below. The figure has now been corrected in the HTML and PDF versions of the article.

**Fig. 1:**
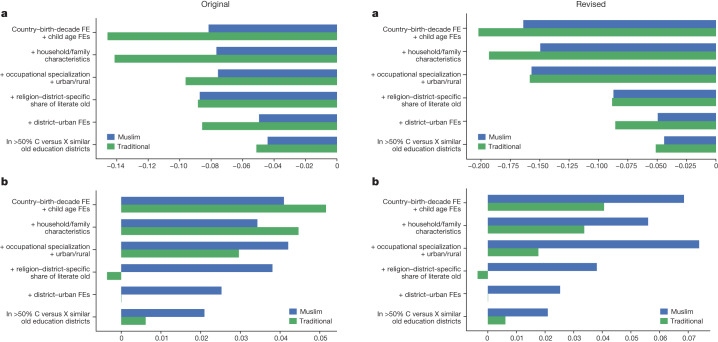
Original and corrected Fig. 2 .

