# Supplementary material for: Religion and educational mobility in Africa
Source: Nature. 2023 May 17;618(7963):134–43. doi: 10.1038/s41586-023-06051-2 (PMC10232358; doi:10.1038/s41586-023-06051-2)
Supplement: Supplementary file 1 — Supplementary Information Supplementary Sections A–F. [file 41586_2023_6051_MOESM1_ESM.pdf]

nature portfolio

https://doi.org/10.1038/s41586-023-06051-2

Supplementary information

Religion and educational mobility in Africa

In the format provided by the authors and unedited

Supplementary Information

Contents of Supplementary Information

A Descriptive Patterns and Introductory Material **1**
A.1 Returns to Schooling in Africa . . . 1
A.2 Country Patterns . . . 2
A.3 Pan-African Patterns . . . 5
A.4 Regional Patterns . . . 5

B Data and Sample **8**
B.1 Sample Construction . . . 8
B.2 Cohabitation Rates . . . 16
B.3 Inter-generational Transmission of Religious Affiliation . . . 18
B.4 Religious Affiliation and Ethnicity . . . 21
B.4.1 Patterns . . . 21
B.4.2 Examples . . . 22
B.5 Comparing African Countries in IPUMS Sample and Out of Sample . . . 22

C Religious Intergenerational Mobility across Countries and Regions **27**
C.1 Additional Country-Level Religious Educational IM Measures . . . 27
C.2 Regional IM across Religious Affiliation . . . 29
C.3 Summary Statistics. Regional Educational IM Series . . . 32

D Drivers of Religious Intergenerational Mobility Gaps **35**
D.1 Gender Differences . . . 35
D.2 Sensitivity Checks . . . 37
D.2.1 Biological Children . . . 37
D.2.2 Ethnicity . . . 38
D.3 Family Organization, and Economic Structure Inter-Religious Differences . . . 40
D.3.1 Household and Family Characteristics . . . 40
D.3.2 Occupation and Employment Features . . . 42
D.4 Matching Districts. Old Generation's Education by Religion . . . 45

E Childhood Regional Exposure Effects **48**
E.1 Country Sample . . . 48
E.2 Parametric Estimates and Sensitivity Analysis . . . 48
E.3 Gender Differences . . . 52

F Variable Sources and Definitions **54**
F.1 At-Independence Features . . . 54
F.2 Geographical and Location Features . . . 54
F.3 Historical Features . . . 55
F.4 Religious Composition . . . 55

A Descriptive Patterns and Introductory Material

This Supplementary Information (SI) Section provides descriptive evidence on the evolution of education across countries and regions, complementing the discussion in the paper's introduction. We first present some studies quantifying the returns to schooling in low-income countries and Africa. Second, we present patterns illustrating the evolution of education across countries since independence-decolonization for the main religious groups. Third, we give Africa-wide patterns across all sample countries and regions on the evolution of educational attainment for Christians, Muslims, and Africans adhering to Traditional religions.

A.1 Returns to Schooling in Africa

The literature on the returns to education is voluminous. Below, we summarize some studies zooming into Africa.

Mincerian Returns Both correlational and causal estimates reveal considerable individual, often referred to as Mincerian, returns on income/wages of formal education (Card, 1999). Montenegro and Patrinos (2014) global harmonization of Mincerian returns suggests considerable wage/income benefits of schooling for boys and girls across Africa. Africa has the highest cross-continental average (12.4% with a world mean of 8.7%). The five countries among the 139 studied with the highest returns to schooling are all African [Rwanda, Namibia, Burundi, Ethiopia, and South Africa]. Moreover, the comparative analysis that spans 819 harmonized household surveys suggests higher private returns to completed primary enrollment compared to secondary and tertiary education across the world and in Africa. These patterns are broadly in line with the subsequent decennial update of the World Bank's team on education, spanning 1,120 Mincerian return estimates from 139 countries (Psacharopoulos and Patrinos, 2018). Average returns worldwide are about 8.8%, whereas, in Africa, the mean is 10.5%. [Private returns to primary education are 25.4% quite similar across rich, middle, and low-income countries.] In the same vein, Young (2012) also estimates private education returns of 11.6% using DHS from 14 Sub-Saharan African countries. The somewhat higher returns to formal education in low(er) income countries and Africa also feature in other comparative works. For example, the estimates in Caselli et al. (2014) suggest private returns to schooling of about 8.4% in Africa (and the Middle East) quite stable across time, compared to about 7.5% in advanced economies.

Economic Returns Studies looking at the role of formal education in other than wages and labor income proxies also uncover significantly positive correlations. Using the DHS surveys, Young (2012) documents strong positive associations between educational attainment and ownership of durables (radio, bicycle, car, telephones), housing conditions (sources of drinking water, flush toilet, access to electricity), children's nutrition and health (e.g., height, weight, survival of children at birth, absence of disease). The significance of the schooling with well-being correlation is present, even when he exploits country x survey x urban/rural variation and conditions on numerous household and individuals demographic features

similar to Psacharopoulos (1994) much-cited mean of 12.4%.

Health Numerous observational and experimental studies uncover significant educational benefits for health outcomes in Africa. For example, (primary) education correlates with lower infant mortality (Kiross et al., 2019), diminished HIV infection (Melesse et al., 2020), and lower fertility (World Bank, 2018, Vogl, 2022, Duflo et al., 2015).

A.2 Country Patterns

SI Table A.1 reports completed primary schooling for Christians, Muslims, and Traditionalists, older than 14 years born in the 1950s and in the 1990s across all sample countries. For the cohort born just before African independence, we see that in 15 countries Christians have higher primary completion rates than Muslims and Traditionalists; the largest differences are in West Africa, Ghana, Cameroon, Nigeria, Togo, and Sierra Leone. Primary education completion rates are higher for Muslims in Botswana, South Africa, Mauritius, Rwanda, Uganda, and Zambia. Inter-religious differences largely persist. In some countries, like Senegal and Ethiopia, the Christian-Muslim gap in completed primary education increases. Muslims' primary completion rates for the 1990-born exceed Christians' in four countries, as Christians overtake in Botswana and South Africa.

SI Table A.2 gives mean years of schooling for Africans, aged 25 years and older, born in the 1940s and the 1980s for the three main religions. Upon independence, Christians had significantly higher schooling except for Botswana, South Africa, Rwanda, and Zambia, where Muslims are less than 2% of the population (see Extended Data Table 1). The educational differences have endured during the post-colonial era. In most countries, the Christian-Muslim gap in schooling years has increased (e.g., Nigeria, Cameroon, Mali, and Burkina Faso) or remained constant (e.g., Mozambique, Guinea, and Togo).

	1950s				1990s			
	(1)	(2)	(3)	(4)	(5)	(6)	(7)	(8)
country	Christian	Muslim	Traditional	$\Delta(c-m)$	Christian	Muslim	Traditional	$\Delta(c-m)$
Ghana	0.62	0.23	0.12		0.77	0.57	0.3	
Cameroon	0.68	0.3	0.21		0.76	0.42	0.39	
Nigeria	0.55	0.23	0.29		0.88	0.57	0.35	
Togo	0.5	0.19	0.11		0.73	0.57	0.42	
Sierra Leone	0.43	0.15	0.04		0.49	0.29	0.25	
Senegal	0.47	0.22			0.8	0.45		
Benin	0.35	0.12	0.04		0.65	0.4	0.45	
Guinea	0.36	0.14	0.08		0.58	0.4	0.41	
Egypt	0.47	0.31			0.88	0.87		
Malawi	0.25	0.11	0.16		0.31	0.2	0.18	
Burkina Faso	0.19	0.05	0.01		0.36	0.19	0.08	
Liberia	0.36	0.23	0.14		0.32	0.3	0.14	
Mali	0.21	0.11	0.02		0.43	0.3	0.18	
Ethiopia	0.09	0.03	0.02		0.29	0.16	0.07	
Mozambique	0.1	0.06	0.12		0.2	0.12	0.23	
Zambia	0.55	0.57	0.5		0.72	0.76	0.7	
Uganda	0.37	0.41	0.05		0.7	0.75	0.32	
Mauritius	0.79	0.85			0.97	0.99		
Rwanda	0.2	0.32	0.03		0.39	0.55	0.38	
South Africa	0.65	0.88	0.33		0.97	0.96	0.94	
Botswana	0.51	0.78	0.24		0.96	0.92	0.82	

Table A.1: Country-Religious Group Share of Completed Primary Education

The table reports the share of completed primary education for individuals older than 14 years old for the 1950s and 1990s cohorts (because of census timing, the Sierra Leone values correspond to the 1980s cohort). Columns (1)-(3) show the shares for the 1950s-born, Columns (5)-(7) for the 1990s-born. Columns (4) and (8) show differences between Christians and Muslims. Red bars indicate a negative difference, green bars a positive difference.

	1940s				1980s			
	(1)	(2)	(3)	(4)	(5)	(6)	(7)	(8)
country	Christian	Muslim	Traditional	$\Delta(c-m)$	Christian	Muslim	Traditional	$\Delta(c-m)$
Ghana	6.02	1.85	0.98		8.26	4.55	1.94	
Sierra Leone	3.88	0.84	0.0		4.53	2.2	1.31	
Senegal	3.76	1.2			7.73	3.29		
Togo	3.51	1.07	0.52		7.12	4.35	2.78	
Cameroon	4.46	2.03	1.64		7.9	2.88	2.27	
Nigeria	3.68	1.38	1.08		9.07	3.95	5.26	
Egypt	4.9	2.76			9.25	8.52		
Benin	2.77	0.85	0.28		5.2	2.62	2.22	
Malawi	3.35	1.49	2.19		6.57	4.78	4.95	
Guinea	2.22	0.69	0.2		5.65	3.14	2.37	
Burkina Faso	1.07	0.23	0.05		3.67	1.56	0.45	
Liberia	2.51	1.73	0.64		5.07	3.42	2.19	
Mali	1.36	0.7	0.18		3.87	2.09	0.63	
Mozambique	1.43	0.92	1.54		3.16	2.23	3.41	
Ethiopia	0.84	0.38	0.16		2.91	1.47	0.61	
Mauritius	6.16	5.84			9.18	9.08		
Uganda	3.1	2.93	0.51		6.9	7.35	3.53	
Zambia	3.84	4.19	3.42		7.25	7.88	7.05	
Rwanda	1.66	2.14	0.27		4.76	5.84	5.62	
South Africa	5.89	8.05	2.2		10.55	10.72	9.78	
Botswana	3.51	9.44	1.29		10.6	11.01	8.51	

Table A.2: Country-Religious Group Years of Schooling

The table reports mean years of schooling for individuals aged 25+ for the 1940s and 1980s cohorts (because of the the Sierra Leone values correspond to the 1970s. Columns (1)-(3) show mean years of schooling for the 1940s, Columns (5)-(7) for the 1980s (or 1970s). Columns (4) and (8) show differences between Christians and Muslims. Red bars indicate a negative difference, green bars a positive difference.

Educational Dynamics. Examples SI Figure A.1 plots the evolution of the share of the population with completed primary education for different cohorts across all religious categories in Cameroon, Ethiopia, Mali, Nigeria, Senegal, and Uganda, looking at all individuals older than 14 years. The gap in completed primary education between Christians and Muslims at independence remains considerable in Nigeria for the post-1960s cohorts. In Senegal and Mali, where Muslims are about 95% of the population the Christian-Muslim gap in completed primary at independence slightly increases. If anything, the sizable religious educational gap at independence widens in Cameroon and Ethiopia, where Muslims are one-fifth and one-third of the population, respectively.

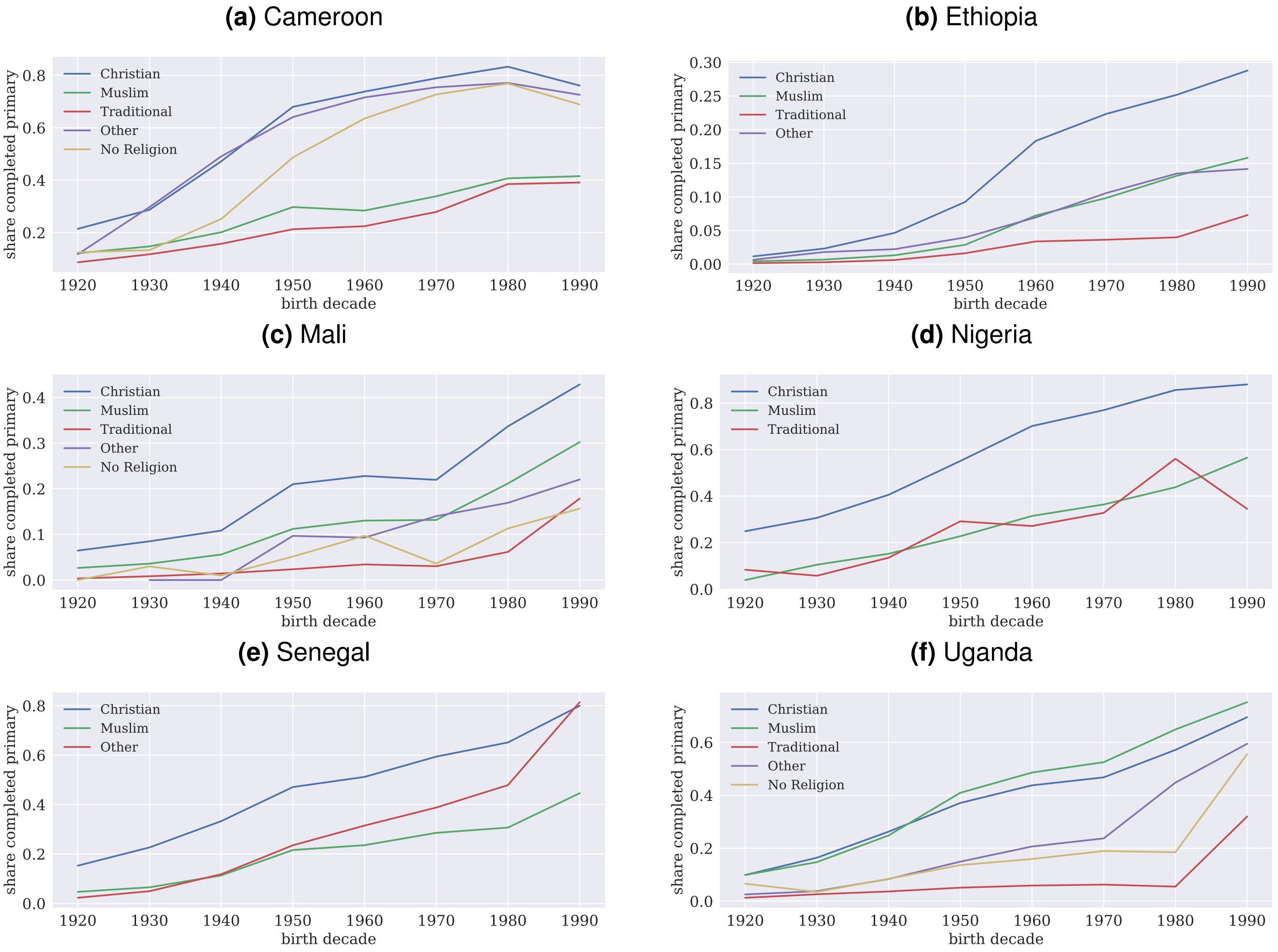

Figure A.1: Population Share with Primary Education Completed, by Birth Cohort, Individuals

The figures plot the share of the population older than 14 years with completed primary schooling, by religious affiliation across decade birth cohorts in Cameroon, Ethiopia, Mali, Nigeria, Senegal, and Uganda. The figures use all available Census information, as reported in IPUMS. Appendix Table 1 Extended Data reports the religion shares in the total population for the six countries.

A.3 Pan-African Patterns

SI Figure A.2 examines educational dynamics for Christians, Muslims, and Africans adhering to local religions pooling all observations from the 21 countries in IPUMS with information on religion. The figure plots the cumulative density function (CDF) for educational attainment (years of schooling) across cohorts for Christians, Muslims, and Traditionalists, using information for individuals older than 25 years. 65% of Christians born in the 1950s had not completed primary education (panel (b)); more than one in two Christians had not attended any schooling. The share of Christians with less than six years of schooling drops to 50% for Christians born in the 1970s and 40% for those born in the 1980s. Almost 80% of Muslims born in the 1950s had less than 6 years of education; most of them, 75%, had not attended any schooling. Despite the general increase in education, most Muslims born in the 1980s did not complete primary education (60%). Roughly 80% of Africans adhering to Traditional religions in the 1960s had not attended any schooling, and about 90% did not complete primary education. This share falls over time, as schooling expands. However, the share of Traditionalists, born in the 1980s, without completed primary education remains high at about 70%.

A.4 Regional Patterns

We then explore regional dynamics in education for Africans adhering to different religions. SI Figure A.3 plots the share with completed primary among the 1950s-born and the 1980s-born among Christians (panels (a)-(b)), Muslims (panels (c)-(d)), and Traditionalists (panels (e)-(f)) across more than 2,000 African districts in 21 countries. There are educational gains for all main religions. Improvements are, on average, larger in Southern Africa for all religious groups. Besides, gains for Muslims appear small in West Africa and large in Rwanda, Uganda, and Zambia.

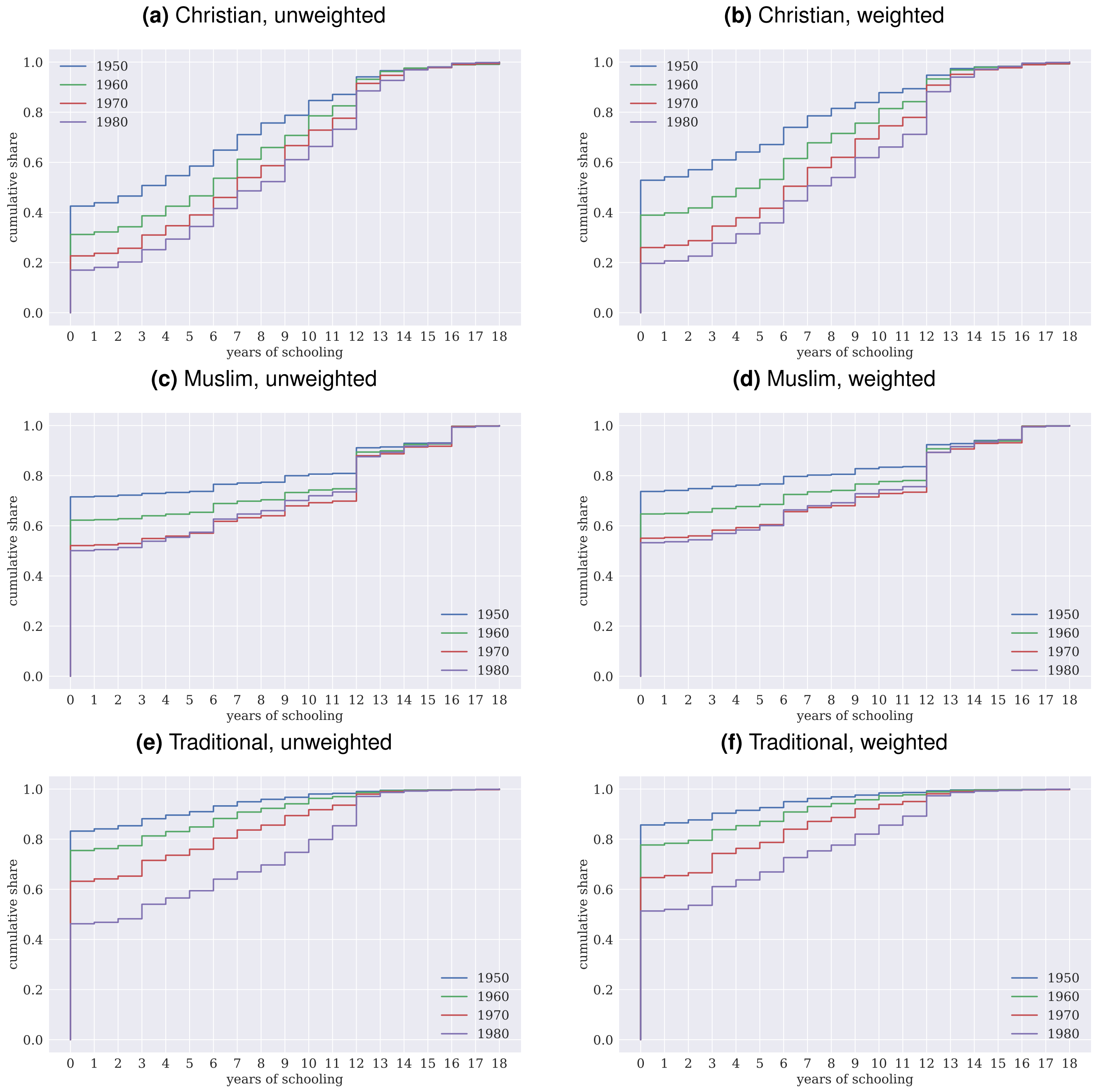

Figure A.2: Years of Schooling. CDF by Major Religion

The figures plot the cumulative density function (CDF) for years of schooling for Africans born in the 1950s, 1960s, 1970s, and 1980s, distinguishing between Christians (panels (a)-(b)), Muslims (panels (c)-(d)), and Africans adhering to Traditional religions, Traditionalists (panels (e)-(f)). The tabulations are based on all available Censuses, as reported in IPUMS for all individuals older than 25. Thus, the sample is unbalanced, as Census coverage is not even across cohorts in the 21-country sample. Panels (a), (c), and (e) report unweighted statistics. Panels (b), (d), and (f) weight observations by countries' 1980 population. Appendix Table B.1 gives sample details for all countries and censuses.

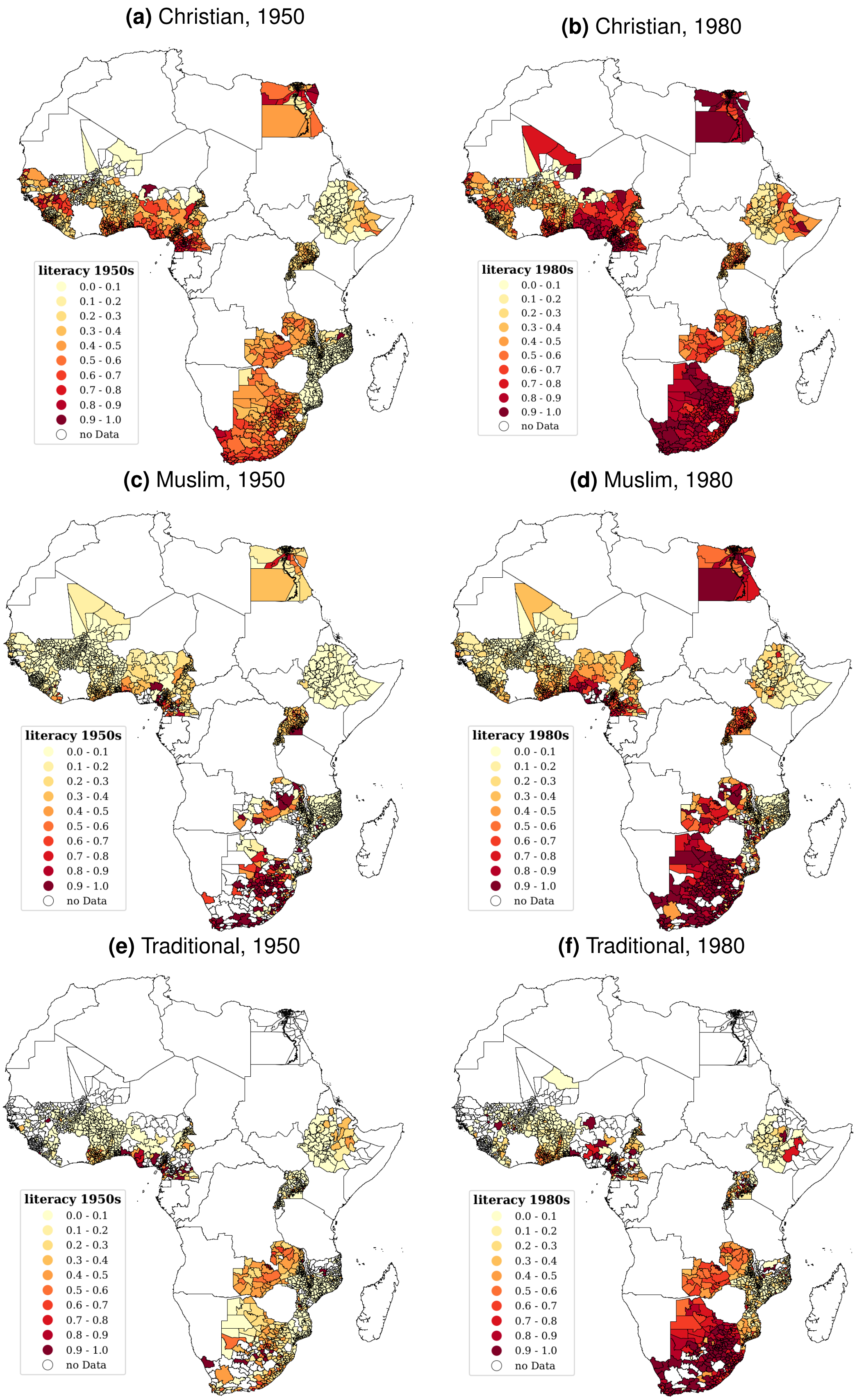

Figure A.3: Evolution of Completed Primary across African Regions by Religious Affiliation

The figures plot the share of Christians (panels (a)-(b)), the share of Muslims (panels (c)-(d)), and the share of Traditionalists (panels (e)-(f)) with completed primary for the 1950s-born and the 1980s-born across African admin-2/3 districts in 21 countries.

B Data and Sample

This Section complements the discussion on data in Section of the main paper. First, B.1 provides details on the sample construction. Second, B.2 reports statistics of co-residence rates of young individuals (aged 14-18 and 14-25) across African countries and districts for Africans adhering to different religions. Third, B.3 reports statistics on the intergenerational (children-parents) transmission in religious affiliation for Muslims, Traditionalists, and Christians. Fourth, B.4 discusses differences in religious affiliation across and within ethnic groups.

B.1 Sample Construction

SI Table B.1 gives the number of observations per census and country. The table also reports the number of districts and ethnic groups, as well as countries' population in 1980.

SI Table B.2 details how we build the sample used in the empirical analysis. The columns give the number of observations (individuals) starting from the IPUMS database, adding restrictions needed for the construction of the statistics of intergenerational mobility (IM) in education across religious lines. We need information on the individual's age, religious affiliation, educational attainment, residence, and household identifier, as well as the religion and education of at least one older generation member in the household.

SI Table B.3 gives details on the assignment of religious groups in IPUMS (Census) to the three main religions (Christians, Muslims, Traditionalists/Animists), plus the two auxiliary religious groups (No Religion and Other Religion) for each country.

		(1)	(2)	(3)	(4)	(5)	(6)	(7)
country	year	N^{14+}	N^{14-18}_{olded}	N^{14-25}_{olded}	$N^{14-25}_{\text{exposure}}$	N_{dist}	N_{eth}	pop$_{1980}$
Benin	1992	255,736	34,784	65,040	4,928	77	9	3,717,165
Benin	2002	373,452	57,364	104,331	7,795	77	9	3,717,165
Benin	2013	559,525	93,329	170,580	9,227	77	9	3,717,165
Botswana	2001	109,509	16,077	29,119		21	8	897,868
Botswana	2011	138,094	14,276	28,817		21	8	897,868
Burkina Faso	1996	552,062	95,669	157,808		45		6,822,843
Burkina Faso	2006	770,161	123,364	211,275		45	12	6,822,843
Cameroon	2005	1,003,327	162,672	295,388	14,650	204		8,621,406
Egypt	1986	4,261,935	693,275	1,345,068	40,708	173		43,309,063
Egypt	1996	3,810,835	695,795	1,230,963	16,798	231		43,309,063
Egypt	2006	5,096,618	759,450	1,590,965	16,599	235		43,309,063
Ethiopia	1984	1,798,345	234,160	359,917	10,193	57	14	35,141,712
Ethiopia	1994	2,831,290	498,229	787,544		80	14	35,141,712
Ethiopia	2007	744,744	128,818	200,774		89	14	35,141,712
Ghana	2000	1,152,128	167,556	309,485		110	9	11,056,116
Ghana	2010	1,575,528	229,128	424,323	18,908	110	9	11,056,116
Guinea	1983	274,948	33,789	68,385		33		4,871,435
Guinea	1996	396,987	58,373	113,585	8,851	33		4,871,435
Guinea	2014	602,197	108,289	212,496	18,275	33	10	4,871,435
Liberia	2008	210,111	32,411	60,197		42	14	1,853,001
Malawi	1998	582,694	83,465	131,703		183		6,250,440
Malawi	2008	736,175	108,154	170,362	10,281	183	9	6,250,440
Mali	2009	775,335	134,899	241,745	10,532	241	11	7,090,126
Mauritius	1990	76,358	9,632	21,222		39	6	966,039
Mauritius	2000	90,070	9,623	22,205		39	6	966,039
Mauritius	2011	99,864	9,667	20,783		39	6	966,039
Mozambique	2007	1,094,578	149,848	248,491		337	17	11,630,194
Nigeria	2010	41,753	6,846	12,376		37		73,423,633
Rwanda	1991	372,256	59,477	98,430	4,839	30	2	5,153,312
Rwanda	2002	456,450	87,049	139,236	5,234	30	6	5,153,312
Rwanda	2012	623,955	95,109	169,293		30	4	5,153,312
Senegal	1988	377,008	58,090	117,949		27	10	5,583,165
Senegal	2002	592,830	112,421	223,045		27	10	5,583,165
Senegal	2013	718,064	120,835	251,201		27	9	5,583,165
Sierra Leone	2004	291,916	48,263	92,052		100	12	3,388,495
South Africa	1996	2,085,501	280,980	542,636		179	11	28,556,769
South Africa	2001	2,562,379	347,662	665,466	4,496	179	11	28,556,769
South Africa	2016	2,337,822	250,101	528,915	241	206	11	28,556,769
Togo	1970	11,724	1,080	2,143		19	19	2,720,839
Togo	2010	339,447	46,328	85,981	6,688	37	20	2,720,839
Uganda	1991	855,005	128,871	205,895	13,425	133	21	12,442,334
Uganda	2002	1,355,857	223,066	337,594	12,752	136	21	12,442,334
Uganda	2014	1,960,887	352,962	545,329	17,513	137	21	12,442,334
Zambia	2000	570,022	100,981	173,281	15,723	55	18	5,851,825
Zambia	2010	704,471	126,500	205,511	8,030	55	18	5,851,825
total		46,229,953	7,188,717	13,018,904	276,686	2,286	222	278,381,741

Table B.1: Observations by Country and Census

This table reports the number of observations by census. Column (1) shows the total number of observations for individuals aged 14+ for whom we oberve religion as well as their own educational attainment. Column (2) shows the number of individuals aged 14-18 for whom we observe religion, their own educational attainment, as well as the educational attainment of the previous generation in the family. Column (3) is the same as column (2) but for individuals aged 14-25. Column (4) shows the number of individuals that enter in the exposure effects regressions. Column (5) shows the number of unique district in that census-year, column (6) shows the number of unique ethnic groups and column (7) shows the country's population in 1980, which we use for weighting.

country	year	full census fraction	(1) number of individuals in raw data	(2) number of individuals with religion observed	(3) number of individuals with religion + own age observed	(4) number of individuals with religion + own age + own education observed	(5) number of individuals, aged 14+, with religion + own age + own education observed	(6) number of individuals, aged 14-25, with religion + own age + own education observed	(7) number of individuals, aged 14-18, with religion + own age + own education observed	(8) number of individuals, aged 14+, with religion + own age + own education + previous generation education observed	(9) number of individuals, aged 14-25, with religion + own age + own education + previous generation education observed	(10) number of individuals, aged 14-18, with religion + own age + own education + previous generation education observed
Benin	1992	10.0	498,419	495,114	494,900	433,274	255,736	100,988	44,455	108,664	65,040	34,784
Benin	2002	10.0	685,467	685,467	685,467	612,658	373,452	155,832	69,048	160,458	104,331	57,364
Benin	2013	10.0	1,009,693	1,009,693	1,009,693	911,604	559,525	240,049	108,694	244,182	170,580	93,329
Burkina Faso	1996	10.0	1,081,046	1,025,717	1,021,722	802,832	552,062	226,254	114,088	250,828	157,808	95,669
Burkina Faso	2006	10.0	1,417,824	1,417,824	1,410,123	1,244,291	770,161	321,384	151,393	327,195	211,275	123,364
Botswana	2001	10.0	168,676	118,718	118,211	117,809	109,509	44,734	20,578	42,573	29,119	16,077
Botswana	2011	10.0	201,752	147,294	146,827	146,149	138,094	48,829	20,638	47,082	28,817	14,276
Cameroon	2005	10.0	1,772,359	1,747,716	1,747,716	1,524,571	1,003,327	431,550	197,049	426,755	295,388	162,672
Egypt	1986	14.1	6,799,093	6,797,805	6,793,098	5,417,612	4,261,935	1,609,560	721,960	1,931,312	1,345,068	693,275
Egypt	1996	10.0	5,902,243	5,902,243	5,901,839	4,453,382	3,810,835	1,471,285	718,874	1,616,808	1,230,963	695,795
Egypt	2006	10.0	7,282,434	7,282,434	7,282,434	5,739,722	5,096,618	1,977,932	785,619	2,046,232	1,590,965	759,450
Ethiopia	1984	10.0	3,404,306	3,365,378	3,360,536	2,729,932	1,798,345	618,869	303,442	556,109	359,917	234,160
Ethiopia	1994	10.0	5,044,598	5,006,937	5,006,936	4,198,638	2,831,290	1,223,914	613,743	1,033,511	787,544	498,229
Ethiopia	2007	10.0	7,434,086	7,434,086	7,434,086	1,097,614	744,744	331,544	161,226	259,645	200,774	128,818
Ghana	2000	10.0	1,894,133	1,894,133	1,894,133	1,730,902	1,152,128	434,882	200,000	489,201	309,485	167,556
Ghana	2010	10.0	2,466,289	2,466,289	2,466,289	2,262,894	1,575,528	603,020	270,162	636,999	424,323	229,128
Guinea	1983	10.0	457,837	457,477	457,474	364,655	274,948	99,777	44,104	120,685	68,385	33,789
Guinea	1996	10.0	729,071	715,336	713,606	551,391	396,987	148,010	69,146	202,097	113,585	58,373
Guinea	2014	10.0	1,050,916	1,050,916	1,050,916	951,617	602,197	250,819	118,361	355,811	212,496	108,289
Liberia	2008	10.0	348,057	348,057	348,057	294,517	210,111	87,459	38,854	86,523	60,197	32,411
Mali	2009	10.0	1,451,856	1,447,559	1,422,097	1,260,437	775,335	325,596	158,204	366,117	241,745	134,899
Mozambique	2007	10.0	2,047,048	2,001,190	2,001,190	1,606,077	1,094,578	434,683	192,075	337,181	248,491	149,848
Mauritius	1990	10.0	106,710	106,013	106,000	101,625	76,358	24,399	9,947	34,593	21,222	9,632
Mauritius	2000	10.0	119,695	118,440	118,432	114,249	90,070	25,401	9,834	37,403	22,205	9,623
Mauritius	2011	10.0	126,332	125,164	125,077	121,251	99,864	22,998	9,879	38,620	20,783	9,667
Malawi	1998	10.0	991,393	991,393	991,393	826,197	582,694	251,873	114,846	170,361	131,703	83,465
Malawi	2008	10.0	1,341,977	1,302,899	1,301,989	1,161,773	736,175	307,167	135,833	216,020	170,362	108,154
Nigeria	2010	0.05	72,191	71,839	71,835	58,859	41,753	15,457	7,521	17,701	12,376	6,846
Rwanda	1991	10.0	742,918	715,704	715,704	535,452	372,256	146,797	71,270	121,728	98,430	59,477
Rwanda	2002	10.0	843,392	792,433	792,433	611,066	456,450	216,930	108,559	159,872	139,236	87,049
Rwanda	2012	10.0	1,038,369	1,036,233	1,036,233	937,974	623,955	250,111	112,241	206,403	169,293	95,109
Senegal	1988	10.0	700,199	690,372	690,157	525,544	377,008	152,934	68,710	199,854	117,949	58,090
Senegal	2002	10.0	994,562	990,796	990,796	909,126	592,830	259,440	124,289	373,031	223,045	112,421
Senegal	2013	10.0	1,245,551	1,189,111	1,189,110	1,091,087	718,064	285,038	131,056	456,163	251,201	120,835
Sierra Leone	2004	10.0	494,298	494,298	492,922	395,788	291,916	120,773	55,346	148,389	92,052	48,263
Togo	1970	1.0	23,680	22,648	22,593	22,587	11,724	3,980	1,574	4,044	2,143	1,080
Togo	2010	10.0	584,859	584,859	584,859	517,900	339,447	132,399	58,429	131,016	85,981	46,328
Uganda	1991	10.0	1,548,460	1,547,236	1,546,543	1,242,140	855,005	378,293	179,153	282,113	205,895	128,871
Uganda	2002	10.0	2,497,449	2,497,449	2,497,449	2,042,838	1,355,857	601,101	289,123	438,634	337,594	223,066
Uganda	2014	10.0	3,506,546	3,506,546	3,506,546	3,145,894	1,960,887	860,666	423,773	692,614	545,329	352,962
South Africa	1996	10.0	3,621,164	3,226,462	3,194,244	2,745,697	2,085,501	744,760	335,699	807,390	542,636	280,980
South Africa	2001	10.0	3,725,655	3,675,739	3,675,739	3,308,325	2,562,379	902,662	415,372	1,002,640	665,466	347,662
South Africa	2016	5.8	3,328,793	3,288,741	3,288,741	3,243,841	2,337,822	711,443	298,203	893,897	528,915	250,101
Zambia	2000	10.0	996,117	996,117	996,117	825,110	570,022	259,096	119,089	230,361	173,281	100,981
Zambia	2010	10.0	1,321,973	1,249,689	1,249,689	1,028,628	704,471	307,786	147,933	263,135	205,511	126,500
total			83,119,486	82,037,564	81,951,951	63,965,529	46,229,953	18,168,474	8,349,392	18,571,950	13,018,904	7,188,717

Table B.2: Sample Construction

The table details how we proceed from the original IPUMS data to the sample we use in the analysis for each country and census year; the table also reports the fraction of the census obtained, processed and harmonized by IPUMS, typically 10%. Column (1) gives the number of observations in the IPUMS database. Column (2) gives the number of observations with information on the individual's religion. Column (3) gives the number of observations for which IPUMS reports both age and religion. Column (4) gives the number of observations for which IPUMS reports age, religion, and education. Column (5) gives the number of observations/individuals, older than 14 years old with available information on age, religion, and education. Column (6) gives the number of observations/individuals in the 14-25 age range with information on age, religion, and education, while column (7) reports the corresponding number for individuals, aged 14-18. Columns (8), (9), and (10) are similar to (5)-(7), but require that IPUMS reports the age and education of at least one member of the older generation in the household, needed to compile measures of intergenerational mobility (IM) in educational attainment.

Table B.3: Religious groups by Country and Size

(1) detailed religion name, IPUMS	(2) number of observations in religion	(3) group share, religion	(4) number of observations in religion with education of the old observed	(5) group share, religion with education of the old observed	(6) major religion name	(7) number of observations in major religion	(8) group share, major religion	(9) number of observations in major religion with education of the old observed	(10) group share, major religion with education of the old observed
**Benin**									
Catholic (Roman or Unspecified)	577,888	0.264	371,192	0.254	Christian	968,403	0.442	628,906	0.430
Other Christian	163,733	0.075	107,398	0.074	Christian	968,403	0.442	628,906	0.430
Celestial	102,365	0.047	68,450	0.047	Christian	968,403	0.442	628,906	0.430
Methodist	74,330	0.034	48,582	0.033	Christian	968,403	0.442	628,906	0.430
Protestant	50,087	0.023	33,284	0.023	Christian	968,403	0.442	628,906	0.430
Muslim	555,388	0.254	398,228	0.273	Muslim	555,388	0.254	398,228	0.273
Traditional	244,198	0.111	160,749	0.110	Traditional	481,689	0.220	312,064	0.214
Vodoun	237,491	0.108	151,315	0.104	Traditional	481,689	0.220	312,064	0.214
No Religion	136,384	0.062	90,136	0.062	No Religion	136,384	0.062	90,136	0.062
Other, Benin	48,410	0.022	31,626	0.022	Other	48,410	0.022	31,626	0.022
**Burkina Faso**									
Muslim	1,433,923	0.587	968,081	0.588	Muslim	1,433,923	0.587	968,081	0.588
Catholic (Roman or Unspecified)	438,746	0.180	292,017	0.177	Christian	529,025	0.216	350,619	0.213
Protestant	90,279	0.037	58,602	0.036	Christian	529,025	0.216	350,619	0.213
Animist	459,250	0.188	316,234	0.192	Traditional	459,250	0.188	316,234	0.192
No Religion	11,306	0.005	7,624	0.005	No Religion	11,306	0.005	7,624	0.005
Other, not Elsewhere Classified	10,037	0.004	5,235	0.003	Other	10,037	0.004	5,235	0.003
**Botswana**									
Christian	202,249	0.760	79,594	0.764	Christian	202,249	0.760	79,594	0.764
No Religion	47,019	0.177	19,738	0.190	No Religion	47,019	0.177	19,738	0.190
Badimo	13,047	0.049	3,621	0.035	Traditional	13,047	0.049	3,621	0.035
Other, Botswana	902	0.003	296	0.003	Other	2,017	0.008	585	0.006
Hindu	640	0.002	157	0.002	Other	2,017	0.008	585	0.006
Bahai	257	0.001	69	0.001	Other	2,017	0.008	585	0.006
Rastafarian	218	0.001	63	0.001	Other	2,017	0.008	585	0.006
Muslim	1,680	0.006	577	0.006	Muslim	1,680	0.006	577	0.006
**Cameroon**									
Catholic (Roman or Unspecified)	671,218	0.384	426,778	0.377	Christian	1,210,224	0.692	771,236	0.680
Protestant	458,789	0.263	295,110	0.260	Christian	1,210,224	0.692	771,236	0.680
Other Christian, Cameroon	71,185	0.041	43,661	0.039	Christian	1,210,224	0.692	771,236	0.680
Orthodox	9,032	0.005	5,687	0.005	Christian	1,210,224	0.692	771,236	0.680
Muslim	365,421	0.209	252,457	0.223	Muslim	365,421	0.209	252,457	0.223
Animist	97,518	0.056	66,139	0.058	Traditional	97,518	0.056	66,139	0.058
No Religion	57,005	0.033	32,885	0.029	No Religion	57,005	0.033	32,885	0.029
Other, not Elsewhere Classified	17,548	0.010	10,784	0.010	Other	17,548	0.010	10,784	0.010
**Egypt**									
Muslim	18,856,525	0.944	11,709,350	0.947	Muslim	18,856,525	0.944	11,709,350	0.947
Christian	1,124,532	0.056	659,096	0.053	Christian	1,124,532	0.056	659,096	0.053
Other	932	0.000	34	0.000	Other	1,425	0.000	231	0.000
Jewish	406	0.000	170	0.000	Other	1,425	0.000	231	0.000
Other, Egypt	87	0.000	27	0.000	Other	1,425	0.000	231	0.000
**Ethiopia**									
Orthodox	7,854,225	0.497	3,107,149	0.523	Christian	10,109,485	0.640	3,795,524	0.639
Protestant	2,120,593	0.134	631,803	0.106	Christian	10,109,485	0.640	3,795,524	0.639
Catholic (Roman or Unspecified)	134,667	0.009	56,572	0.010	Christian	10,109,485	0.640	3,795,524	0.639
Muslim	4,919,812	0.311	1,791,583	0.301	Muslim	4,919,812	0.311	1,791,583	0.301
Traditional	622,923	0.039	282,753	0.048	Traditional	622,923	0.039	282,753	0.048
Other, Ethiopia	154,181	0.010	72,564	0.012	Other	154,181	0.010	72,564	0.012
**Ghana**									
Pentecostal	1,153,222	0.264	700,651	0.263	Christian	3,057,276	0.701	1,845,319	0.692
Protestant	807,571	0.185	474,277	0.178	Christian	3,057,276	0.701	1,845,319	0.692
Catholic (Roman or Unspecified)	608,309	0.140	374,217	0.140	Christian	3,057,276	0.701	1,845,319	0.692
Other Christian, Ghana	488,174	0.112	296,174	0.111	Christian	3,057,276	0.701	1,845,319	0.692

Table B.3: Religious Groups by Country and Size, continued

(1) detailed religion name, IPUMS	(2) number of observations in religion	(3) group share, religion	(4) number of observations in religion with education of the old observed	(5) group share, religion with education of the old observed	(6) major religion name	(7) number of observations in major religion	(8) group share, major religion	(9) number of observations in major religion with education of the old observed	(10) group share, major religion with education of the old observed
Muslim	722,897	0.166	482,403	0.181	Muslim	737,951	0.169	491,849	0.184
Ahmadis	15,054	0.003	9,446	0.004	Muslim	737,951	0.169	491,849	0.184
Traditional, Ghana	287,582	0.066	185,173	0.069	Traditional	287,582	0.066	185,173	0.069
No Religion	244,706	0.056	125,429	0.047	No Religion	244,706	0.056	125,429	0.047
Other, Ghana	19,596	0.004	11,054	0.004	Other	32,907	0.008	18,820	0.007
Other, not Elsewhere Classified	13,311	0.003	7,766	0.003	Other	32,907	0.008	18,820	0.007
**Guinea**									
Muslim	1,956,921	0.880	1,393,724	0.886	Muslim	1,956,921	0.880	1,393,724	0.886
Christian	117,683	0.053	81,578	0.052	Christian	137,412	0.062	93,267	0.059
Catholic (Roman or Unspecified)	16,387	0.007	9,648	0.006	Christian	137,412	0.062	93,267	0.059
Protestant	3,169	0.001	1,927	0.001	Christian	137,412	0.062	93,267	0.059
Anglican	173	0.000	114	0.000	Christian	137,412	0.062	93,267	0.059
No Religion	72,854	0.033	51,597	0.033	No Religion	72,854	0.033	51,597	0.033
Animist	51,887	0.023	31,800	0.020	Traditional	51,887	0.023	31,800	0.020
Other, Guinea	3,384	0.002	2,227	0.001	Other	4,655	0.002	3,105	0.002
Other, not Elsewhere Classified	1,271	0.001	878	0.001	Other	4,655	0.002	3,105	0.002
**Liberia**									
Christian	298,471	0.858	187,121	0.866	Christian	298,471	0.858	187,121	0.866
Muslim	41,965	0.121	24,657	0.114	Muslim	41,965	0.121	24,657	0.114
No Religion	5,034	0.014	2,932	0.014	No Religion	5,034	0.014	2,932	0.014
Traditional	2,060	0.006	1,014	0.005	Traditional	2,060	0.006	1,014	0.005
Other, Liberia	527	0.002	285	0.001	Other	527	0.002	285	0.001
**Mali**									
Muslim	1,376,478	0.951	961,193	0.952	Muslim	1,376,478	0.951	961,193	0.952
Christian	34,667	0.024	22,776	0.023	Christian	34,667	0.024	22,776	0.023
Animist	29,437	0.020	19,583	0.019	Traditional	29,437	0.020	19,583	0.019
No Religion	6,409	0.004	5,202	0.005	No Religion	6,409	0.004	5,202	0.005
Other, Mali	568	0.000	393	0.000	Other	568	0.000	393	0.000
**Mozambique**									
Catholic (Roman or Unspecified)	571,694	0.286	337,773	0.280	Christian	1,127,762	0.564	694,057	0.576
Zion Christian	311,048	0.155	200,554	0.166	Christian	1,127,762	0.564	694,057	0.576
Pentecostal	218,623	0.109	139,585	0.116	Christian	1,127,762	0.564	694,057	0.576
Anglican	26,397	0.013	16,145	0.013	Christian	1,127,762	0.564	694,057	0.576
No Religion	378,807	0.189	228,148	0.189	No Religion	378,807	0.189	228,148	0.189
Muslim	360,001	0.180	200,415	0.166	Muslim	360,001	0.180	200,415	0.166
Other, not Elsewhere Classified	134,620	0.067	82,777	0.069	Traditional	134,620	0.067	82,777	0.069
**Mauritius**									
Hindu	173,516	0.496	97,502	0.509	Other	175,795	0.503	98,199	0.512
Buddhist	1,699	0.005	415	0.002	Other	175,795	0.503	98,199	0.512
Bahai	295	0.001	163	0.001	Other	175,795	0.503	98,199	0.512
Other, not Elsewhere Classified	285	0.001	119	0.001	Other	175,795	0.503	98,199	0.512
Catholic (Roman or Unspecified)	89,791	0.257	48,234	0.252	Christian	113,336	0.324	60,646	0.316
Christian	15,452	0.044	8,170	0.043	Christian	113,336	0.324	60,646	0.316
Assembly of God	2,632	0.008	1,438	0.008	Christian	113,336	0.324	60,646	0.316
Adventist / Seventh-Day Adventist	1,142	0.003	597	0.003	Christian	113,336	0.324	60,646	0.316
Anglican	1,140	0.003	543	0.003	Christian	113,336	0.324	60,646	0.316
Pentecostal	1,103	0.003	605	0.003	Christian	113,336	0.324	60,646	0.316
Jehovah's Witnesses	599	0.002	313	0.002	Christian	113,336	0.324	60,646	0.316
Salvation and Healing Mission	589	0.002	303	0.002	Christian	113,336	0.324	60,646	0.316
Other Christian	456	0.001	221	0.001	Christian	113,336	0.324	60,646	0.316
Presbyterian	199	0.001	105	0.001	Christian	113,336	0.324	60,646	0.316
Evangelical Protestant	75	0.000	39	0.000	Christian	113,336	0.324	60,646	0.316
Christian Tamil	65	0.000	42	0.000	Christian	113,336	0.324	60,646	0.316
Voice of Deliverance	50	0.000	25	0.000	Christian	113,336	0.324	60,646	0.316
Protestant	43	0.000	11	0.000	Christian	113,336	0.324	60,646	0.316

Table B.3: Religious Groups by Country and Size, continued

(1) detailed religion name, IPUMS	(2) number of observations in religion	(3) group share, religion	(4) number of observations in religion with education of the old observed	(5) group share, religion with education of the old observed	(6) major religion name	(7) number of observations in major religion	(8) group share, major religion	(9) number of observations in major religion with education of the old observed	(10) group share, major religion with education of the old observed
Muslim	58,808	0.168	32,258	0.168	Muslim	58,808	0.168	32,258	0.168
No Religion	1,678	0.005	619	0.003	No Religion	1,678	0.005	619	0.003
**Malawi**									
Christian	1,867,448	0.814	1,081,494	0.829	Christian	1,867,448	0.814	1,081,494	0.829
Muslim	296,822	0.129	164,035	0.126	Muslim	296,822	0.129	164,035	0.126
No Religion	74,816	0.033	28,665	0.022	No Religion	74,816	0.033	28,665	0.022
Other, not Elsewhere Classified	55,206	0.024	29,934	0.023	Traditional	55,206	0.024	29,934	0.023
**Nigeria**									
Christian	37,715	0.525	22,907	0.548	Christian	37,715	0.525	22,907	0.548
Muslim	33,461	0.466	18,616	0.445	Muslim	33,461	0.466	18,616	0.445
Traditional	653	0.009	307	0.007	Traditional	653	0.009	307	0.007
Other, Nigeria	10	0.000	4	0.000	Other	10	0.000	4	0.000
**Rwanda**									
Catholic (Roman or Unspecified)	1,300,981	0.511	777,414	0.515	Christian	2,372,408	0.932	1,408,411	0.934
Protestant	746,937	0.294	442,287	0.293	Christian	2,372,408	0.932	1,408,411	0.934
Adventist / Seventh-Day Adventist	281,549	0.111	163,319	0.108	Christian	2,372,408	0.932	1,408,411	0.934
Other Christian, Rwanda 2002	32,309	0.013	19,246	0.013	Christian	2,372,408	0.932	1,408,411	0.934
Jehovah's Witnesses	10,632	0.004	6,145	0.004	Christian	2,372,408	0.932	1,408,411	0.934
No Religion	103,219	0.041	62,913	0.042	No Religion	103,219	0.041	62,913	0.042
Muslim	44,574	0.018	25,465	0.017	Muslim	44,574	0.018	25,465	0.017
Other, Rwanda	15,859	0.006	8,983	0.006	Other	15,859	0.006	8,983	0.006
Traditional Religion, Rwanda	8,310	0.003	2,483	0.002	Traditional	8,310	0.003	2,483	0.002
**Senegal**									
Muslim	2,742,582	0.956	2,083,495	0.961	Muslim	2,742,582	0.956	2,083,495	0.961
Catholic (Roman or Unspecified)	110,823	0.039	74,278	0.034	Christian	117,194	0.041	78,134	0.036
Protestant	3,320	0.001	1,977	0.001	Christian	117,194	0.041	78,134	0.036
Other Christian	3,051	0.001	1,879	0.001	Christian	117,194	0.041	78,134	0.036
Other	10,056	0.004	5,798	0.003	Other	10,056	0.004	5,798	0.003
No Religion	447	0.000	234	0.000	No Religion	447	0.000	234	0.000
**Sierra Leone**									
Sunni	261,294	0.529	178,129	0.529	Muslim	378,977	0.767	256,836	0.763
Muslim	92,641	0.187	61,921	0.184	Muslim	378,977	0.767	256,836	0.763
Ahmadis	25,042	0.051	16,786	0.050	Muslim	378,977	0.767	256,836	0.763
Catholic (Roman or Unspecified)	35,298	0.071	24,285	0.072	Christian	104,272	0.211	70,570	0.210
Other Christian, Sierra Leone	22,781	0.046	15,599	0.046	Christian	104,272	0.211	70,570	0.210
Methodist	19,357	0.039	12,924	0.038	Christian	104,272	0.211	70,570	0.210
Pentecostal	16,862	0.034	11,131	0.033	Christian	104,272	0.211	70,570	0.210
Anglican	6,244	0.013	4,049	0.012	Christian	104,272	0.211	70,570	0.210
Adventist / Seventh-Day Adventist	3,730	0.008	2,582	0.008	Christian	104,272	0.211	70,570	0.210
No Religion	6,530	0.013	6,097	0.018	No Religion	6,530	0.013	6,097	0.018
Other, Sierra Leone	3,830	0.008	2,598	0.008	Other	4,212	0.009	2,871	0.009
Bahai	382	0.001	273	0.001	Other	4,212	0.009	2,871	0.009
Traditional Religion, Sierra Leone	307	0.001	198	0.001	Traditional	307	0.001	198	0.001
**Togo**									
Catholic (Roman or Unspecified)	152,168	0.250	87,684	0.238	Christian	291,160	0.479	169,187	0.459
Assembly of God	38,167	0.063	22,882	0.062	Christian	291,160	0.479	169,187	0.459
Other Christian, Togo	35,366	0.058	20,751	0.056	Christian	291,160	0.479	169,187	0.459
Presbyterian	27,265	0.045	14,844	0.040	Christian	291,160	0.479	169,187	0.459
Pentecostal	19,456	0.032	11,996	0.033	Christian	291,160	0.479	169,187	0.459
Baptist	10,190	0.017	6,204	0.017	Christian	291,160	0.479	169,187	0.459
Jehovah's Witnesses	4,453	0.007	2,440	0.007	Christian	291,160	0.479	169,187	0.459
Methodist	1,679	0.003	992	0.003	Christian	291,160	0.479	169,187	0.459
Protestant	1,315	0.002	794	0.002	Christian	291,160	0.479	169,187	0.459
Adventist / Seventh-Day Adventist	1,101	0.002	600	0.002	Christian	291,160	0.479	169,187	0.459

Table B.3: Religious Groups by Country and Size, continued

(1) detailed religion name, IPUMS	(2) number of observations in religion	(3) group share, religion	(4) number of observations in religion with education of the old observed	(5) group share, religion with education of the old observed	(6) major religion name	(7) number of observations in major religion	(8) group share, major religion	(9) number of observations in major religion with education of the old observed	(10) group share, major religion with education of the old observed
Animist	175,965	0.290	111,493	0.303	Traditional	175,965	0.290	111,493	0.303
Muslim	95,386	0.157	61,656	0.167	Muslim	95,386	0.157	61,656	0.167
without Religion	39,417	0.065	22,901	0.062	No Religion	39,417	0.065	22,901	0.062
Other, not Elsewhere Classified	5,579	0.009	3,255	0.009	Other	5,579	0.009	3,255	0.009
**Uganda**									
Catholic (Roman or Unspecified)	3,117,529	0.413	1,862,515	0.405	Christian	6,431,866	0.852	3,892,654	0.846
Anglican	2,630,739	0.348	1,607,853	0.350	Christian	6,431,866	0.852	3,892,654	0.846
Pentecostal	501,697	0.066	310,767	0.068	Christian	6,431,866	0.852	3,892,654	0.846
Adventist / Seventh-Day Adventist	114,723	0.015	69,751	0.015	Christian	6,431,866	0.852	3,892,654	0.846
Other Christian	40,592	0.005	25,070	0.005	Christian	6,431,866	0.852	3,892,654	0.846
Baptist	10,646	0.001	6,904	0.002	Christian	6,431,866	0.852	3,892,654	0.846
Orthodox	8,739	0.001	5,320	0.001	Christian	6,431,866	0.852	3,892,654	0.846
Salvation Army	2,728	0.000	1,726	0.000	Christian	6,431,866	0.852	3,892,654	0.846
Presbyterian	2,287	0.000	1,470	0.000	Christian	6,431,866	0.852	3,892,654	0.846
Jehovah's Witnesses	2,186	0.000	1,278	0.000	Christian	6,431,866	0.852	3,892,654	0.846
Muslim	934,319	0.124	586,754	0.128	Muslim	934,319	0.124	586,754	0.128
Other, Uganda	60,831	0.008	40,869	0.009	Other	120,122	0.016	78,502	0.017
Other Non-Christian, Uganda	51,053	0.007	32,819	0.007	Other	120,122	0.016	78,502	0.017
Bahai	4,828	0.001	3,011	0.001	Other	120,122	0.016	78,502	0.017
Hindu	1,527	0.000	661	0.000	Other	120,122	0.016	78,502	0.017
Mammon	860	0.000	523	0.000	Other	120,122	0.016	78,502	0.017
Jewish	764	0.000	486	0.000	Other	120,122	0.016	78,502	0.017
Buddhist	259	0.000	133	0.000	Other	120,122	0.016	78,502	0.017
Traditional Religion	34,780	0.005	21,589	0.005	Traditional	34,780	0.005	21,589	0.005
No Religion	30,144	0.004	20,212	0.004	No Religion	30,144	0.004	20,212	0.004
**South Africa**									
Zion Christian	1,108,838	0.109	656,513	0.119	Christian	7,956,947	0.781	4,392,912	0.794
Other African Independent Churches	986,883	0.097	567,741	0.103	Christian	7,956,947	0.781	4,392,912	0.794
Catholic (Roman or Unspecified)	779,179	0.076	414,330	0.075	Christian	7,956,947	0.781	4,392,912	0.794
Other Apostolic Churches	695,215	0.068	401,492	0.073	Christian	7,956,947	0.781	4,392,912	0.794
Methodist	677,499	0.066	356,466	0.064	Christian	7,956,947	0.781	4,392,912	0.794
Dutch Reformed	585,613	0.057	266,955	0.048	Christian	7,956,947	0.781	4,392,912	0.794
Pentecostal	517,318	0.051	291,477	0.053	Christian	7,956,947	0.781	4,392,912	0.794
Anglican	385,741	0.038	196,896	0.036	Christian	7,956,947	0.781	4,392,912	0.794
Other Christian Churches	353,776	0.035	197,601	0.036	Christian	7,956,947	0.781	4,392,912	0.794
Lutheran	248,328	0.024	137,390	0.025	Christian	7,956,947	0.781	4,392,912	0.794
Presbyterian	174,801	0.017	94,642	0.017	Christian	7,956,947	0.781	4,392,912	0.794
Baptist	158,671	0.016	87,579	0.016	Christian	7,956,947	0.781	4,392,912	0.794
Reformed	149,288	0.015	70,555	0.013	Christian	7,956,947	0.781	4,392,912	0.794
Other Evangelical Churches	143,176	0.014	85,705	0.015	Christian	7,956,947	0.781	4,392,912	0.794
Apostolic Faith Mission of Sa	120,123	0.012	66,273	0.012	Christian	7,956,947	0.781	4,392,912	0.794
Ethiopian Type Churches	105,359	0.010	61,461	0.011	Christian	7,956,947	0.781	4,392,912	0.794
Other Pentecostal Churches	95,245	0.009	56,109	0.010	Christian	7,956,947	0.781	4,392,912	0.794
Congregational	80,429	0.008	46,887	0.008	Christian	7,956,947	0.781	4,392,912	0.794
Jehovah's Witnesses	64,194	0.006	34,491	0.006	Christian	7,956,947	0.781	4,392,912	0.794
St John's Apostolic Church	63,852	0.006	37,168	0.007	Christian	7,956,947	0.781	4,392,912	0.794
Assembly of God	63,659	0.006	37,153	0.007	Christian	7,956,947	0.781	4,392,912	0.794
Bandla Lama Nazaretha	60,360	0.006	36,780	0.007	Christian	7,956,947	0.781	4,392,912	0.794
Adventist / Seventh-Day Adventist	52,660	0.005	28,365	0.005	Christian	7,956,947	0.781	4,392,912	0.794
African Methodist Episcopal Church	49,301	0.005	27,824	0.005	Christian	7,956,947	0.781	4,392,912	0.794
Full Gospel Church of God in SA	46,949	0.005	26,502	0.005	Christian	7,956,947	0.781	4,392,912	0.794
Christian Centres	33,739	0.003	19,620	0.004	Christian	7,956,947	0.781	4,392,912	0.794
International Pentecost Church	31,345	0.003	18,649	0.003	Christian	7,956,947	0.781	4,392,912	0.794
New Apostolic	20,901	0.002	11,760	0.002	Christian	7,956,947	0.781	4,392,912	0.794
Other Assemblies	20,375	0.002	12,444	0.002	Christian	7,956,947	0.781	4,392,912	0.794
Ethnic Churches	13,430	0.001	8,287	0.001	Christian	7,956,947	0.781	4,392,912	0.794
Pinkster Protestant Church	10,513	0.001	5,442	0.001	Christian	7,956,947	0.781	4,392,912	0.794
Int'l Fellowship of Chr. Churches	10,309	0.001	4,907	0.001	Christian	7,956,947	0.781	4,392,912	0.794
Salvation Army	9,436	0.001	5,645	0.001	Christian	7,956,947	0.781	4,392,912	0.794

Table B.3: Religious Groups by Country and Size, continued

(1) detailed religion name, IPUMS	(2) number of observations in religion	(3) group share, religion	(4) number of observations in religion with education of the old observed	(5) group share, religion with education of the old observed	(6) major religion name	(7) number of observations in major religion	(8) group share, major religion	(9) number of observations in major religion with education of the old observed	(10) group share, major religion with education of the old observed
Latter Day Saints (Mormon)	9,147	0.001	4,702	0.001	Christian	7,956,947	0.781	4,392,912	0.794
Pentecostal Churches	8,133	0.001	4,874	0.001	Christian	7,956,947	0.781	4,392,912	0.794
Other Catholic Churches	7,207	0.001	4,271	0.001	Christian	7,956,947	0.781	4,392,912	0.794
Other Orthodox Churches	4,627	0.000	2,512	0.000	Christian	7,956,947	0.781	4,392,912	0.794
Other African Apostolic Churches	3,714	0.000	2,237	0.000	Christian	7,956,947	0.781	4,392,912	0.794
Afrikaanse Protestant Church	3,274	0.000	1,271	0.000	Christian	7,956,947	0.781	4,392,912	0.794
Other Charismatic Churches	1,888	0.000	906	0.000	Christian	7,956,947	0.781	4,392,912	0.794
Orthodox	1,865	0.000	745	0.000	Christian	7,956,947	0.781	4,392,912	0.794
Christian Scientist	587	0.000	285	0.000	Christian	7,956,947	0.781	4,392,912	0.794
No Religion	964,498	0.095	493,643	0.089	No Religion	1,331,600	0.131	678,085	0.123
without Religion	363,579	0.036	182,983	0.033	No Religion	1,331,600	0.131	678,085	0.123
Atheist	2,189	0.000	913	0.000	No Religion	1,331,600	0.131	678,085	0.123
Agnostic	1,334	0.000	546	0.000	No Religion	1,331,600	0.131	678,085	0.123
Other	361,868	0.036	182,675	0.033	Other	587,064	0.058	295,479	0.053
Hindu	120,536	0.012	62,456	0.011	Other	587,064	0.058	295,479	0.053
Other Non-Christian, S. Africa	85,695	0.008	42,953	0.008	Other	587,064	0.058	295,479	0.053
Jewish	13,783	0.001	4,935	0.001	Other	587,064	0.058	295,479	0.053
Other, South Africa	2,083	0.000	1,140	0.000	Other	587,064	0.058	295,479	0.053
Buddhist	1,615	0.000	659	0.000	Other	587,064	0.058	295,479	0.053
Bahai	682	0.000	330	0.000	Other	587,064	0.058	295,479	0.053
Taoist	420	0.000	162	0.000	Other	587,064	0.058	295,479	0.053
New Age	263	0.000	111	0.000	Other	587,064	0.058	295,479	0.053
Confucian	119	0.000	58	0.000	Other	587,064	0.058	295,479	0.053
African Traditional Belief	171,071	0.017	86,623	0.016	Traditional	171,071	0.017	86,623	0.016
Muslim	144,260	0.014	77,726	0.014	Muslim	144,260	0.014	77,726	0.014
**Zambia**									
Protestant	1,583,933	0.705	1,020,868	0.712	Christian	2,059,553	0.917	1,320,268	0.921
Catholic (Roman or Unspecified)	475,620	0.212	299,400	0.209	Christian	2,059,553	0.917	1,320,268	0.921
Other, Zambia	97,263	0.043	61,713	0.043	Traditional	97,263	0.043	61,713	0.043
No Religion	76,485	0.034	44,420	0.031	No Religion	76,485	0.034	44,420	0.031
Muslim	10,282	0.005	6,333	0.004	Muslim	10,282	0.005	6,333	0.004
Hindu	991	0.000	510	0.000	Other	2,223	0.001	1,245	0.001
Buddhist	905	0.000	553	0.000	Other	2,223	0.001	1,245	0.001
Bahai	327	0.000	182	0.000	Other	2,223	0.001	1,245	0.001

The table details the aggregation of census information on religious affiliation to the major religious groups we work with. Column (1) gives the name of the religious group, as reported in IPUMS (Census). Column (2) reports the number of observations for the respective religious affiliation, without imposing any age restriction. Column (3) gives the population share of the respective religious group in the country. Column (4) gives the number of observations where we observe the education of the older generation in the household, needed to compile statistics of intergenerational mobility (IM) in education. Column (5) gives the religion's group population share based on individuals for whom we observe the education of the older generation. Column (6) gives the aggregation to major religious groups, we use in the analysis. There are five main religious groups: Christians, Muslims, Animists, Other, and No Religion. Column (7) gives the number of observations for each major religious group in each country, as assigned in column (6). Column (8) shows the within-country population shares of major religious groups, based on column (7). Column (9) shows the number of observations of the main religious groups for whom we observe the education of the older generation. Column (10) shows the the within-country population shares of major religious groups, based on column (9).

B.2 Cohabitation Rates

SI Figure B.1 reports cohabitation rates for Christians, Traditionalists/Animists, and Muslims across 21 African countries (panel (a)) and more than 2,000 African regions (panel (b)), pooling all (IPUMS) censuses in each country (see SI Table B.1). The three distributions are centered around the cross-country mean cohabitation rates of about 85%. The tabulation of cohabitation rates across regions reveal also cohabitation rates of about 80%, though in some regions cohabitation rates appear relatively low. [We thus repeated all estimates dropping districts with cohabitation below 80%, finding very similar results.]

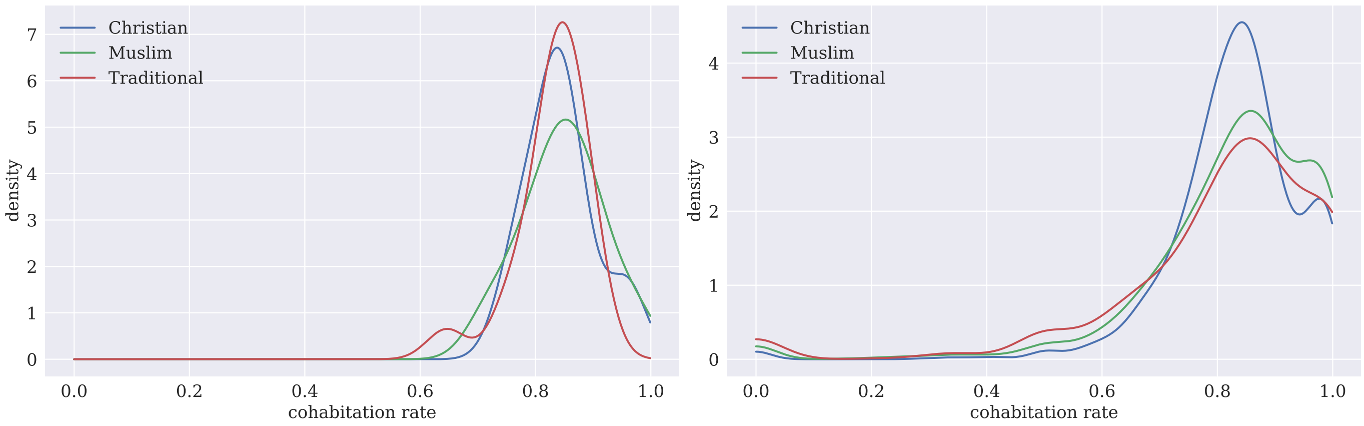

(a) Country-level, ages 14-18

(b) District-level, ages 14-18

Figure B.1: Cohabitation Rates by Major Religion across Countries and Regions

The figures plot the distribution of cohabitation rates for Christians, Muslims, and Animist young individuals, aged $14 - 18$ years, across African countries (panel (a)) and regions (panel (b)).

SI Table B.4 reports cohabitation rates for Christians, Muslims, and Traditionalists/Animists for 14-18 (columns (1)-(9)) and 14-25-year-old individuals (columns (10)-(18)) matched to the previous generation members in the household for each country-census. The cohabitation rates denote the share of 14-18-year-old children who reside with at least one older generation relative, most often, their parent(s). For each religious group and age cohort, the table gives the number of observations, the share of young residing with any relative (of any age/generation), and the share of young residing with at least one older generation relative. To get at the older generation relative to a young individual, we use census information on the relationship to the household head. When the relationship to the household head variable is inconclusive in terms of allowing us to assign the corresponding individual to an older generation we use those who are relatives of the young individual and are between 15-40 years older than him/her. This happens when this census variable is coarsely recorded. An extreme example is the Togo 2010 census, which classified 92.9% of individuals 14-18 years as cohabiting with a relative.

		Ages 14-18									Ages 14-25								
		Christian			Muslim			Traditional			Christian			Muslim			Traditional		
		(1)	(2)	(3)	(4)	(5)	(6)	(7)	(8)	(9)	(10)	(11)	(12)	(13)	(14)	(15)	(16)	(17)	(18)
iso	year	N	living with relative	living w/ old or fam mbr 15-40 yrs older	N	living with relative	living w/ old or fam mbr 15-40 yrs older	N	living with relative	living w/ old or fam mbr 15-40 yrs older	N	living with relative	living w/ old or fam mbr 15-40 yrs older	N	living with relative	living w/ old or fam mbr 15-40 yrs older	N	living with relative	living w/ old or fam mbr 15-40 yrs older
BEN	1992	19,702	97.1	78.4	9,541	98.8	76.8	13,200	98.3	78.9	43,437	95.5	64.6	21,785	97.6	67.1	30,984	97.8	64.0
BEN	2002	33,621	97	82.4	17,684	98.5	83.7	13,384	98.7	85.2	75,873	95	65.6	40,149	97.2	70.9	29,936	97.6	67.3
BEN	2013	55,726	96.9	84.3	29,922	98.7	88	14,529	98.8	89	123,268	94.9	68.3	67,351	97.6	75.7	30,314	97.9	74.0
BFA	1996	24,733	98.9	86.1	65,065	99.5	81.9	23,956	99.8	88.7	48,852	97.8	71.8	131,309	98.9	67.7	45,513	99.7	76.0
BFA	2006	38,003	98.7	82.5	93,921	99.1	80.5	21,059	99.5	84.7	80,716	97.4	67.4	203,974	98.3	64.7	41,383	99.4	70.7
BWA	2001	15,180	96.9	79.2	51	98	90.2	772	95.5	74.7	32,306	91.5	66.5	152	86.2	63.8	1,904	85.6	57.0
BWA	2011	16,672	81.1	70	135	88.1	78.5	547	65.6	50.3	38,921	82	59.9	308	85.7	64	1,468	71.2	45.2
CMR	2005	143,860	97.2	82.8	40,872	99	82.8	8,863	99.1	86.9	317,330	95.5	69	88,127	97.1	68.8	18,406	98.3	71.6
EGY	1986	40,328	99.4	96.1	683,438	99.6	96.1				92,728	98.8	85	1,518,674	99	83.5			
EGY	1996	37,503	99.6	97.2	681,371	99.7	96.8				78,015	98.9	83.9	1,393,268	99.1	83.7			
EGY	2006	40,313	98.3	96.8	745,333	98.8	96.7				101,137	96.8	82	1,877,513	97.9	80.3			
ETH	1984	204,946	97.7	76.4	79,170	98.2	80.3	15,619	96.3	75.9	407,048	95.9	58.5	166,122	96.7	60.4	35,997	95	51.5
ETH	1994	428,556	96.1	78.9	162,853	98.4	85.7	26,059	98.6	83.8	852,293	94.1	63.1	324,130	97.3	68	53,417	97.4	62.1
ETH	2007	615,020	96.7	79.4	328,378	98.3	85.4	20,616	98.3	84	1,248,643	94.2	61	647,347	97.2	67.8	41,888	97.3	62.8
GHA	2000	146,171	98.4	83.3	31,322	97.4	85.4	13,128	98.9	89.2	312,579	95.9	70.4	70,839	95.8	74.2	27,535	98.4	80.2
GHA	2010	198,240	96.1	84	47,829	97	88.1	10,997	98.6	92.1	438,179	90.6	69.3	109,803	93.9	75	23,037	97.5	81.4
GIN	1983	2,234	88.7	76.9	37,999	90.2	77	2,122	82.4	74.9	5,058	88.7	65.6	86,502	90.1	68.8	4,517	83.1	69.9
GIN	1996	5,408	99.2	83.7	60,575	99	84.8	1,297	99.3	87.5	11,457	98.2	72.5	129,690	98.4	77.4	2,862	98.7	79.7
GIN	2014	9,091	99.7	90.4	105,148	99.9	91.6	1,824	99.6	93	19,245	98.9	81	222,841	99.6	85.1	3,761	99.4	85.5
LBR	2008	33,838	97	84.1	4,387	96.2	78.6	166	93.4	81.9	75,418	95.6	69.6	10,481	94.2	63.5	376	93.1	69.7
MLI	2009	4,229	99.4	83.8	151,625	99.4	85.3	3,026	99.8	89.3	8,667	98.2	72	312,363	98.8	74.5	5,819	99.5	74.5
MOZ	2007	115,131	99	80.2	32,323	98.6	71.2	13,385	99.2	80.7	256,529	98	59.9	75,157	97.4	48.6	30,937	98.1	58.1
MUS	1990	3,021	99.6	95.1	1,499	100	97.7				7,360	99.2	82.5	3,839	99.4	88.1			
MUS	2000	3,404	99.6	96.7	1,579	99.9	98.3				8,224	99.1	85.2	3,881	99	87.9			
MUS	2011	3,295	99.4	96	1,793	99.7	98.8				7,775	98.4	87.1	4,180	95.9	88.9			
MWI	1998	94,674	97.8	73.5	13,725	97.8	68.4	3,112	97.8	72.5	206,019	96.6	53.3	30,546	96.6	47.9	7,131	96.4	49.9
MWI	2008	115,083	98.6	80.4	16,443	98.4	75.7	2,288	97.2	79.9	259,232	98	56.5	37,458	98	51.1	5,222	97.4	54.4
NGA	2010	4,492	99.2	94.5	3,339	99.8	91.3	68	100	88.2	9,249	97.3	84.8	7,047	98.7	76.1	122	98.4	78.7
RWA	1991	68,859	98.5	85.6	871	96.2	78.3	316	95.9	76.3	144,230	96.4	69.5	2,062	92.1	57.6	816	96.2	51.0
RWA	2002	104,848	97.9	81.1	1,881	96.3	73.6	40	100	72.5	208,976	96.2	65.2	4,476	92	52.6	67	100	61.2
RWA	2012	107,995	98.8	85.3	2,367	98	80.9	6	100	83.3	239,465	96.3	68.4	5,926	92.9	59.9	19	94.7	36.8
SEN	1988	3,521	99.3	83.6	67,188	99.1	85				7,842	97.8	73.1	149,075	98.7	77.8			
SEN	2002	4,934	99.3	89.1	118,906	99.7	90.6				10,712	98.1	81.8	247,816	99.4	86.2			
SEN	2013	4,988	99.4	90.9	125,953	99.4	92.4				11,367	97.8	84.9	273,400	98.9	88.5			
SLE	2004	12,570	99.3	88.3	42,557	99.4	87.5	38	100	86.8	26,925	98.5	77.9	93,634	98.6	76.5	63	98.4	84.1
TGO	1970	535	90.8	60.4	115	93	53.9	879	99.1	75.2	1,197	88.5	46.8	266	94.7	46.2	2,372	98.1	58.2
TGO	2010	32,189	97.1	76.9	10,022	98.6	83.2	13,376	98.9	84.2	74,365	94	62.7	23,236	96.7	69.3	29,223	98.1	70.9
UGA	1991	156,734	96.5	72.7	17,788	95.6	65.3	402	97	76.6	332,951	93.8	55.1	38,646	92.8	48.1	870	95.4	62.5
UGA	2002	246,958	96.7	77.5	34,663	96.7	74	3,345	95.4	83	512,288	94.2	56.5	73,848	93.8	51.7	6,513	94.2	70.2
UGA	2014	360,546	98.8	83.5	57,461	98.6	82	360	99.4	81.7	730,275	96	63.8	118,518	95.5	61	743	96.5	66.5
ZAF	1996	290,310	97.3	85.4	4,942	98.3	93.9	174	96	82.2	638,210	94.2	75.1	11,650	97.3	81.5	376	92.6	71.5
ZAF	2001	343,546	96.8	84.2	5,893	94.6	91.2	1,167	98.4	81	729,767	94.1	75.1	13,205	94.2	80.9	2,417	95	73.1
ZAF	2016	212,467	98.7	85.3	2,754	98.5	91.6	13,885	98	82.9	485,719	95.7	77.2	7,129	94.4	79.2	34,671	93.1	72.3
ZMB	2000	106,371	99.5	84.9	497	98.8	85.9	8,499	99.5	84.8	229,539	98.5	67	1,030	97.3	67.2	18,769	98.6	66.7
ZMB	2010	142,464	99.5	87.4	665	99.2	92.2	2,986	99.6	88.4	295,424	98.4	68.6	1,382	97.5	74.2	6,174	98.5	69.0
total		4,652,309	97.5	81.9	3,941,843	99	91.1	255,490	98.5	84.3	9,844,810	95.2	66.2	8,650,135	98.1	77.6	545,622	97.3	67.8

Table B.4: Cohabitation Rates by Census and Major Religion

The table reports cohabitation rates by census and major religion for all young individuals aged 14-18 (columns (1)-(9)) and 14-25 (columns (10)-(18)). For each religious group and age cohort, the table gives the number of observations, the share of young residing with any relative (of any age/generation), and the share of young residing with at least one older generation relative. To get at the older generation relative of a young individual, we use census information on the relationship to household head (typically father or mother). When the relationship to household head variable is inconclusive we use relatives of the young individual 15-40 years older than him/her.

B.3 Inter-generational Transmission of Religious Affiliation

SI Figure B.2 examines the inter-generational transmission of religion for different decade-birth cohorts, by the religious affiliation of the old generation (parents). The figure plots the likelihood that the child (boy or girl) of the household head adheres to Christianity [red solid line], Islam [blue dashed line], and Traditional religions [grey dashed line], distinguishing by the mode of the religious affiliation among the old members in the household; Christian in panel (a), Muslim in panel (b), and Animist in panel (c).

The estimates in panel (a) show that the children of Christian household heads are almost always Christian; the likelihood exceeds 97% across all cohorts. The probability that the sons or daughters of Christians convert to Islam or Traditional religions is minuscule. The patterns are similar when we look at the religious affiliation of children, whose parent(s) adhere to Islam. The likelihood that they will change to Christianity or Traditional religions is very low, as more than 96% follow Islam. The high intergenerational inertia in religious affiliation, revealed by the census tabulations, are in line with the Pew Research Center (2010) survey estimates, based on about 25,000 face-to-face interviews conducted across 19 African nations in the late 2000s. Panel (c) illustrates the conversion of Africans adhering to Traditional religions during the past decades. 30%-40% of children whose parents follow Traditional religions identify as Christians and about 5% as Muslims.

In SI Figures B.3 and B.4 we break down the transmission of religion among parents without completed primary schooling distinguishing whether their children have not finished primary school and with completed primary education. Children with completed primary education born to Traditionalists/Traditionalists parents are roughly 53% likely to report they are Christian and 46% that they follow Traditional religions. The corresponding statistics for children that did not complete primary schooling are 25% and 70%, respectively. For Animist households primary school completion increases conversion to Christianity by roughly 30 percentage points reflecting how improvements in the educational attainment of the Animist population in post-colonial Africa moved in tandem with its Christianization.

As the completion of primary education moves in tandem with religious conversion for children whose parents follow Traditional religions, we may misclassify the upward IM for Traditionalists. We thus repeated the analysis on the drivers (household/family, occupational, industry of employment, regions) of upward and downward IM restricting estimation to parents-children with the same religious affiliation. The estimated magnitudes (not shown for brevity) on the Traditional indicator that reflects the gap in IM between Christians and Traditionalists are almost identical to the baseline estimates in Figure 2. This should not come as a surprise as the number of Traditionalists in the sample is rather small.

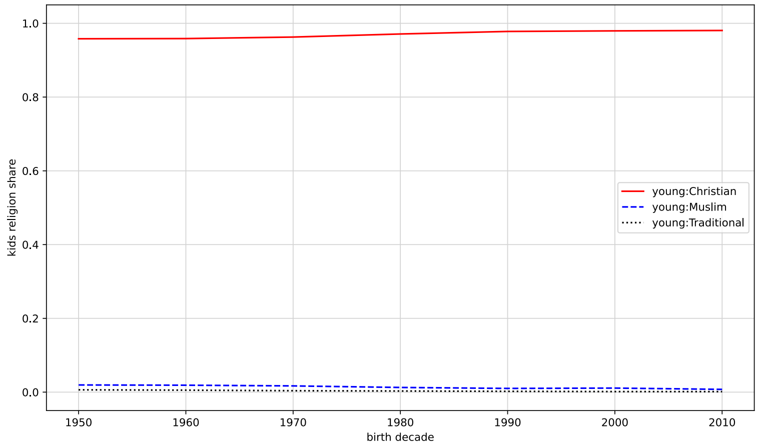

(a) Old Christians

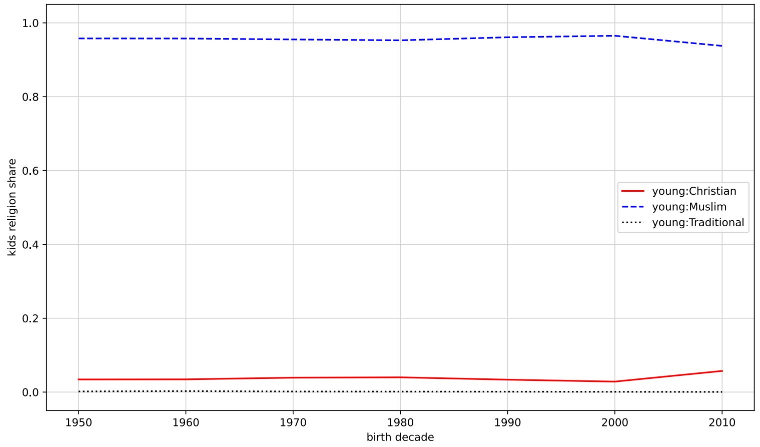

(b) Old Muslims

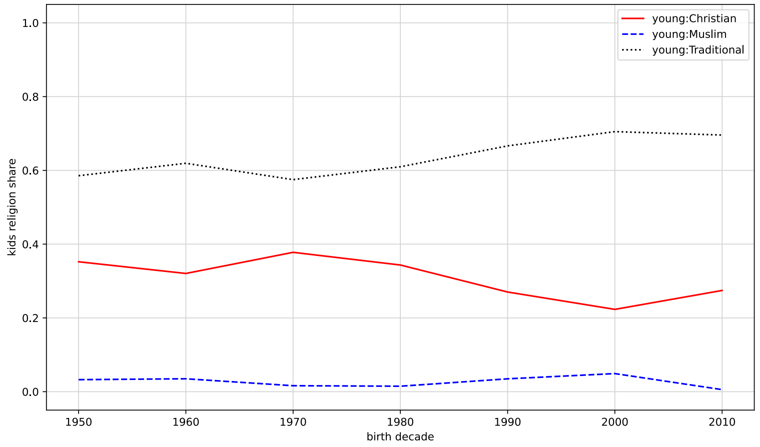

(c) Old Traditional religions

Figure B.2: Inter-generational Transmission of Religion

The figures report the likelihood by the birth cohort that cohabitating children of Christian, Muslim, and Animist parents (depicted in panels (a), (b), and (c), respectively) will adhere to Christianity (red solid line), Islam (blue dashed line), and local Traditional religions (black dotted line).

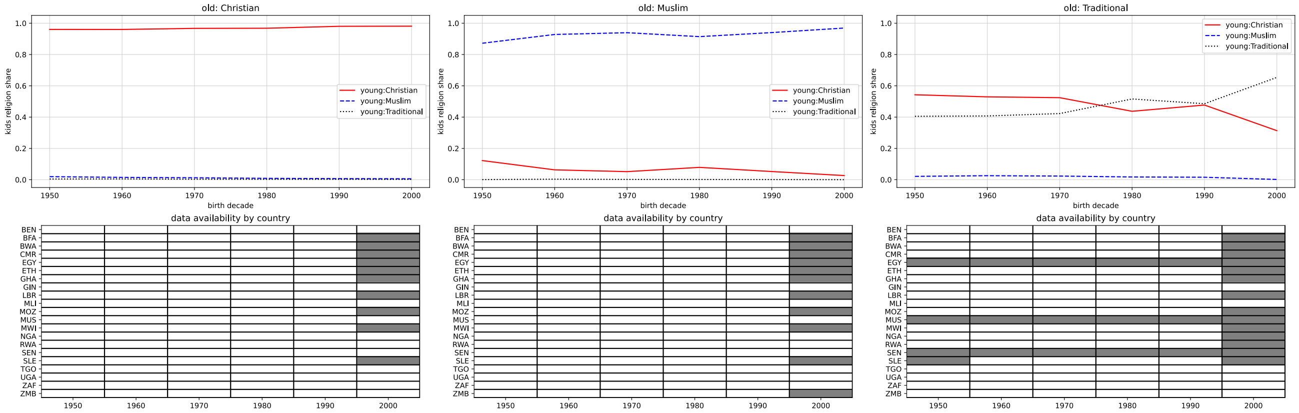

Figure B.3: Intergenerational Transmission of Religious Affiliation
Parents without Completed Primary Education - Children with Completed Primary

The three panels plot the likelihood that children cohabiting with at least one older generation member adhere to Christianity (red solid line), Islam (dashed blue line), and Traditional religions (dashed grey line) across different decade birth cohorts looking at 14-18-year-old children with completed primary educations whose parents have not completed primary education. Panel (a) tabulates the statistics for Christian household heads, panel (b) for Muslims, and panel (c) for Traditionalists. As the sample composition somewhat changes, the figures also give the countries available for the average for each birth cohort.

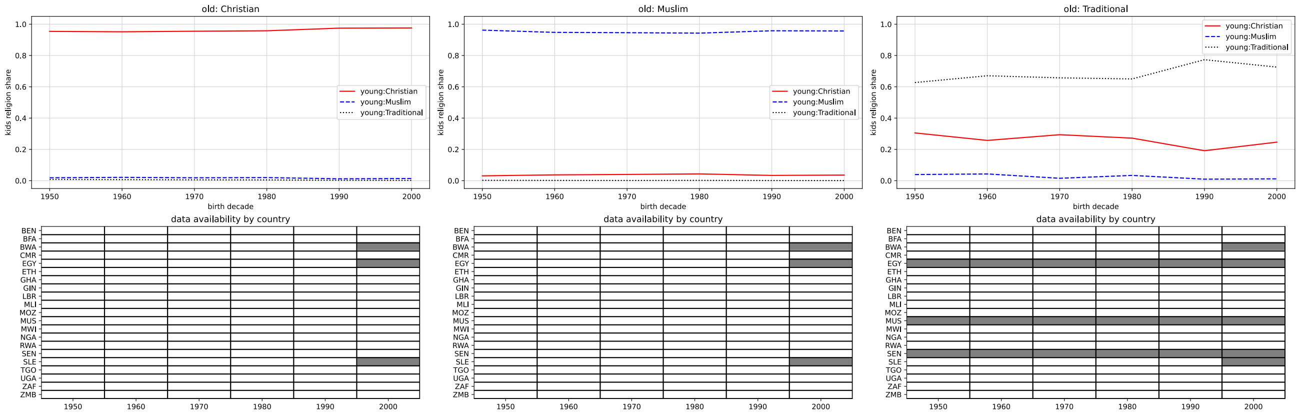

Figure B.4: Intergenerational Transmission of Religious Affiliation
Parents without Completed Primary Education - Children without Completed Primary

The three panels plot the likelihood that children cohabiting with at least one older generation member adhere to Christianity (red solid line), Islam (dashed blue line), and Traditional religions (dashed grey line) across different decade birth cohorts looking at 14-18-year-old children without completed primary educations whose parents have not completed primary education. Panel (a) tabulates the statistics for Christian household heads, panel (b) for Muslims, and panel (c) for Traditionalists. As the sample composition somewhat changes, the figures also give the countries available for the average for each birth cohort.

B.4 Religious Affiliation and Ethnicity

While ethnicity and religious affiliation intersect, there is significant within-ethnicity heterogeneity in religious adherence and considerable ethnic diversity within the three main religions.

B.4.1 Patterns

SI Figure B.5 gives the distribution of within-ethnicity religious concentration (Herfindahl-Hirschman Index) across 222 ethnic groups, as recorded by IPUMS, from 18 countries; we miss ethnicity data from Nigeria, Egypt, and Cameroon. Besides ethnicity information is not available for all censuses. For example, in Guinea ethnicity is recorded in one of the three censuses. See Appendix Table B.1 for details. For the median African ethnic group, the probability of picking randomly two individuals sharing the same religious affiliations is somewhat below 50%. The median (mean) number of religious groups per ethnicity is 5 (4.52), with a standard deviation of 0.45. There is geographic clustering in religious diversity by ethnicity. One finds many religiously diverse ethnic groups in Southern Africa, whereas in West Africa, especially Mali, Burkina Faso, and Senegal, many groups adhere mainly to Islam. The likelihood that two randomly picked individuals of the same faith will not be members of the same ethnicity is substantial for all religious groups. The median (mean) number of ethnic groups per religious group within a country is 12 (13.01) with a standard deviation of 4.15. The uncovered pattern that has not received attention in the literature reveals that religious and ethnic cleavages are quite distinct with ethnic identity transcending religion and vice versa.

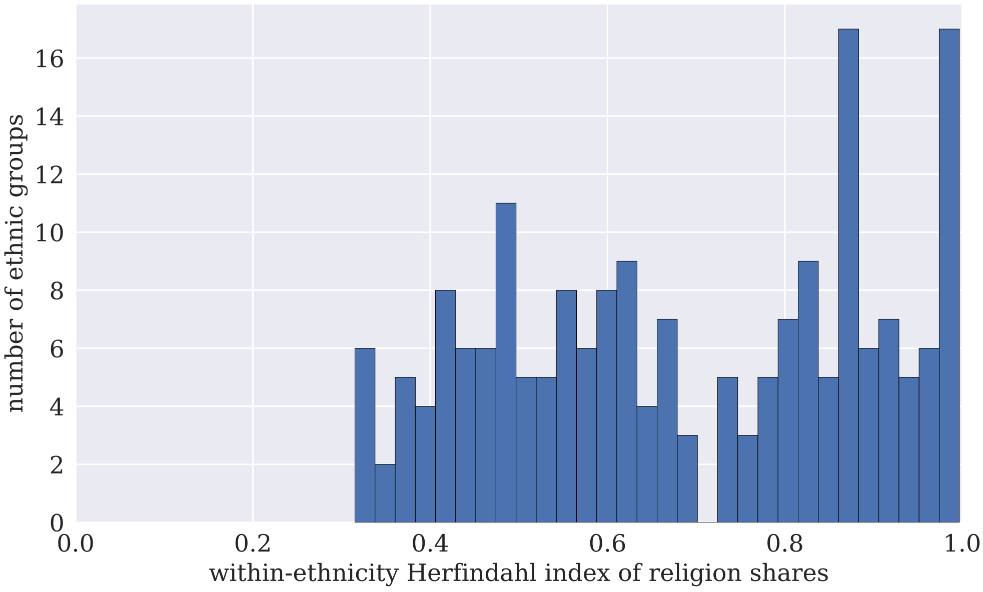

Figure B.5: Religious Concentration by Ethnicity [Herfindahl–Hirschman Index]

The figure plots a histogram of within-ethnic group Herfindahl-Hirschman indices (HHI) of the concentration of religious affiliation. Concretely, let s_{cer} denote the share of adherents of religion r among individuals belonging to ethnicity e in country c such that $\sum_r s_{cer} = 1$. Then the country-ethnicity specific HHI is defined as the sum of the squares shares, i.e. $\text{HHI}_{ce} = \sum_r s_{cer}^2$. Smaller numbers mean more heterogeneous religious affiliations within an ethnic group and an index value of 1 means a perfectly religiously homogeneous ethnic group. The figure plots this index using all available individuals with both religion and ethnicity information and does not restrict the birth cohort of individuals considered.

B.4.2 Examples

Almost exclusively Muslim ethnic groups include the Wolof and the Fula in Senegal, the Maraka-Soninke and the Peul in Mali, and the Somali in Ethiopia. Similarly, homogeneous Christian groups are the Agew in Ethiopia, the Bassa and the Grebo in Liberia, and the Acholi in Uganda.

Examples of large ethnic groups split between Christianity and Islam are the Oromo in Ethiopia, the Yoruba in Nigeria (Laitin, 1986), the Banyole in Uganda, the Emakhuwa and the Sena in Mozambique; smaller groups with a roughly equal Muslim-Christian split are the Gola in Liberia, the Limba in Sierra Leone, the Banyole in Uganda.

The Adja and the Otamari in Benin, the Gurunsi and the Bwamu in Burkina Faso, the Ouatchi in Togo, the Bobo - Dafing in Mali are roughly split between Christianity and Traditional religions.

Groups with a similar representation of Christians, Muslims, and Traditionalists are the Gurma in Ghana, the Basari in Togo, and the Goulmancema in Burkina Faso. The Mole (Dagbani) in Ghana are 45% Muslim, 35% Christian, and 15% Animist. The Otamari in Benin are 43% Animist, 35% Christian, and 6% Muslim. The San (Samo or Samogho) in Burkina Faso are 55% Muslim, 36% Christian, and 9% Animist. The Yoruba in Benin are 50% Christian, 36% Muslim, and 8.5% Animist.

B.5 Comparing African Countries in IPUMS Sample and Out of Sample

Out of the 50 African countries, 21 are in our sample. One may wonder how representative is our sample of the African continent in terms of real Gross Domestic Product (GDP) per capita, human capital, institutional quality, and proxies of state capacity. SI Table B.5 and SI Figure B.6 report the two samples. We conduct the test of means between in the sample and not in the sample countries across the four categories running ordinary least squares regressions (with heteroskedasticity robust standard errors) that associate the various economic and institutional aspects to an indicator variable that takes the value of one for the countries in our sample. Therefore, the constant term gives the mean of the outcome of the missing countries, while the estimate on the $In - Sample$ binary index gives the difference between the sample and not-in-sample countries.

(a) Countries in the sample

	**Name**
1	Benin
2	Botswana
3	Burkina Faso
4	Cameroon
5	Egypt
6	Ethiopia
7	Ghana
8	Guinea
9	Liberia
10	Malawi
11	Mali
12	Mauritius
13	Mozambique
14	Nigeria
15	Rwanda
16	Senegal
17	Sierra Leone
18	South Africa
19	Togo
20	Uganda
21	Zambia

(b) Countries not in the sample

	**Name**
1	Algeria
2	Angola
3	Burundi
4	Cape Verde
5	Central African Republic
6	Chad
7	Comoros
8	Congo
9	Democratic Republic of Congo
10	Djibouti
11	Equatorial Guinea
12	Gabon
13	Gambia
14	Guinea-Bissau
15	Ivory Coast
16	Kenya
17	Lesotho
18	Libya
19	Madagascar
20	Mauritania
21	Morocco
22	Namibia
23	Niger
24	Somalia
25	Sudan
26	Swaziland
27	Tanzania
28	Tunisia
29	Zimbabwe

Table B.5: In-Sample (21) and Not-In-Sample (29) African Countries

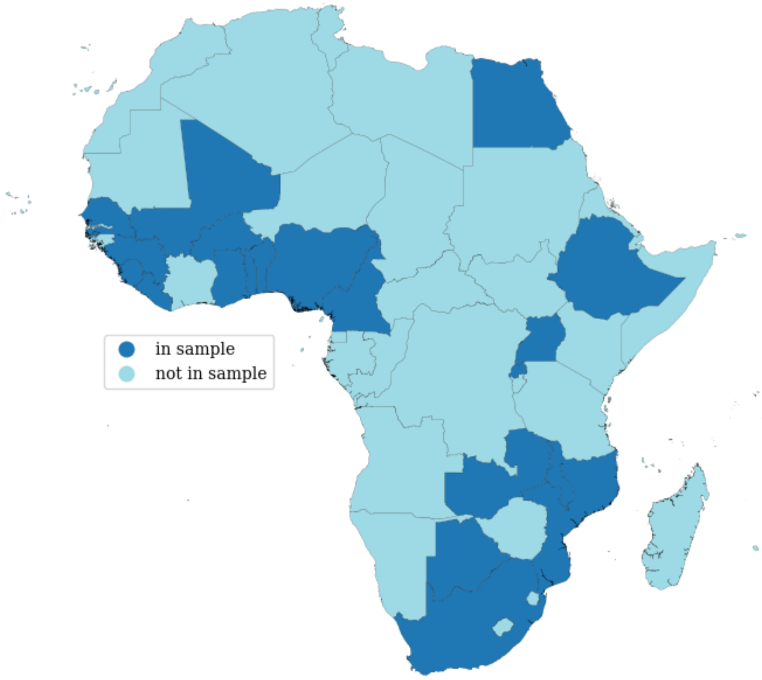

Figure B.6: In-Sample (21) and Not-In-Sample (29) African Countries

The figure plots the 21 countries in the base sample of the analysis with educational and religious adherence information from IPUMS (dark blue) and the African countries not in our sample (light blue).

Economic Development SI Table B.6 reports the comparisons of real per capita GDP in 1950, 1975, 1995, and 2000 for the sample countries and those African countries, not in the sample. The data on real GDP per capita come from Nunn and Puga (2012), who extract them from Maddison and the World Bank. The estimate on the identifier for the sample countries is small and statistically indistinguishable from zero. Our sample does not differ much from the countries we miss data from IPUMS, as it includes both relatively richer (South Africa and Botswana) and poorer African countries (Malawi and Ethiopia).

	(1) $GDP^{real,pc}_{2000}$	(2) $GDP^{real,pc}_{1950,m}$	(3) $GDP^{real,pc}_{1975,m}$	(4) $GDP^{real,pc}_{2000,m}$	(5) $GDP^{real,pc}_{1950-2000,m}$	(6) $GDP^{real,pc}_{1995}$
Sample (Difference)	-523.225	-14.473	-431.702	67.295	-168.558	-335.297
	(904.081)	(179.038)	(453.643)	(575.896)	(325.358)	(743.479)
Constant (Not in Sample)	2933.614***	916.6096***	1772.424***	1770.738***	1495.118***	2792.588***
	(596.133)	(115.569)	(292.826)	(371.739)	(210.018)	(491.765)
R^2	0.0076	0.0001	0.0193	0.0003	0.0058	0.004
N	46†	48††	48††	48††	48††	48†††

Table B.6: Test of Means: Real GDP per capita

*Data: Nunn and Puga (2012)
‡Robust standard errors are reported in parentheses. $***$, $**$, $*$, denote significance at 1%, 5%, and 10% levels, respectively, in a two-sided test of the null hypothesis of the *In-Sample* indicator equal to zero, using the t-statistic. Test statistics are not adjusted for multiple comparisons.
†No available data for 4 countries, 1 of which is in the sample of the present paper (Liberia) and 3 of which are not in the sample (Democratic Republic of Congo, Libya and Somalia).
††No available data for 2 countries, 1 of which is in the sample of the present paper (Ethiopia) and 1 which is not in the sample (Democratic Republic of Congo).
†††No available data for 2 countries, both of which are not in the sample of the present paper (Democratic Republic of Congo and Somalia).

Education SI Table B.7 reports OLS estimates that compare the percentage of the population with completed primary education in the 1950s, 1960s, 1970s, 1980s, and 1990s for the sample countries and those African countries, not in IPUMS. For the tabulation, we use the widely used Barro and Lee (2013) educational statistics. The test of means reveals no major differences in the two sets of countries, as the estimate on the *In-Sample* indicator is small and insignificant.

	(1)	(2)	(3)	(4)	(5)
	$SCPE_{1950s}$	$SCPE_{1960s}$	$SCPE_{1970s}$	$SCPE_{1980s}$	$SCPE_{1990s}$
Sample (Difference)	1.411	0.944	-1.205	-2.129	-4.193
	(1.399)	(1.394)	(1.558)	(1.962)	(2.585)
Constant (Not in Sample)	4.154***	6.129***	9.744***	13.582***	18.112***
	(0.961)	(0.958)	(1.071)	(1.348)	(1.777)
R^2	0.0290	0.0133	0.0173	0.034	0.0718
N	36†	36†	36†	36†	36†

Table B.7: Test of Means: Completed Primary Schooling

*Data: Barro and Lee (2013)
**$SCPE$ refers to the variable *share of completed primary education*.
‡Robust standard errors are reported in parentheses. $***, **, *$, denote significance at 1%, 5%, and 10% levels, respectively, in a two-sided test of the null hypothesis of the *In-Sample* indicator equal to zero, using the t-statistic. Test statistics are not adjusted for multiple comparisons.
†No available data for 14 countries, 4 of which are in the sample of the present paper (Burkina Faso, Ethiopia, Guinea and Nigeria) and 10 of which are not in the sample (Angola, Cape Verde, Chad, Comoros, Democratic Republic of Congo, Djibouti, Equatorial Guinea, Guinea-Bissau, Madagascar and Somalia).

Institutions SI Table B.8 compares sample and not-in-sample countries across proxies of institutional and governance quality aspects using data from the World Bank's Governance Matters Database. Aggregating via principal components numerous institutional quality variables provided by non-governmental organizations, think tanks, and specialized agencies, the World Bank provides institutional quality statistics reflecting control of corruption, government effectiveness, political stability and absence of violence, regulatory quality, the rule of law, and voice and accountability. The six variables range from -2.5 to +2.5, with a mean of zero, with higher values suggesting stronger institutional capacity and better governance. The indicator for the sample countries measures the difference in governance quality (using data from the 2000s) between the sample and not-in-sample countries; the intercept gives the average for the not-in-sample countries. The sample countries score somewhat higher than not-in-sample countries in all aspects of institutional quality. However, the differences are not large and statistically insignificant except for the voice and accountability index.

	(1)	(2)	(3)	(4)	(5)	(6)
	CoC	GE	$PSnAoV$	RQ	RoL	VnA
Sample (Difference)	0.226	0.203	0.363	0.278	0.330	0.444*
	(0.165)	(0.167)	(0.272)	(0.174)	(0.192)	(0.188)
Constant (Not in Sample)	-0.772***	-0.857***	-0.736***	-0.836***	-0.882***	-0.830***
	(0.106)	(0.107)	(0.175)	(0.112)	(0.123)	(0.121)
R^2	0.041	0.032	0.039	0.055	0.063	0.112
N	46[†]	46[†]	46[†]	46[†]	46[†]	46[†]

Table B.8: Test of Means: Institutional Quality

[*]Data: World Bank's Governance Matters Database
[**]Columns (1), (2), (3), (4), (5) and (6) refer to the six Worldwide Governance Indicators: *control of corruption*, *government effectiveness*, *political stability and absence of violence*, *regulatory quality*, *rule of law* and *voice and accountability, respectively*.
[‡]Robust standard errors are reported in parentheses. $***, **, *$, denote significance at 1%, 5%, and 10% levels, respectively, in a two-sided test of the null hypothesis of the *In-Sample* indicator equal to zero, using the t-statistic. Test statistics are not adjusted for multiple comparisons.
[†]No available data for 4 countries, 2 of which are in the sample of the present paper (Egypt and South Africa) and 2 of which are not in the sample (Libya and Swaziland).

State Capacity SI Table B.9 reports OLS estimates that compare various proxies of state capacity related to civil conflict and fragility for the sample countries and African countries not in the sample. Columns (1) and (2) display the estimates for the fragility of the state index (retrieved by The Fund for Peace) and the years of intrastate conflict (retrieved by the UCDP database), respectively. The FSI is an index that assesses states' vulnerability to conflict or state collapse by ranking all members of the United Nations based on the sum of scores for twelve indicators, where each indicator is scored on a scale of 0 to 10, with 0 being the lowest intensity (most stable) and 10 being the highest intensity (least stable); hence, FSI index ranges from 0 to 120 with higher values suggesting higher fragility and lower state capacity. Columns (3), (4), and (5) show the estimates for the share of income tax revenue in GDP, the share of tax revenue in GDP, and an index of anti-diversion policies, respectively; all measures are retrieved from Besley and Persson (2010). Column (6) depicts the state capacity index, retrieved by Gennaioli and Rainer (2007). There are no significant differences between sample and not-in-sample countries across all proxies of state capacity. This is because our sample includes both politically unstable, with low fiscal capacity, and recurrent conflict countries, like Ethiopia, Liberia, Mozambique, and Sierra Leone countries, besides more stable and with stronger state capacity nations, like Egypt, South Africa, and Botswana.

	(1)	(2)	(3)	(4)	(5)	(6)
	FSI	$YoIC$	$SoIT$	SoT	ADP	SC
Sample (Difference)	-2.543	-0.038	0.005	-0.059	-0.015	0.008
	(4.370)	(0.628)	(0.038)	(0.041)	(0.038)	(0.099)
Constant (Not in Sample)	90.978***	2.913***	0.149***	0.371***	0.501***	0.577***
	(2.913)	(0.402)	(0.026)	(0.029)	(0.027)	(0.063)
R^2	0.01	0.0001	0.0004	0.048	0.0045	0.0001
N	36†	39††	42‡	42‡	35‡‡	47‡‡‡

Table B.9: Test of Means: State Capacity

*Data: columns (1) and (2) use data from The Fund of Peace and the UCDP database, respectively; (3), (4), and (5) use data from Besley and Persson (2010); (6) uses data from Gennaioli and Rainer (2007).
**Columns (1), (2), (3), (4), (5), and (6) refer to the variables: *fragility of state index*, *years of intrastate conflict*, *share of income tax revenue in GDP*, *share of tax revenue in GDP*, *anti-dispersion policies index* and *state capacity index*, respectively.
$^{\clubsuit}$Robust standard errors are reported in parentheses. $***$, $**$, $*$, denote significance at 1%, 5%, and 10% levels, respectively, in a two-sided test of the null hypothesis of the *In-Sample* indicator equal to zero, using the t-statistic. Test statistics are not adjusted for multiple comparisons.
†No available data for 14 countries, 5 of which are in the sample of the present paper (Ghana, Mali, Mauritius, Senegal, and South Africa) and 9 of which are not in the sample (Cape Verde, Comoros, Congo, Djibouti, Lesotho, Madagascar, Namibia, Niger, and Swaziland).
††No available data for 11 countries, 5 of which are in the sample of the present paper (Benin, Botswana, Malawi, Mauritius, and Zambia) and 6 of which are not in the sample (Cape Verde, Comoros, Equatorial Guinea, Namibia, Swaziland, and Tanzania).
‡No available data for 8 countries, 1 of which is in the sample of the present paper (Liberia) and 7 of which are not in the sample (Angola, Cape Verde, Democratic Republic of Congo, Guinea-Bissau, Libya, Somalia, and Sudan).
‡‡No available data for 15 countries, 4 of which are in the sample of the present paper (Benin, Mauritius, Rwanda, and Sierra Leone) and 11 of which are not in the sample (Burundi, Cape Verde, Central African Republic, Chad, Comoros, Democratic Republic of Congo, Djibouti, Equatorial Guinea, Lesotho, Mauritania, and Swaziland).
‡‡‡No available data for 3 countries, 2 of which are in the sample of the present paper (Mauritius and South Africa) and 1 of which is not in the sample (Cape Verde).

C Religious Intergenerational Mobility across Countries and Regions

This Supplementary Information (SI) Section complements the analysis in Section of the main paper, where we present the newly-constructed measures of absolute upward IM and absolute downward IM gaps across African countries and regions between Christians, Muslims, and Traditionalists/Traditionalists. First, we provide additional country measures of educational IM. Second, we map educational mobility for Christians, Muslims, and Traditionalists/Animists across more than 2,000 African regions. Third, we provide summary statistics and descriptives for the regional IM measures.

C.1 Additional Country-Level Religious Educational IM Measures

SI Table C.1 reports absolute upward and absolute downward IM for Christians, Muslims, and Animists, estimated across 14-25 years old children (young generation) cohabiting with at least one older generation member in the household. The table, therefore, mirrors Table 1 that reports similar statistics for the 14-18 age bracket. The benefit of looking at 14-25-year-old young individuals, as compared to 14-18, is that the sample almost doubles (from about 7.2 to 13.02 million observations), although cohabitation rates fall to about 70%. The cross-country patterns are similar as the correlation of upward and downward IM across the two age brackets exceeds 0.95.

SI Table C.2 reports absolute upward IM (panel (a)) and absolute downward IM (panel (b)) for Christians, Muslims, and Traditionalists across countries, distinguishing by the gender of the young looking across 14-18 year boys and girls. Columns (1)-(3) plot the IM measures for girls and column (4) reports

	upward IM					downward IM				
	(1)	(2)	(3)	(4)	(5)	(6)	(7)	(8)	(9)	(10)
Cameroon	0.558	0.699	0.347	0.363		0.078	0.058	0.27	0.297	
Nigeria	0.625	0.808	0.463	0.233		0.083	0.066	0.141	0.0	
Senegal	0.244	0.534	0.235			0.264	0.157	0.274		
Benin	0.291	0.412	0.204	0.201		0.293	0.265	0.332	0.518	
Ghana	0.551	0.655	0.453	0.244		0.142	0.125	0.243	0.448	
Burkina Faso	0.197	0.334	0.184	0.069		0.241	0.193	0.291	0.565	
Togo	0.54	0.661	0.538	0.395		0.18	0.153	0.212	0.352	
Mali	0.276	0.397	0.276	0.187		0.231	0.209	0.231	0.515	
Mozambique	0.31	0.345	0.232	0.398		0.253	0.229	0.331	0.203	
Botswana	0.825	0.848	0.741	0.729		0.061	0.057	0.012	0.069	
Sierra Leone	0.268	0.344	0.251	0.128		0.322	0.238	0.384	0.429	
Malawi	0.256	0.275	0.19	0.161		0.35	0.341	0.439	0.463	
Ethiopia	0.165	0.199	0.114	0.028		0.295	0.272	0.443	0.659	
Guinea	0.195	0.264	0.192	0.167		0.413	0.449	0.398	0.682	
Egypt	0.708	0.713	0.707			0.04	0.034	0.04		
Zambia	0.488	0.497	0.51	0.474		0.194	0.19	0.173	0.227	
Liberia	0.222	0.218	0.266	0.103		0.538	0.537	0.544	0.632	
Uganda	0.434	0.44	0.511	0.02		0.254	0.257	0.235	0.548	
Mauritius	0.912	0.875	0.948			0.017	0.027	0.016		
Rwanda	0.259	0.262	0.37	0.139		0.421	0.421	0.34		
South Africa	0.753	0.762	0.881	0.775		0.091	0.091	0.036	0.159	

Table C.1: Country-group-level estimates of IM, ages 14-25

This table reports upward and downward IM measures for the 1980s cohort (the cohort with the broadest coverage) for individuals aged 14-25 by country and major religious group. Because of the timing of censuses, we have to use the 1990s cohort for Liberia, Mali, Nigeria, and Togo as for those countries we don't have 14-25 year olds born in the 1980s. Columns (1) - (4) show the estimates for country-cohort-level upward IM, columns (6) - (9) those for downward IM. Columns (1) and (6) give the country-level estimates across all groups, columns (2) - (4) and (7) - (9) give estimates for Christians, Muslims, and Traditionalists, respectively. Columns (5) and (10) show differences between Christians and Muslims. Red bars indicate a negative difference, green bars a positive difference.

the difference in IM between Christian and Muslim girls. Columns (5)-(7) plot IM measures for boys and column (8) reports the differences in IM between Christian and Muslim boys. In column (9) we report the difference-in-difference in IM between Muslim and Christian boys and girls. Red (green) bars indicate that the IM gap between Muslims and Christians is larger (smaller) for boys than girls.

Panel A: Upward IM

	female				male				diff-in-diff
	(1)	(2)	(3)	(4)	(5)	(6)	(7)	(8)	(9)
country	Overall	Christian	Muslim	$\Delta(c-m)$	Overall	Christian	Muslim	$\Delta(c-m)$	$\Delta(f-m)\Delta(c-m)$
Nigeria	0.608	0.801	0.426		0.616	0.773	0.489		
Cameroon	0.553	0.696	0.35		0.656	0.783	0.485		
Senegal	0.205	0.471	0.197		0.285	0.582	0.276		
Ghana	0.526	0.625	0.421		0.585	0.682	0.51		
Botswana	0.846	0.865	0.667		0.745	0.768	0.333		
Benin	0.213	0.316	0.155		0.37	0.504	0.264		
Burkina Faso	0.165	0.299	0.157		0.214	0.362	0.203		
Mozambique	0.073	0.29	0.151		0.096	0.354	0.249		
Mali	0.231	0.351	0.231		0.311	0.433	0.31		
Togo	0.465	0.585	0.466		0.577	0.695	0.59		
Sierra Leone	0.213	0.283	0.197		0.309	0.354	0.3		
Ethiopia	0.106	0.128	0.071		0.124	0.146	0.092		
Malawi	0.131	0.141	0.092		0.134	0.145	0.1		
Guinea	0.11	0.143	0.113		0.242	0.31	0.239		
Zambia	0.427	0.433	0.43		0.448	0.457	0.515		
Egypt	0.637	0.629	0.638		0.706	0.724	0.705		
Liberia	0.215	0.212	0.243		0.229	0.223	0.29		
Mauritius	0.939	0.918	0.979		0.895	0.842	0.939		
South Africa	0.778	0.785	0.888		0.687	0.691	0.861		
Uganda	0.389	0.389	0.506		0.41	0.417	0.467		
Rwanda	0.175	0.175	0.311		0.187	0.191	0.236		

Panel B: Downward IM

	female				male				diff-in-diff
	(1)	(2)	(3)	(4)	(5)	(6)	(7)	(8)	(9)
country	Overall	Christian	Muslim	$\Delta(c-m)$	Overall	Christian	Muslim	$\Delta(c-m)$	$\Delta(f-m)\Delta(c-m)$
Cameroon	0.072	0.044	0.237		0.057	0.039	0.154		
Ethiopia	0.362	0.341	0.507		0.323	0.301	0.455		
Sierra Leone	0.371	0.287	0.437		0.293	0.222	0.338		
Senegal	0.302	0.183	0.314		0.226	0.141	0.234		
Mozambique	0.58	0.238	0.365		0.577	0.212	0.272		
Ghana	0.19	0.173	0.3		0.156	0.14	0.228		
Malawi	0.497	0.489	0.596		0.527	0.516	0.635		
Togo	0.229	0.199	0.294		0.15	0.128	0.141		
Nigeria	0.096	0.079	0.168		0.086	0.076	0.155		
Burkina Faso	0.302	0.263	0.34		0.157	0.118	0.193		
Benin	0.383	0.364	0.395		0.188	0.168	0.212		
Liberia	0.548	0.546	0.565		0.529	0.528	0.525		
Zambia	0.251	0.25	0.257		0.255	0.253	0.194		
Egypt	0.055	0.052	0.055		0.049	0.045	0.05		
Mali	0.295	0.291	0.292		0.18	0.139	0.18		
Mauritius	0.015	0.019	0.014		0.022	0.037	0.015		
Botswana	0.066	0.065	0.056		0.109	0.106	0.0		
South Africa	0.077	0.077	0.033		0.13	0.135	0.048		
Uganda	0.276	0.283	0.228		0.305	0.307	0.288		
Guinea	0.53	0.594	0.507		0.346	0.394	0.328		
Rwanda	0.534	0.534	0.446		0.552	0.548	0.534		

Table C.2: Country-group-level estimates of IM for Christians and Muslims by gender

The table reports educational Intergenerational Mobility (IM) statistics for the 1980s cohort (the cohort with the broadest coverage) for individuals aged 14-18 by country and major religious group (Christians and Muslims) distinguishing by children's gender. Panel A gives estimates for upward IM and panel B for downward IM. Because of differences in the timing of censuses, reported in IPUMS, the estimates for Liberia, Mali, Nigeria, and Togo correspond to the 1990s cohort. Columns (1) - (4) show the estimates for females, columns (5) - (8) for males. Columns (1) and (5) give the country-level estimates across all religious groups, columns (2) - (3) and (6) - (7) give estimates for Christians and Muslims, respectively. Columns (4) and (8) show differences between Christians and Muslims for females and males respectively. Red bars indicate a negative difference, green bars a positive difference. Column (9) shows the difference-in-difference, i.e., the female difference between Christians and Muslims minus the analogous male difference in educational IM (column 4 minus column 8).

C.2 Regional IM across Religious Affiliation

SI Figure C.1 plots educational upward IM for Christians (panel (a)), Muslims (panel (b)), and Traditionalists (panel (c)) across 2,286 districts in 21 countries. [The maps in Figure 1 plotting the religious IM differences between Christian-Muslims and Christian-Traditionalists are based on these regional statis-

tics.] The three panels, therefore, provide a mapping of the African land of opportunity for young children adhering to different faiths. Upward IM is higher in Southern Africa (South Africa and Botswana). Upward IM for Muslims is lower than for Christians across Ethiopia and West Africa. Muslims' IM is high in Rwanda and Uganda, and some parts of Zambia. Africans adhering to Traditional religions fare relatively well in Southern Africa (Botswana, Zambia) and Southern Ghana.

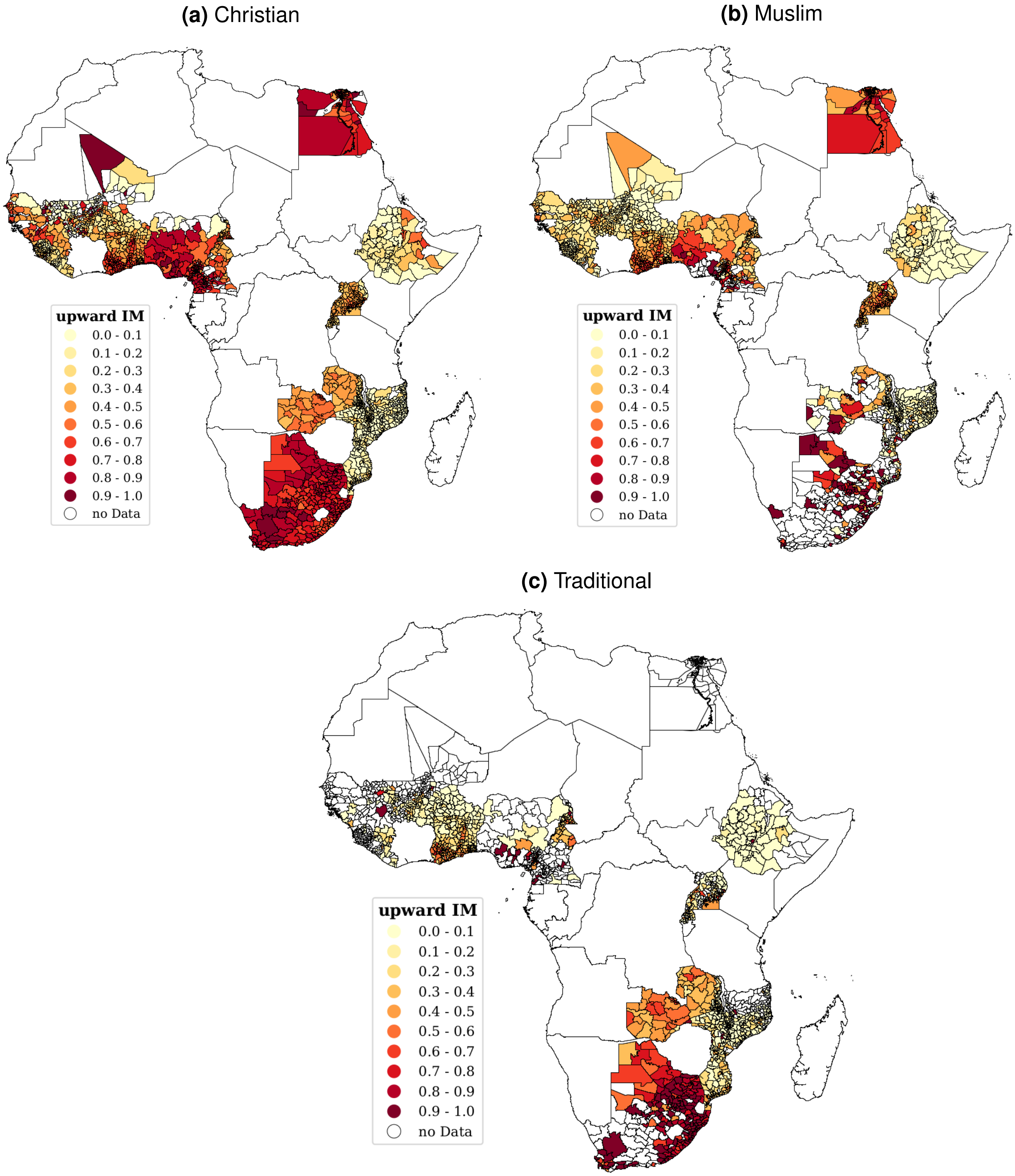

Figure C.1: Religious Upward IM across African Regions

The figures plot upward IM for Christians (panel (a)), Muslims (panel (b)), and Africans adhering to traditional local religions (panel (c)) across districts (typically admin-2 and admin-3 regions). The maps are in a single, common-across-all-religious-groups scale to facilitate comparisons. Upward IM is grouped into ten bins, with red(der) color indicating higher educational mobility and yellow(er) color indicating lower IM. The estimates of intergenerational mobility in educational attainment are based on children 14-18 years old, cohabiting with at least one older generation member, typically a parent.

SI Table C.3 gives information on the district size (population and land area) for each of the 21 countries in the sample.

Table C.3: Summary Stats: Population and Land Area Size of Districts

	country	census year	(1) $N_{districts}$	(2) Pop_{mean}	(3) Pop_{median}	(4) Pop_{std}	(5) $Area_{mean}$	(6) $Area_{median}$	(7) $Area_{std}$
1	Benin	2013	77	131,128.96	110,540.00	102,967.91	1,497.34	528.32	1,707.52
2	Botswana	2011	21	96,072.38	71,530.00	72,861.99	27,531.44	23,913.64	29,285.13
3	Burkina Faso	2006	45	279,082.22	222,890.00	267,698.53	6,064.75	5,364.41	3,245.88
4	Cameroon	2005	204	84,305.83	48,360.00	192,256.63	1,906.24	744.70	2,791.24
5	Egypt	2006	235	309,890.81	264,160.00	290,551.82	4,157.36	154.19	29,683.78
6	Ethiopia	2007	89	815,759.67	462,853.49	787,542.39	11,759.40	8,182.72	13,744.57
7	Ghana	2010	110	224,208.09	166,770.00	277,526.76	2,173.57	1,383.67	2,404.33
8	Guinea	2014	33	318,459.39	278,290.00	275,607.27	7,421.99	6,010.84	4,564.31
9	Liberia	2008	42	82,050.00	44,680.00	151,506.37	2,283.76	1,525.11	2,337.57
10	Malawi	2008	183	73,332.08	55,280.00	78,338.49	632.12	343.95	1,705.34
11	Mali	2009	241	60,094.36	40,950.00	121,665.72	5,148.94	1,676.01	20,864.22
12	Mauritius	2011	39	31,586.92	26,350.00	16,086.21			
13	Mozambique	2007	337	60,029.53	39,020.00	84,161.29	2,154.72	1,322.24	2,787.70
14	Nigeria	2010	37	4,302,545.86	3,875,619.00	1,948,722.79	23,922.16	20,649.40	17,863.38
15	Rwanda	2012	30	346,123.00	333,280.00	52,201.38	843.06	678.61	435.19
16	Senegal	2013	27	485,050.29	385,976.76	331,655.82	7,275.37	2,946.98	11,660.28
17	Sierra Leone	2004	100	49,429.80	36,035.00	76,242.31	677.38	544.80	557.07
18	Togo	2010	37	170,059.29	125,092.39	165,455.91	1,536.24	1,206.95	1,051.43
19	Uganda	2014	137	255,952.26	207,810.00	203,271.32	1,761.93	1,061.60	2,981.69
20	South Africa	2016	206	270,164.75	128,012.57	600,948.16	3,166.26	1,114.96	6,460.55
21	Zambia	2010	55	240,358.73	171,760.00	259,697.21	13,656.22	13,244.24	10,833.17
Total			2,285						

This table reports descriptive statistics regarding the population and area size of districts for each country. Column (1) shows the total number of districts. Columns (2), (3), and (4) display the mean, median and standard deviation population size of districts in each country for the 1980s cohort, respectively. Columns (5), (6), and (7) exhibit the mean, median, and standard deviation of the land area size in square kilometers across districts in each country, respectively. There is no shapefile for Mauritius. We focus on the districts that appear in the latest census of each country. One district drops for Ethiopia since it is not included in the most recent (2007) census.

C.3 Summary Statistics. Regional Educational IM Series

SI Tables C.4 and C.5 report summary statistics of absolute upward and downward IM across African regions for Christians, Muslims, and Traditionalists. As the year of the Census(es) differ across countries, we do not have information for all regions across all main birth cohorts. SI Table C.6 reports the correlation matrix of upward and downward IM across African regions for Christians, Muslims, and Traditionalists.

(a) All districts

religion	direction	N	mean	min	p10	p50	p90	max
Christian	up	2,188	0.454	0.0	0.081	0.442	0.839	1.0
Muslim	up	1,998	0.386	0.0	0.04	0.333	0.798	1.0
Traditional	up	1,333	0.313	0.0	0.0	0.207	0.903	1.0
Christian	down	2,041	0.306	0.0	0.034	0.25	0.667	1.0
Muslim	down	1,869	0.344	0.0	0.0	0.291	0.786	1.0
Traditional	down	899	0.408	0.0	0.0	0.353	1.0	1.0

The table gives summary statistics (number of observations, mean, median, minimum, maximum, p10 and p90) for the regional measures of upward and downward IM for Christians, Muslims, and Animists, pooling across all birth-cohorts. The educational IM statistics are estimated across 14-18 years old young individuals cohabiting with older generation relatives across African districts in 21 countries.

(b) Districts with more than 10 observations per Religion

religion	direction	N	mean	min	p10	p50	p90	max
Christian	up	1,998	0.464	0.0	0.1	0.453	0.833	1.0
Muslim	up	1,618	0.366	0.0	0.069	0.313	0.723	1.0
Traditional	up	836	0.317	0.0	0.04	0.231	0.826	1.0
Christian	down	1,944	0.308	0.0	0.042	0.25	0.643	1.0
Muslim	down	1,551	0.367	0.0	0.044	0.318	0.765	1.0
Traditional	down	671	0.451	0.0	0.0	0.419	1.0	1.0

The table gives summary statistics (number of observations, mean, median, minimum, maximum, p10 and p90) for the regional measures of upward and downward IM for Christians, Muslims, and Animists, pooling across all birth-cohorts. The educational IM statistics are estimated across 14-18 years old young individuals cohabiting with older generation relatives across African districts, with more than 10 observations, in 21 countries.

Table C.4: Summary Statistics, District-level IM

(a) 1950s

religion	direction	N	mean	min	p10	p50	p90	max
Christian	up	256	0.498	0.0	0.088	0.567	0.823	1.0
Muslim	up	273	0.399	0.0	0.049	0.413	0.73	0.928
Traditional	up	71	0.072	0.0	0.0	0.008	0.185	1.0
Christian	down	233	0.267	0.0	0.0	0.182	0.667	1.0
Muslim	down	252	0.329	0.0	0.077	0.261	0.667	1.0
Traditional	down	19	0.654	0.0	0.0	1.0	1.0	1.0

(b) 1960s

religion	direction	N	mean	min	p10	p50	p90	max
Christian	up	864	0.524	0.0	0.147	0.516	0.877	1.0
Muslim	up	719	0.414	0.0	0.05	0.396	0.85	1.0
Traditional	up	273	0.13	0.0	0.0	0.042	0.283	1.0
Christian	down	839	0.236	0.0	0.0	0.158	0.572	1.0
Muslim	down	685	0.298	0.0	0.0	0.237	0.741	1.0
Traditional	down	98	0.607	0.0	0.0	0.708	1.0	1.0

(c) 1970s

religion	direction	N	mean	min	p10	p50	p90	max
Christian	up	1,879	0.457	0.0	0.09	0.424	0.864	1.0
Muslim	up	1,559	0.389	0.0	0.0	0.333	0.947	1.0
Traditional	up	986	0.286	0.0	0.0	0.158	0.826	1.0
Christian	down	1,742	0.279	0.0	0.0	0.2	0.647	1.0
Muslim	down	1,355	0.35	0.0	0.0	0.286	0.906	1.0
Traditional	down	580	0.436	0.0	0.0	0.365	1.0	1.0

(d) 1980s

religion	direction	N	mean	min	p10	p50	p90	max
Christian	up	2,185	0.463	0.0	0.079	0.446	0.87	1.0
Muslim	up	2,000	0.388	0.0	0.0	0.312	0.9	1.0
Traditional	up	1,273	0.324	0.0	0.0	0.216	1.0	1.0
Christian	down	2,035	0.295	0.0	0.0	0.209	0.693	1.0
Muslim	down	1,856	0.346	0.0	0.0	0.286	1.0	1.0
Traditional	down	864	0.41	0.0	0.0	0.333	1.0	1.0

(e) 1990s

religion	direction	N	mean	min	p10	p50	p90	max
Christian	up	2,157	0.496	0.0	0.056	0.5	0.976	1.0
Muslim	up	1,902	0.414	0.0	0.024	0.333	0.901	1.0
Traditional	up	1,162	0.343	0.0	0.0	0.235	1.0	1.0
Christian	down	1,991	0.297	0.0	0.0	0.221	0.692	1.0
Muslim	down	1,745	0.331	0.0	0.0	0.25	0.833	1.0
Traditional	down	745	0.368	0.0	0.0	0.3	1.0	1.0

Table C.5: Summary Statistics, District-level IM, by birth cohorts

The table gives summary statistics (number of observations, mean, median, minimum, maximum, p10 and p90) for the regional measures of upward and downward IM for Christians, Muslims, and Animists for the 1950s, 1960s, 1970s, 1980s and 1990s birth cohorts, respectively. The educational IM statistics are estimated across 14-18 years old young individuals cohabiting with older generation relatives across African districts in 21 countries.

		upward			downward		
		Christian	Muslim	Traditional	Christian	Muslim	Traditional
upward	Christian	1.0					
	Muslim	0.732	1.0				
	Traditional	0.724	0.603	1.0			
downward	Christian	-0.76	-0.606	-0.579	1.0		
	Muslim	-0.625	-0.633	-0.444	0.615	1.0	
	Traditional	-0.6	-0.431	-0.581	0.525	0.388	1.0

Table C.6: Correlation matrix, district-level IM, ages 14-18, all birth decades

The table reports the correlation between upward IM and downward IM for Christians, Muslims, and Animists, estimated among 14-18 old young individuals cohabiting with older generation relatives across African districts in 21 countries.

D Drivers of Religious Intergenerational Mobility Gaps

This Supplementary Information (SI) Section complements the analysis that explores the role of family/household features, profession/industry of employment, and regional constants in explaining the IM gap between Christians on the one hand, and Muslims or Africans adhering to local religions, on the other (Section in the paper). First, we report estimates distinguishing by children's gender (Section D.1). Second, we report sensitivity checks (Section D.2.2). Third, we compare individuals adhering to different religious groups along family/household features and the occupation and employment sector of the household head (Section D.3). Fourth, we go over our method to match regions on the similarity of the education of the old generation across religious affiliation (Section D.4).

D.1 Gender Differences

SI Figure D.1 plots the evolution of Christian-Muslim (blue bars) and Christian - Animist gaps (green bars) in upward IM and downward IM separately for boys and girls, as we augment the baseline specification with household/family features (household size, relationship to household head, multi-generational presence) [bar 2]; the previous generation's occupation, the industry of employment, and household's rural/urban status [bar 3]; the share with completed primary education for the old generation of the same religious group in a district [bar 4]; and district fixed effects interacted with rural/urban residence [bar 5]. To further condition on unobserved heterogeneity, we also restrict estimation of [bar 5] to half of a country's districts where inter-religious educational differences among the old are the smallest [bar 6]. The figure is, therefore, similar to the main Figure 2, where we did not distinguish between children's gender. The upward IM regressions are run in a sample of 2,637,859 (young boys) and 2,352,071(young girls) individuals who cohabit with older generation members without completing primary education. The downward IM regressions are run in a sample of 947,437 (young boys) and 972,272 (young girls) individuals who cohabit with older generation members with completed primary education or higher.

The following patterns emerge. First, household and family features play a small role in explaining the considerable differences in upward and downward IM for *both* boys and girls. Household and family traits are somewhat more important in explaining Christian-Muslim and Christian-Animist differences in downward IM for girls. Second, the profession and industry of employment of the household head play a

considerable role in explaining the Christian-Animist gaps for both boys and girls. Economic features are way more important in explaining downward IM. Third, conditioning on the share of the old with completed primary education for each religious group in the district eliminates the Christian-Animist difference in downward IM for both boys and girls and halves the Christian-Animist gap in upward IM. Differences between Christian and Muslim boys and girls are halved when we condition on the literacy rate of each religious group's old generation in the district. Fourth, adding region interacted with urban/rural status constants bring the Christian-Muslim upward IM gap for boys to 6 percentage points (pps) and for girls to $4pps$. The within-district downward IM difference between Christian and Muslim boys is small, about $1.5pps$, much smaller than for girls, $4pps$. The within-district Christian-Animist upward IM gap for boys is $9pps$ and $7pps$ for girls. Regional features eliminate the Christian-Animist difference in downward IM for both boys and girls. Fifth, zooming into regions with small inter-religious differences in literacy rate among the old does not much change the Christian-Muslim and the Christian-Animist gap in upward IM; the gap is about $5pps$ for boys and $3pps$ for girls. Turning to downward IM, the Christian-Muslim gap is about $2 - 3pp$ for girls and boys. The tabulations suggest a difference between Christian and Animist boys of $2pps$; however, if anything, young Animist girls born to parents with completed primary education have a lower likelihood of not completing primary school than Christian girls. Yet, the sample shrinks, as the number of parents adhering to Traditional religions with completed primary education is small to start with, and in this specification, we restrict estimation to half of the districts.

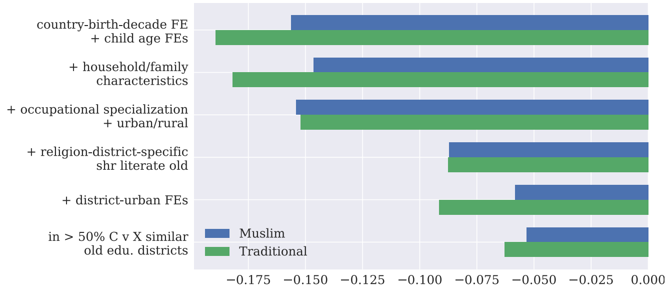

(a) Boys, upward IM

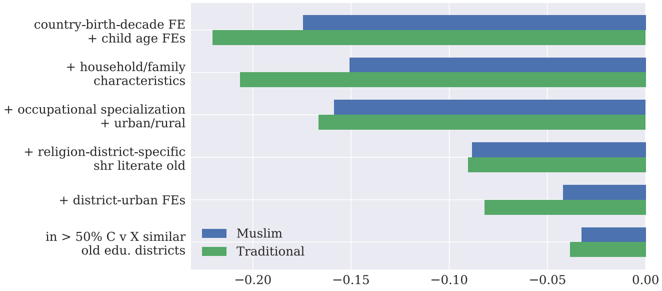

(b) Girls, upward IM

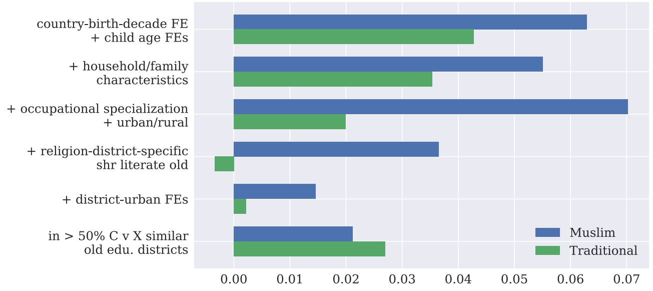

(c) Boys, downward IM

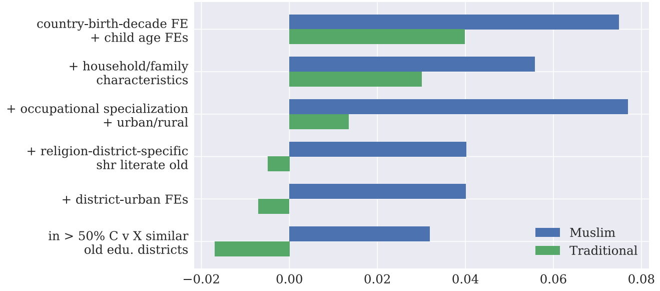

(d) Girls, downward IM

Figure D.1: Individual-level Religious IM Gap Drivers, by Gender

The figure shows how the Christian-Muslim (blue bars) and the Christian-Animist (green bars) differences in upward IM (panels (a) and (b)) and downward IM (panels (c) and (d)) change as we add controls for the household structure (model (2)), parental occupational specialization, the industry of employment, and rural-urban residence (model (3)), the share with completed primary education of the old generation of the same religious group in the district (model (4)), and district x rural/urban fixed effects (model (5)) for each country. The last permutation (model (6)) restricts estimation in half of each country's districts, where differences in completed primary education of the old generation between Christians and Muslims and Christians and Animists are the smallest. The bars on the top (model (1)) reflect the baseline inter-religious differences in IM, conditioning on birth-cohort fixed effects, and age constants. Panels (a) and (b) look at $14-18$ years old males. Panels (b) and (d) look at $14-18$ years old females. The sample consists of Muslim, Animist, and Christian young individuals, matched to the previous generation in the household. The figure gives weighted linear probability model (OLS) estimates using countries' 1980 population for the weighting to account for differential IPUMS sampling/coverage across countries.

D.2 Sensitivity Checks

D.2.1 Biological Children

SI Figure D.2 reports how the religious IM gap changes as we progressively condition on household/family features (household size, relationship to household head, multi-generational presence), the previous generation's occupation, the industry of employment, and household's rural/urban status, and district fixed effects interacted with rural/urban. Compared to the baseline Figure 2 in the main paper, we restrict estimation to 14-18 year-old individuals (young generation) matched to their (biological) parents only.

The results echo the baseline estimates. Household features explain about 10% of the considerable differences in IM between Christians and Animists (green bar) and between Christians and Muslims (blue bar). Occupation/profession and industry of employment do explain partly the Christian-Animist gap in downward and upward IM. Although professional and employment patterns between Christians and Muslims are broadly similar, augmenting the specification with indicators for profession, industry, and rural/urban residence if anything increases the Christian-Muslim gap, particularly regarding downward mobility. The share of the old generation's completed primary schooling and regional features explain

roughly two-thirds of the differences in both upward and downward mobility between Christians and Muslims.

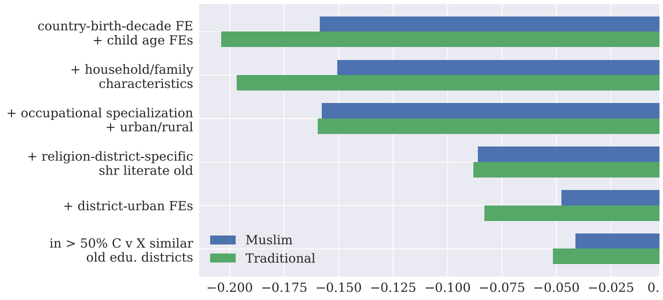

(a) Upward IM

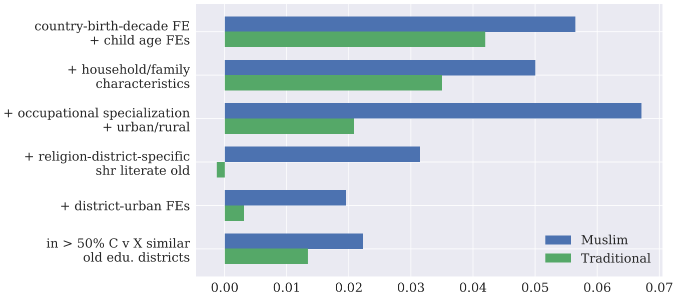

(b) Downward IM

Figure D.2: Individual-Level Religion IM Gap, Children Matched to Biological Parents

The figure shows how the Christian-Muslim (blue bars) and the Christian-Animist differences in upward IM (panel (a)) and downward IM (panel (b)) change as we add controls for the household structure (model (2)), parental occupational specialization, the industry of employment, and rural-urban residence (model (3)), the share with completed primary education of the old generation of the same religious group in the district (model (4)), and district x rural/urban fixed effects (model (5)) for each country. The last permutation (model (6)) restricts estimation to half of each country's districts, where differences in completed primary education of the old generation between Christians and Muslims and Christians and Animists are the smallest. The bars on the top (model (1)) reflect the baseline inter-religious differences in IM, conditioning on birth-cohort fixed effects, and age-specific constants. The sample consists of Muslim, Animist, and Christian young individuals (14-18 years), matched to their biological parents (mothers, fathers, or both). The figure gives weighted linear probability model (OLS) estimates using countries' 1980 population for the weighting to account for differential IPUMS sampling/coverage across countries.

D.2.2 Ethnicity

SI Figure D.3 reports the religious IM gaps in upward (panels (a)-(b)) and downward IM (panels (c)-(d)) across individuals for whom IPUMS records ethnic affiliation. The countries with such information (number of ethnicities) are: Benin (9), Botswana (9), Burkina Faso (13), Ethiopia (16), Ghana (9), Liberia (16), Malawi (10), Mali (13), Mauritius (6), Mozambique (18), Senegal (9), Sierra Leone (12), South Africa (11), Togo (20), Uganda (19), and Zambia (11), see also SI Table B.1 for sample details. Panels (a) and (c) are otherwise identical to the baseline results in Figure 2 in the main paper (Section) but estimated in the smaller ethnicity sample. Panels (b) and (d) add ethnicity constants to the corresponding specifications to examine their role in accounting for the observed IM gaps.

The Christian-Muslim gap in upward IM is 0.08 and in downward IM 0.04; these differences are smaller than in the much larger sample in the main paper, as we miss countries with large Muslim populations and sizable religious educational gaps (like Nigeria and Cameroon) and a few censuses from sample countries. Adding in the specification the ethnicity constants lowers the gap in upward IM (to 0.06), though the downward IM difference remains unchanged. Conditioning on household size, family structure, and the relationship to the household head does not matter much, especially in the within-ethnicity comparisons. Likewise, parental occupation, the industry of employment, and household rural-urban status do not explain Muslim-Christian differences in IM, both in the specifications without and with ethnicity constants. Unlike the baseline specifications, where regional constants are the most important features explaining differences in upward and downward IM between Muslims and Christians, in the within-ethnicity specifications regional fixed effects, do not explain much of the gap, revealing African countries' considerable segregation along ethnic lines (Alesina and Zhuravskaya, 2011). The

estimates, also, suggest that the Muslim-Christian gap established in the main part of the paper remains even when one compares children of the same ethnic background, in similarly structured households, born to equally uneducated parents, working in the same sector, in the same urban/rural part of a given district. The specifications that exploit within-regional and within-ethnicity variation suggest that Muslim children, whose parents have not completed primary schooling, have a 3pps lower likelihood to finish 6 years of formal education as compared to Christian boys and girls. The Christian-Muslim difference in downward IM is about 1.5-2pps.

Turning now to the differences between Christians and Traditionalists, occupational features explain a moderate part of the gap, reflecting the differences in the industry of employment, rural-urban status, and professions between Animists and Christians (see the earlier subsection). Patterns are similar both in the cross-sectional and within-ethnicity estimates. The within-ethnicity Christian-Animist gap in upward IM is about 0.12, similar to the cross-sectional estimates. Regional fixed effects reduce inter-religious IM gaps, but less so in the within-ethnicity empirical models, consistent with ethnic segregation. The most restrictive specifications with regional and ethnic constants, as well as controls for household, occupational, and sectoral differences suggest that Animist children whose parents have not completed primary education have a 5pps lower chance to finish primary education as compared to Christian children. The within-region and within-ethnicity Christian-Animist gap in downward IM is about 2pps.

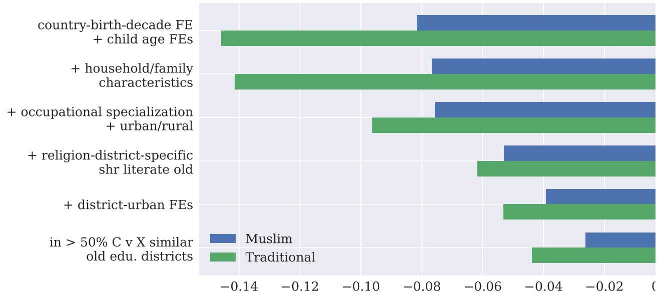

(a) Upward IM, without ethnicity FE

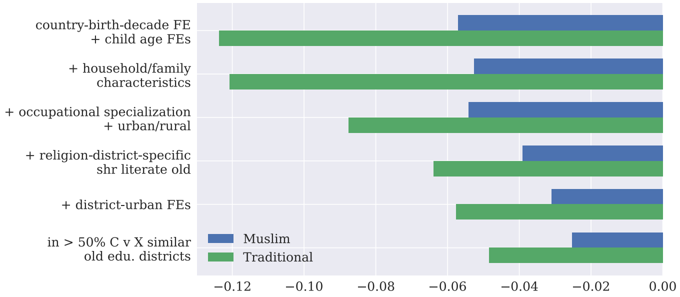

(b) Upward IM, with ethnicity FE

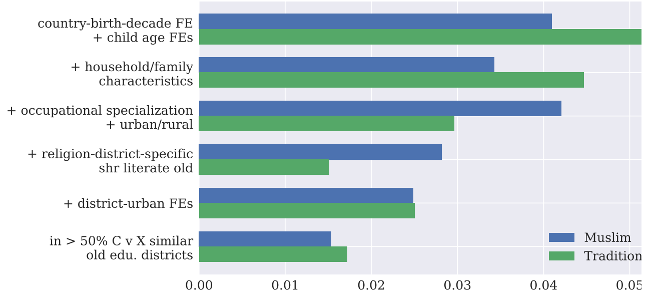

(c) Downward IM, without ethnicity FE

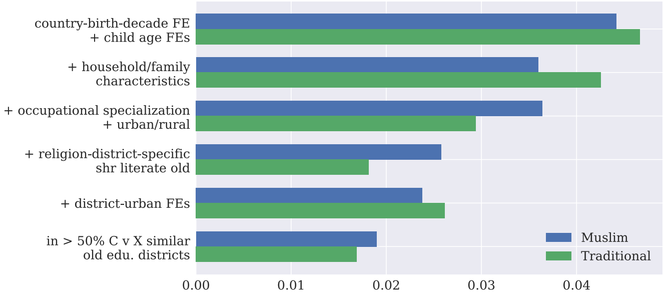

(d) Downward IM, with ethnicity FE

Figure D.3: Individual-Level Religious IM Gap. Accounting for Ethnicity

The figure shows how the Christian-Muslim (blue bars) and the Christian-Animist differences in upward IM (panels (a) and (b)) and downward IM (panels (c) and (d)) change as we add controls for the household structure (model (2)), parental occupational specialization, the industry of employment, and rural-urban residence (model (3)), and district x rural/urban fixed effects (model (4)). The bars on the top (model (1)) reflect the baseline inter-religious differences in IM, simply conditioning on country-birth-cohort fixed effects and age constants. The specifications in panels (b) and panel (d) also condition on ethnicity fixed effects (on top of the analogous empirical models in panels (a) and (c)). The sample consists of Muslim, Animist, and Christian young individuals (14-18 years old), matched to the immediately previous generation in the household. The figure gives weighted linear probability model (OLS) estimates using countries' 1980 population for the weighting to account for differential IPUMS sampling/coverage across countries.

D.3 Family Organization, and Economic Structure Inter-Religious Differences

In this subsection, we explore differences in family size, organization, structure and economic features between Christians, Muslims, and Animists. SI Figures D.4, D.5, and D.6, tabulate differences between Christians and Muslims and between Christians and Animists across family and household characteristics, while Appendix Figures D.7, D.8, and D.9 plot inter-religious differences in household urban/rural residence, older generation industry of employment, and occupation, respectively. All figures report weighted OLS specifications using countries' 1980 population as weights. We estimate the specifications in the same sample as the baseline estimates (Section). For each variable, the Figures plot two specifications on Christian-Muslim differences and two specifications on Christian-Animist differences: (i) conditioning on country-birth-decade fixed effects and (ii) conditioning on country-birth-decade fixed effects and district-urban/rural fixed effects. Two-standard-error bands, based on standard errors clustered at the country$\times$cohort level are also reported; clustering at the district level and at the cohort level yields somewhat smaller standard errors.

D.3.1 Household and Family Characteristics

Household Size Appendix Figure D.4 plots Christian-Muslim and Christian - Animist differences in household size, as reflected on the: (i) total number of household members (*# hh members*); (ii) number of household members of the "young" generation. This is captured either by IPUMS information on the relationship to household head (*rel head)* or based on the age of the individual (*age*); (iii) household members of the previous (old) generation, as reflected in the relationship to the household head variable (*rel head*) or based on the age of the individual (*age*); (iv) household members of the previous (old) generation other than the (biological) parents. The following patterns are evident. First, Muslim households are larger than Christian by one individual on average. When we compare Christian to Muslim households in the same district, the difference is about 0.75 members. The generational breakdown shows that Muslim households have on average more cohabiting members in the old (parental) generation (like uncles, aunts, and other relatives) and more in the children's generation (more siblings and young relatives like cousins). Besides, Muslim households are more likely to host the parents of the head. Second, the households of 14-18 years old Animists are also larger than Christians, by roughly 0.6 members. As with Muslims, the district of residence is important; when we compare Christian to Animist households in the same region x urban/rural the difference becomes 0.25. Compared to Christian, Animist households have more members across all three generations (household head, his/her children, and parents).

Family Structure Appendix Figure D.5 plots Christian-Muslim and Christian - Animist differences in the type of household head a typical 14-18 year old has. We distinguish between the following relationships, as recorded in IPUMS (in parentheses we report the shares of each category): (i) Mother only (14%); (ii) father only (2.3%); (iii) mother and father only (53%); (iv) other relatives only (both father and mother are absent) (13%); (v) at least one parent and other relatives (17.7%). Let's start with the Christian-Animist differences. Animist 14-18-year-old children are more likely to reside in households with at least one parent and some extended family member of the old generation, like an aunt or an uncle. However, Animist children are less likely in households without any parent, only with aunts and/or uncles. Turning

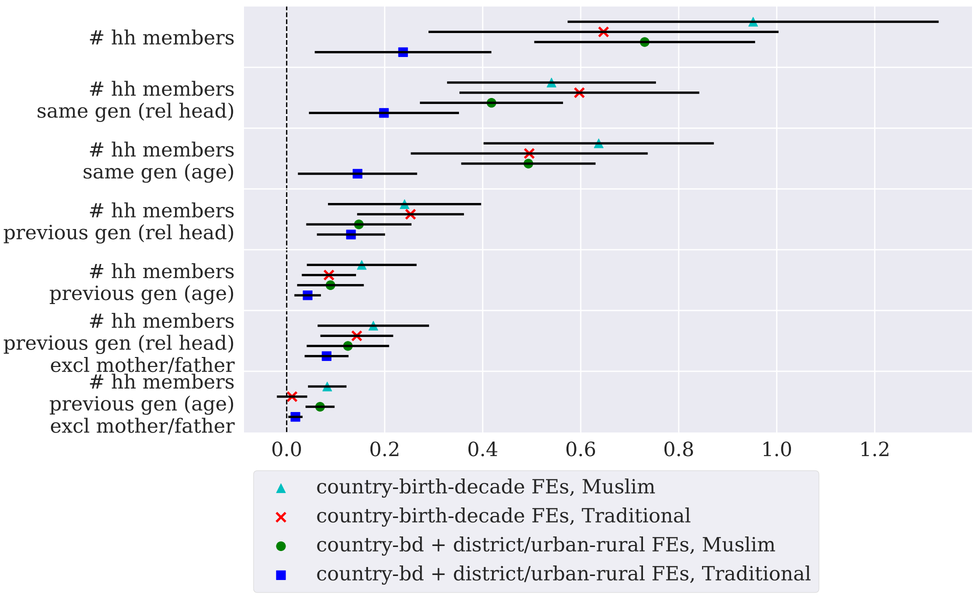

Figure D.4: Balancedness: Household Size

The figure plots weighted OLS regression estimates that associate various dimensions of household size with the religious affiliation of the young respondent. As weights, we use the country's population in 1980. The Muslim dummy variable takes the value of one for individuals adhering to Islam and zero otherwise. The Traditional dummy variable takes the value of one for individuals adhering to traditional African religions. Christian households serve as the baseline (omitted) category. There are seven measures (i) the total number of household members; (ii) the number of household members of the "young" generation, as deduced from the information on the relationship to the household head variable; (iii) the number of household members of the "young" generation, based on the age of the individual; (iv) household members of the previous (old) generation, as deduced from the information on the relationship to the household head variable; (v) household members of the previous (old) generation, based on the age of the individual; (vi) household members of the previous (old) generation other than the (biological) parents, as captured by the IPUMS-provided relationship to the household head variable; and (vii) household members of the previous (old) generation other than the (biological) parents, based on the age of the individual. The figure plots estimates from two specifications: (i) conditioning on country-birth-decade fixed effects (light blue triangles for Muslims and red marks for Animists); and (ii) conditioning on country-birth-decade fixed effects and district fixed effects interacted with rural/urban household status (green circle for Muslims and blue squares for Animists). Two-standard-error bands based on heteroskedasticity adjusted double clustered at the country x birth decade and at the province level are reported. The point estimates (green and blue dots in the figure) were obtained by running separate regressions of the district-level Christian - Muslim and Christian - Traditional IM gap, respectively, on each district-level variable (indicated on the vertical axis of the figure). The IM gap is defined as the average IM of Christians minus the average IM of Muslims or Traditionals in the district. Before running each regression, we standardize the dependent and independent variable by subtracting its sample mean and dividing by its sample standard deviation. n=6,909,663.

to Christian-Muslim differences, 14-18 years old Muslims are less likely to live in households headed by single mothers; the difference declines to 2% once we compare households in the same district. Muslim kids are less likely (by about 5%) to cohabit with both parents only, even when we look within districts, suggesting that the nuclear family structure is more common among Christians. In line with the evidence of larger Muslim households, there are considerable inter-religious differences in the likelihood of households hosting both other relatives and at least one of the parents. Compared to Christians, there is a roughly 12% increased propensity for Muslim children to co-reside with their parent(s) and other relatives. There are no significant differences among Christian, Muslim, and Animist households in the frequency of single fathers. And differences between Christian and Muslim kids cohabiting solely with other relatives are nil.

Relationship to Household Head SI Figure D.6 tabulates differences between Christians and Muslims and between Christians and Animists in terms of the relationship to the household head of 14-18 years old individuals. We distinguish between the following categories (overall shares): (i) child (34.4%); (ii)

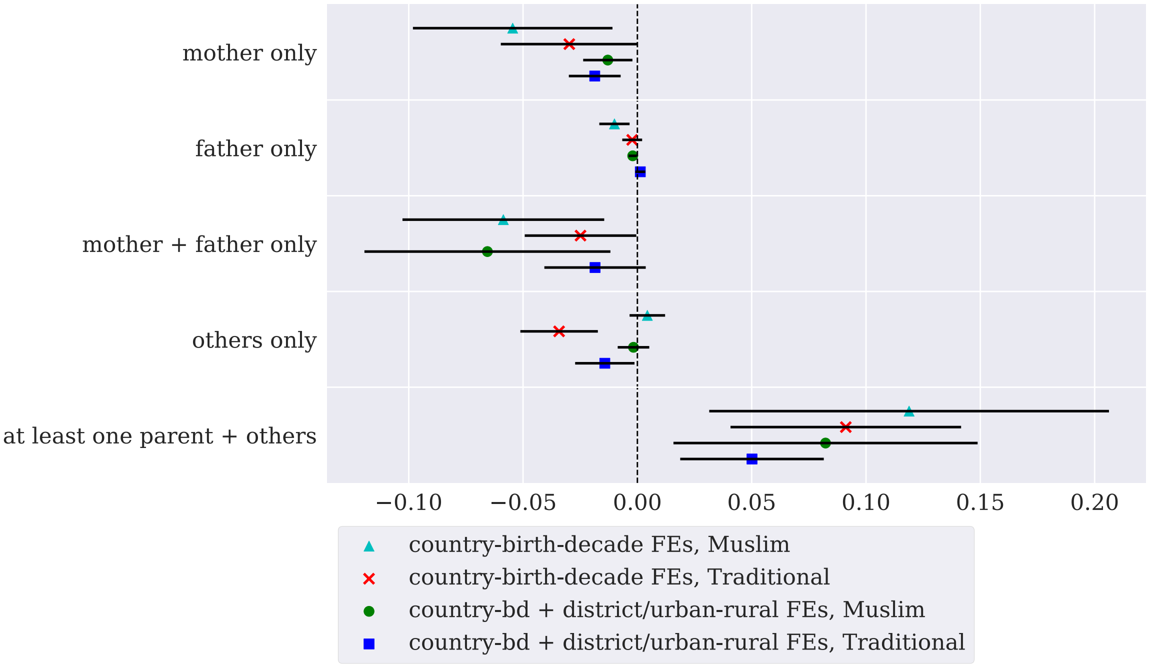

Figure D.5: Balancedness: Family Structure at the Level of the Household Head

The figure plots estimates from weighted OLS regressions that associate indicator variables of different types of family structure at the level of the household head with the religious affiliation of a young individual. As weights, we use a country's population in 1980. The Muslim variable takes the value of one for individuals adhering to Islam and zero otherwise. The Traditional variable takes the value of one for individuals adhering to traditional African religions. Christian households serve as the baseline (omitted) category. There are six family-structure categories (shares): (i) Mother only (14%); (ii) father only (2.3%); (iii) mother and father only (53%); (iv) other relatives only (both father and mother are absent) (13%); (v) at least one parent and other relatives (17.7%). The figure plots estimates from two specifications: (i) conditioning on country-birth-decade fixed effects (light blue triangles for Muslims and red marks for Animists); and (ii) conditioning on country-birth-decade fixed effects and district fixed effects interacted with rural/urban household status (green circle for Muslims and blue squares for Animists). Two-standard-error bands based on heteroskedasticity adjusted double clustered at the country x birth decade and at the province level are also reported. The point estimates (green and blue dots in the figure) were obtained by running separate regressions of the district-level Christian - Muslim and Christian - Traditional IM gap, respectively, on each district-level variable (indicated on the vertical axis of the figure). The IM gap is defined as the average IM of Christians minus the average IM of Muslims or Traditionals in the district. Before running each regression, we standardize the dependent and independent variable by subtracting its sample mean and dividing by its sample standard deviation. n=6,909,663.

biological child (49.2%) (note that the distinction between a child and a biological child reflects differences in the exact wording across censuses); (iii) foster/step/adopted child (1.1%); (iv) grandchild (3.1%); (v) household head (0.1%); (vi) spouse (1.5%); (vii) sibling (2.3%); (viii) other relative (8%); and (ix) non-relative (0.01%). There are no major differences between Muslim and Christian children in the main categories, ”child” and ”biological child” (that are about 84% of the cases). Muslim children (girls) are more likely to be spouses of the household head (by two percentage points), reflecting the tendency of girls to be married very young in Muslim communities. Besides, 14-18 year-old Animists are more likely to co-reside with their parents, compared to Christians at the expense of co-residence with other relatives and grandparents, which is lower for Animist kids.

D.3.2 Occupation and Employment Features

Urban-Rural Residence SI Figure D.7 tabulates inter-religious urban-rural differences in the households' residence; there is also a missing category, as some Censuses do not provide this information. Two patterns emerge. First, Animists have a 20-percentage-points lower (higher) likelihood to reside in

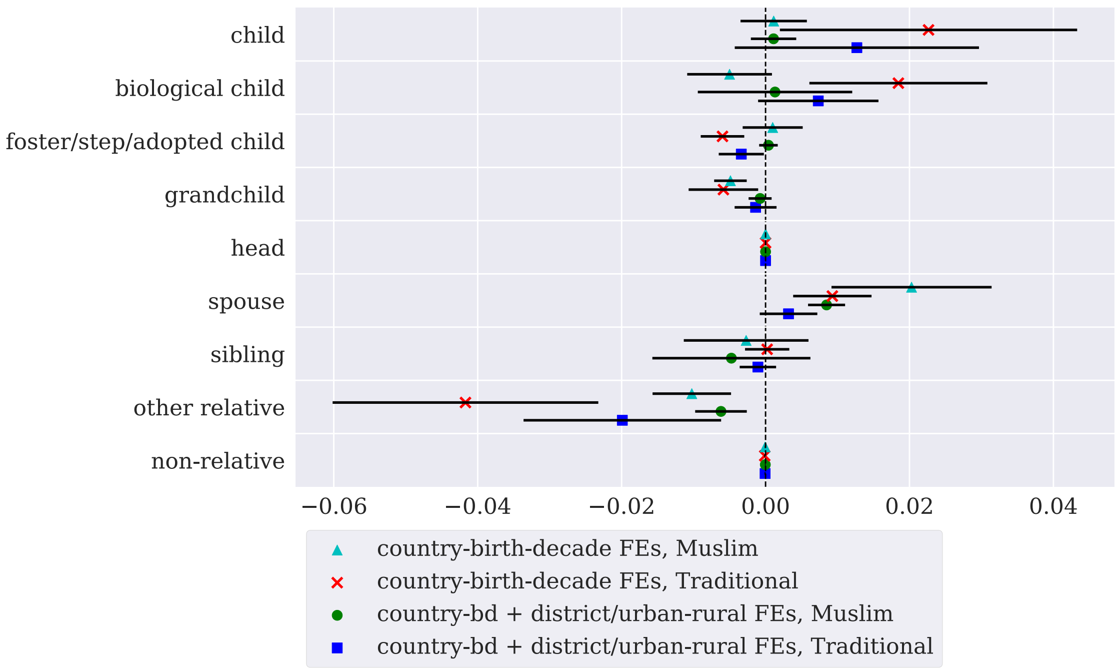

Figure D.6: Balancedness: Relationship to Household Head

The figure plots estimates from weighted OLS regressions that associate indicator variables of different relationships to household head a young individual assumes with the religious affiliation the latter. As weights, we use a country's population in 1980. The Muslim variable takes the value of one for individuals adhering to Islam and zero otherwise. The Traditional variable takes the value of one for individuals adhering to traditional African religions. Christian households serve as the baseline (omitted) category. There are nine categories in the relationship to the household head variable (we report the respective shares in parenthesis): (i) child (34.4%); (ii) biological child (49.2%) (note that the distinction between a child and a biological child reflects differences in the exact wording across censuses); (iii) foster/step/adopted child (1.1%); (iv) grandchild (3.1%); (v) household head (0.1%); (vi) spouse (1.5%); (vii) sibling (2.3%); (viii) other relative (8%); and (ix) non-relative (0.01%). The figure plots estimates from two specifications: (i) conditioning on country-birth-decade fixed effects (light blue triangles for Muslims and red marks for Animists); and (ii) conditioning on country-birth-decade fixed effects and district fixed effects interacted with rural/urban household status (green circle for Muslims and blue squares for Animists). Two-standard-error bands based on heteroskedasticity adjusted double clustered at the country x birth-decade and at the province level are also reported. The point estimates (green and blue dots in the figure) were obtained by running separate regressions of the district-level Christian - Muslim and Christian - Traditional IM gap, respectively, on each district-level variable (indicated on the vertical axis of the figure). The IM gap is defined as the average IM of Christians minus the average IM of Muslims or Traditionals in the district. Before running each regression, we standardize the dependent and independent variable by subtracting its sample mean and dividing it by its sample standard deviation. n=6,909,663.

urban (rural) locations. The difference halves when we compare Animists to Christians (the omitted category) in the same district, indicative of the residence differences between the two groups (see below). Second, there are no differences in rural-urban residence between Muslim and Christian children.

Employment Sector SI Figure D.8 tabulates inter-religious differences in the industry of employment of the old generation of 14-18 years-old children. [When we have more than one older generation member, we take the mode.] IPUMS distinguishes between the following broad industrial sectors: (i) Agriculture, fishing, and forestry, which is the largest (43%); (ii) Mining and extraction (just 0.5%); (iii) manufacturing (3.8%); (iv) utilities (3.6%); (v) construction (1.8%); and (vi) services (28%); for roughly 20% of the individuals this information is missing. Three major patterns emerge. First, there are no major differences in construction, mining, utilities, and manufacturing across religious groups. Second, members of the old generation in Animist households are more likely to work in agriculture, at the expense of services, compared to Christians. Differences are considerable, about 20 percentage points, with the district of residence differences explaining roughly half of the gap. Third, Muslim-Christian differences are smaller;

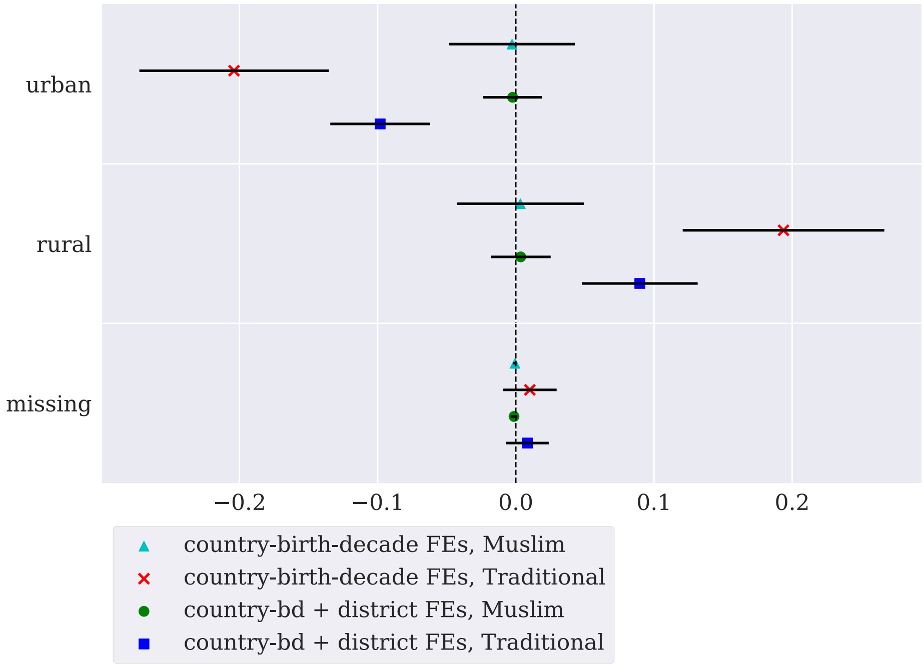

Figure D.7: Balancedness: Urban-Rural

The figure plots estimates from weighted OLS regressions that associate indicator variables of a young individual's household rural-urban status with their religious affiliation. As weights, we use a country's population in 1980. The Muslim variable takes the value of one for individuals adhering to Islam and zero otherwise. The Traditional variable takes the value of one for individuals adhering to traditional African religions. Christian households serve as the baseline (omitted) category. The figure plots estimates from two specifications: (i) conditioning on country-birth-decade fixed effects (light blue triangles for Muslims and red marks for Animists); and (ii) conditioning on country-birth-decade fixed effects and district fixed effects (green circle for Muslims and blue squares for Animists). Two-standard-error bands based on heteroskedasticity adjusted double clustered at the country x birth decade and at the province level are also reported. The point estimates (green and blue dots in the figure) were obtained by running separate regressions of the district-level Christian - Muslim and Christian - Traditional IM gap, respectively, on each district-level variable (indicated on the vertical axis of the figure). The IM gap is defined as the average IM of Christians minus the average IM of Muslims or Traditionals in the district. Before running each regression, we standardize the dependent and independent variable by subtracting its sample mean and dividing it by its sample standard deviation. n=6,909,663.

Muslims in the older generation are 3-5 percentage points more (less) likely to work in agriculture (services) than Christians.

Occupational Specialization Appendix Table D.9 tabulates inter-religious differences in the occupational specialization of the previous (parental) generation of the 14-18 years old child. [When we have more than one older generation member, we take the mode.] There are 10 main occupations, plus a missing category. (i) Legislators, senior officials, and managers (1.5%); (ii) Armed Forces (3.8%); (iii) Professionals (6.1%; (iv) Technicians and Associate Professionals (1.8%); (v) Clerks (8.8%); (vi) Service Workers (47%); (vii) Skilled agriculture and fishery (5.1%); (viii) Crafts and related trades workers (2.3%); (ix) Plant and machine operators (0.1%; and (x) Elementary occupations (4.6%); 18.7% is missing. The test of means reveals the following regularities. First, Animists and to a lesser extent Muslims are less likely to work as professionals than Christians; however, this is a small category, as only 6% of Africans are professionals. Second, there are some inter-religious differences in clerks and associate professionals. Third, Animists are considerably more likely (by about 20pps) to work in agriculture and fishery. However, regional features explain most of this difference. Fourth, Animists are under-represented as service and sales workers by about 20pps. Fifth, Muslim-Christian differences are minimal in all cate-

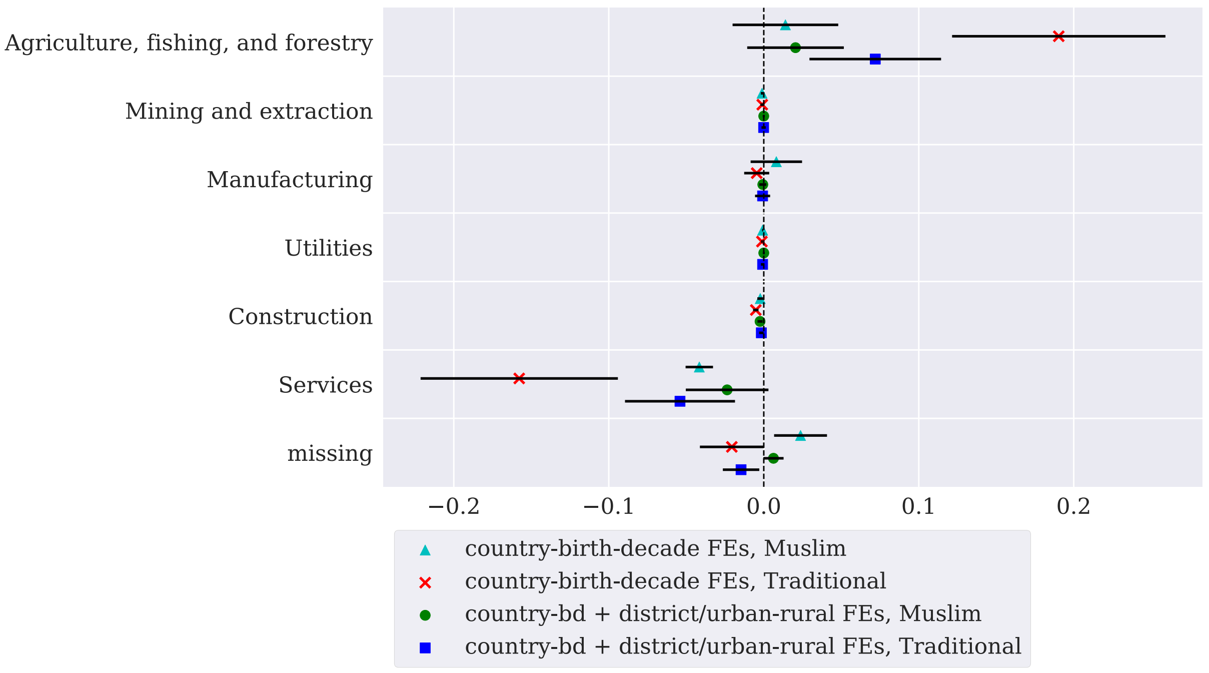

Figure D.8: Balancedness: Previous Generation Industry of Employment

The figure plots estimates from weighted OLS regressions that associate indicator variables of the sector of employment of the previous generation household members (parent's generation) with the religious affiliation of the young in the household. As weights, we use a country's population in 1980. The Muslim variable takes the value of one for individuals adhering to Islam and zero otherwise. The Traditional variable takes the value of one for individuals adhering to traditional African religions. Christian households serve as the baseline (omitted) category. There are six sectors of employment (shares): (i) Agriculture, fishing, and forestry, which is the largest (43%); (ii) Mining and extraction (just 0.5%); (iii) manufacturing (3.8%); (iv) utilities (3.6%); (v) construction (1.8%); and (vi) services (28%); for roughly 20% of the sample, this information is missing. The figure plots estimates from two specifications: (i) conditioning on country-birth-decade fixed effects (light blue triangles for Muslims and red marks for Animists); and (ii) conditioning on country-birth-decade fixed effects and district fixed effects interacted with rural-urban residence (green circle for Muslims and blue squares for Animists). Two-standard-error bands based on heteroskedasticity adjusted double clustered at the country x birth decade and at the province level are also reported. The point estimates (green and blue dots in the figure) were obtained by running separate regressions of the district-level Christian - Muslim and Christian - Traditional IM gap, respectively, on each district-level variable (indicated on the vertical axis of the figure). The IM gap is defined as the average IM of Christians minus the average IM of Muslims or Traditionals in the district. Before running each regression, we standardize the dependent and independent variable by subtracting its sample mean and dividing it by its sample standard deviation. n=6,909,663.

gories but professionals. Interestingly, Muslims within districts are somewhat more likely to be traders and engaged in shop and market services more generally.

D.4 Matching Districts. Old Generation's Education by Religion

Our exploration of the role of family/household size and structure, income proxies of the household head, and regional features reveals that regional factors explain roughly half of the non-negligible differences in upward IM and downward IM between Christians and Muslims and between Christians and Animists. Besides, the education of the old in the district is a significant correlate of children's attainment (Alesina et al., 2021). However, even when we compare Christians to Muslims and Traditionalists in the same district and condition on their own group's old-generation primary school completion (and other observable household features), there is a significant gap in children's educational attainment. In an effort to better account for religion-specific regional educational dynamics and hard-to-observe religious differences in initial access to schools, we repeated the analysis restricting estimation to districts with small inter-religious differences in education across the old generation.

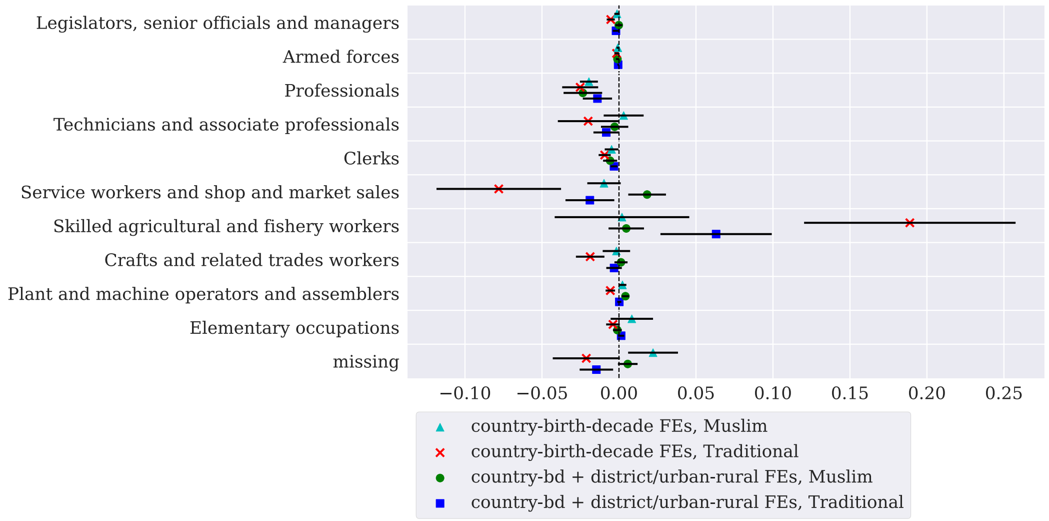

Figure D.9: Balancedness: Previous Generation Occupation

The figure plots estimates from weighted OLS regressions that associate indicator variables reflecting the profession of the previous generation household members (parent's generation) with the religious affiliation of the young in the household. As weights, we use a country's population in 1980. The Muslim variable takes the value of one for individuals adhering to Islam and zero otherwise. The Traditional variable takes the value of one for individuals adhering to traditional African religions. Christian households serve as the baseline (omitted) category. There are ten occupational categories (shares): (i) Legislators, senior officials, and managers (1.5%); (ii) Armed Forces (3.8%); (iii) Professionals (6.1%; (iv) Technicians and Associate Professionals (1.8%); (v) Clerks (8.8%); (vi) Service Workers (47%); (vii) Skilled agriculture and fishery (5.1%); (viii) Crafts and related trades workers (2.3%); (ix) Plant and machine operators (0.1%; and (x) Elementary occupations (4.6%); 18.7% is missing. The figure plots estimates from two specifications: (i) conditioning on country-birth-decade fixed effects (light blue triangles for Muslims and red marks for Animists); and (ii) conditioning on country-birth-decade fixed effects and district fixed effects interacted with rural-urban residence (green circle for Muslims and blue squares for Animists). Two-standard-error bands based on heteroskedasticity adjusted double clustered at the country x birth decade and at the province level are reported. The point estimates (green and blue dots in the figure) were obtained by running separate regressions of the district-level Christian - Muslim and Christian - Traditional IM gap, respectively, on each district-level variable (indicated on the vertical axis of the figure). The IM gap is defined as the average IM of Christians minus the average IM of Muslims or Traditionals in the district. Before running each regression, we standardize the dependent and independent variable by subtracting its sample mean and dividing it by its sample standard deviation. n=6,909,663.

Specifically, we focused on 50% of all districts where inter-religious differences between the old generation's completed primary schooling are the smallest. In Figure 2 and in this appendix section's figures we report estimates looking at half of a country's districts. Focusing on this sample makes the previous-generation education distributions between Christians and Muslims and between Christians and Animists more comparable, as the mass of the distribution of gaps in terms of primary school completion rates for the old generation shifts closer to zero for each country-religion-pair. SI Figure D.10 illustrates our approach, plotting for each country, distributional differences (Wasserstein distances) in the old generation's completed primary between Christians and Muslims (panel (a)) and between Christians and Animists (panel (b)) for the 1980s-cohort. [As the share of Africans adhering to Traditional religions is about 3% the observations for Animists in panel (b) per district are not always large.] Each panel gives two distributions [Gaussian kernel densities] of Wasserstein distances per country. [1] First for all districts [full sample in blue]. second, for half of the total districts in a country, where inter-religious differences in old's education are the smallest [in red]. The comparison of the two distributions, therefore, shows the effect of reducing, for each country, the sample to districts whose previous-generation years-of-schooling

[1] Given two distributions u and v, the Wasserstein distance equals the minimum amount of "work" needed to transform u into v, where work is defined as the amount of probability mass moved times the distance it is moved by. For this reason, the Wasserstein distance is also called the "earth mover's distance." In the one-dimensional case, the Wasserstein distance is equivalent to the integral over the absolute value of the difference in the CDFs of the two distributions: $W(u,v) = \int_{\mathbb{R}} |U(x) - V(x)|\, dx$.

distributions are among the 50% closest for a given religion-pair.

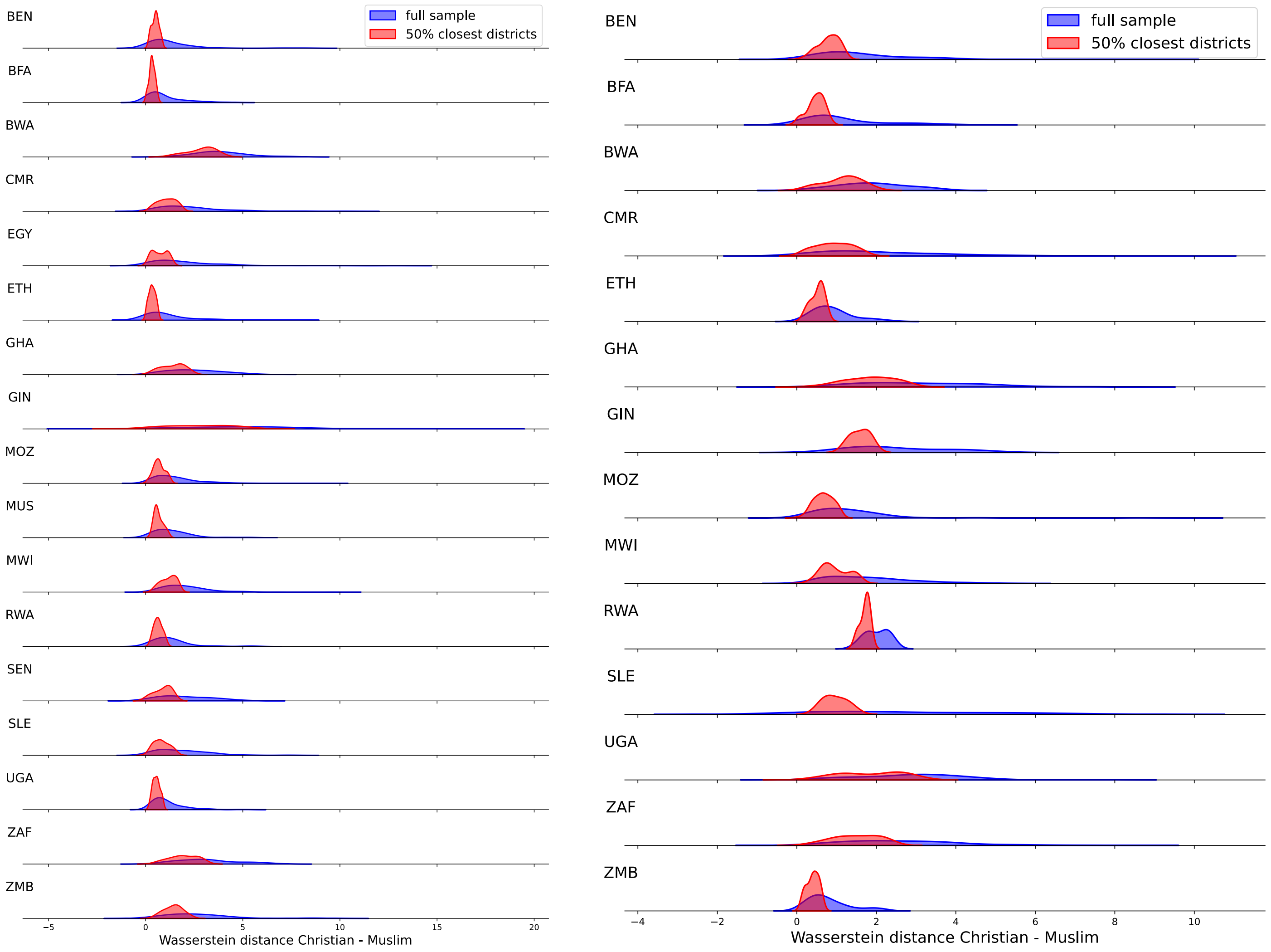

(a) Christian-Muslim Differences

(b) Christian-Animist Differences

Figure D.10: District-Level Parental Education Distribution Comparisons

The figures show the effect of reducing the sample to those districts whose previous-generation years-of-schooling distributions are among the 50% closest for a given religion-pair in each country. Panel (a) looks at Christian-Muslim district-level differences in years of schooling, while panel (b) at Christian-Animist district-level differences in years of schooling. The panels plot for each country and religion-pair, two Gaussian kernel density estimates of the distributions of Wasserstein distances of the district-level years-of-schooling distributions for the 1980s-born. In blue, the figures plot the country-level distribution of Wasserstein distances [each Wasserstein distance is the distributional difference within a district for a given religion pair] in the full sample of districts (in a given country). In red, the figures plot the distribution of Wasserstein distances across half (50%) of total districts with the closest education distributions.

E Childhood Regional Exposure Effects

This Section reports further evidence and sensitivity checks for our analysis of religion-specific regional childhood exposure effects reported in Section of the main paper (Figure 3). First, E.1 compares the sample of movers with non-movers. Second, E.2 presents parametric estimates on regional childhood exposure effects. Third, E.3 gives estimates distinguishing by gender.

E.1 Country Sample

For the implementation of the movers' design that teases apart childhood exposure regional effects from spatial sorting, one needs data on internal migration, place of birth and current residence, *and* information on length (years) of residence in the current location. IPUMS provides this information for 13 countries: Benin, Cameroon, Egypt, Ethiopia, Ghana, Guinea, Mali, Malawi, Rwanda, Togo, Uganda, South Africa, and Zambia. Compared to 21 countries we work with in the other parts of the paper, we miss data from 8 countries, namely: Botswana (2001, 2011), Liberia (2008), Mozambique (2007), Mauritius (1990, 2000, 2011), Nigeria (2010), Senegal (1988, 2002), Sierra Leone (2004), and Togo (1970, 2010), see SI Table B.2. [The Nigerian household surveys do not record birthplace. The censuses of the other seven countries, while they give birthplace and current residence, do not provide information on time in current residence.]

E.2 Parametric Estimates and Sensitivity Analysis

Specification Besides the semi-parametric estimates of childhood exposure regional effects in the main paper, following Chetty and Hendren (2018), we also run parametric estimates imposing a piece-wise linear structure between ages $(1-11)$ and the years less relevant for primary schooling completion $(12-18)$ (Alesina et al., 2021). The specification reads:

$$\begin{aligned}\text{IM\_up}_{ihbmcod} = {} & [\psi_h +] \sum_{b=b_0}^{B} \mathbb{I}(b_i = b) \times \left(\alpha_b^1 + \alpha_b^2 \times \widehat{\text{IM\_up}}_{ob}^{\text{nm}}\right) + \\ & \sum_{m=1}^{18} \zeta_m \times \mathbb{I}(m_i = m) + \sum_{b=b_0}^{B} \kappa_b \times \mathbb{I}(b_i = b) \times \Delta_{odb} + \\ & \mathbb{I}(m_i < 12) \times (\beta_0 + (18 - m_i) \times \beta_1) \times \Delta_{odb} + \\ & \mathbb{I}(m_i \geq 12) \times (\delta_0 + (18 - m_i) \times \delta_1) \times \Delta_{odb} + \epsilon_{ihbmcod}.\end{aligned}$$

Parametric Cross-sectional and Within-Household Estimates SI Table E.1 reports the semi-parametric estimates. For brevity, we report specifications using differences in upward educational mobility between origin and destination districts among non-movers, Δ_{odb}^{all}. Odd-numbered columns report cross-sectional estimates. Even-numbered columns add household fixed effects $[\psi_h]$; therefore, these specifications exploit variation between siblings whose families moved when they were at different ages. Regional childhood exposure effects are about two percentage points per year for Christians and somewhat smaller for Muslims. The estimate for Animists is smaller, about 1.1% and more imprecise, reflecting the low

number of observations and the heterogeneous nature of indigenous religions. The pattern is similar when we augment the specification with household fixed effects to account for unobserved family features related to the valuation of education, ethnic features, and social norms. Comparing siblings yields regional childhood exposure effects of about 2.3% for Christians, similar but imprecise for Animists, and about 1.85% for Muslims.

Dependent Variable:	upward IM					
	Christian		Muslim		Traditional	
Model:	(1)	(2)	(3)	(4)	(5)	(6)
Variables						
exposure ages 1-11	0.0228***	0.0235***	0.0157***	0.0184***	0.0118	0.0236
	(0.0052)	(0.0057)	(0.0051)	(0.0069)	(0.0121)	(0.0298)
exposure ages 12-18	-0.0080	0.0031	-0.0098	-0.0151	-0.0062	-0.0667*
	(0.0081)	(0.0093)	(0.0143)	(0.0096)	(0.0262)	(0.0360)
Fixed-effects						
birth-decade	Yes	Yes	Yes	Yes	Yes	Yes
age-at-migration	Yes	Yes	Yes	Yes	Yes	Yes
household	No	Yes	No	Yes	No	Yes
Fit statistics						
Observations	141,355	141,355	128,215	128,215	7,116	7,116
R^2	0.12212	0.82261	0.11252	0.82461	0.17014	0.85483
Within R^2	0.09573	0.00789	0.07677	0.00430	0.13066	0.00767

Table E.1: Parametric Estimates. Regional Childhood Exposure by Religion, Overall Δ_{odb}

The table reports LS estimates. The dependent variable is an indicator variable that takes the value of one for 14-25 years old individuals with completed (at least) primary education, cohabiting with previous generation member(s), usually parents, who did not complete primary schooling (upward educational IM). Columns (1)-(2) report specifications for Christians, columns (3)-(4) for Muslims, and columns (5)-(6) for Africans adhering to traditional religions. In all specifications, destination minus origin differences in educational upward IM is estimated using non-movers of all religious affiliations. The control variables comprise a linear origin-average-IM (calculated for the birth-cohort relevant to the individual among non-movers) term, age-at-move indicator variables, birth-decade x destination indicators interacted with destination-minus-origin differences in upward IM, all of which are not reported, and two linear terms for destination-minus-origin differences in the relevant birth- cohort-non-mover average IM for moves taking place when the child moves, ages 1-11 and 12-18. The specifications in columns (2), (4), and (6) also include household fixed effects. Double clustered at the origin and at the destination district standard errors are reported in parentheses. ***,** ,*, denote significance at 1%, 5%, and 10% levels, respectively, in a two-sided test of the null hypothesis of exposure effects equal to zero, using the t-statistic. Test statistics are not adjusted for multiple comparisons.

Robustness We perturbed the empirical model in various ways to explore the sensitivity of our regional childhood exposure effect estimates. In SI Table E.2 we drop multi-generational households (with grandparents), while in SI Table E.3 we only look at children matched-to-parents (no uncles, aunts, etc). By doing so, we minimize concerns that the baseline estimates reflect differences in household arrangements and family structure across religious affiliations. Regional childhood exposure effects are about 2.1-2.5% for Christians and 1.3%-1.7% for Muslims. The estimates for Animists are smaller, unstable, and statistically indistinguishable from zero. As the number of observations is small, the data does not allow us to credibly assess whether the insignificance reflects actual weak regional exposure effects for Africans adhering to local religions when moving to high-mobility regions or imprecision due to heterogeneity and small-sample issues.

Dependent Variable:	upward IM					
	Christian		Muslim		Traditional	
Model:	(1)	(2)	(3)	(4)	(5)	(6)
Variables						
exposure ages 1-11	0.0243***	0.0262***	0.0163***	0.0171**	0.0049	-0.0127
	(0.0051)	(0.0069)	(0.0062)	(0.0076)	(0.0139)	(0.0348)
exposure ages 12-18	-0.0086	0.0030	-0.0080	-0.0141	0.0040	-0.1014**
	(0.0082)	(0.0103)	(0.0165)	(0.0122)	(0.0323)	(0.0458)
Fixed-effects						
birth-decade	Yes	Yes	Yes	Yes	Yes	Yes
age-at-migration	Yes	Yes	Yes	Yes	Yes	Yes
household	No	Yes	No	Yes	No	Yes
Fit statistics						
Observations	111,278	111,278	101,536	101,536	5,165	5,165
R^2	0.12708	0.84112	0.11157	0.84744	0.16981	0.86649
Within R^2	0.10191	0.00805	0.07449	0.00334	0.12785	0.01087

Table E.2: Regional Childhood Exposure by Religion, Overall Δ_{odb}
Excluding Multigenerational Households

The table reports LS estimates. The dependent variable is an indicator variable that takes the value of one for 14-25 years old individuals with completed (at least) primary education, cohabiting with previous generation member(s), usually parents, who did not complete primary schooling (upward educational IM). Columns (1)-(2) report specifications for Christians, columns (3)-(4) for Muslims, and columns (5)-(6) for Africans adhering to traditional religions. In all specifications, destination minus origin differences in educational upward IM is estimated using non-movers of all religious affiliations. The control variables comprise a linear origin-average-IM (calculated for the birth-cohort relevant to the individual among non-movers) term, age-at-move indicator variables, birth-decade x destination indicators interacted with destination-minus-origin differences in upward IM, all of which are not reported, and two linear terms for destination-minus-origin differences in the relevant birth-cohort-non-mover average IM for moves taking place when the child moves, ages 1-11 and 12-18. The specifications in columns (2), (4), and (6) also include household fixed effects. In all specifications, we drop multi-generational households, defined as those with more than two generations (e.g., with grandparents). Double clustered at the origin and at the destination district standard errors are reported in parentheses. ***,** ,*, denote significance at 1%, 5%, and 10% levels, respectively, in a two-sided test of the null hypothesis of exposure effects equal to zero, using the t-statistic. Test statistics are not adjusted for multiple comparisons.

Dependent Variable:	upward IM					
	Christian		Muslim		Traditional	
Model:	(1)	(2)	(3)	(4)	(5)	(6)
Variables						
exposure ages 1-11	0.0204***	0.0213**	0.0173**	0.0130*	-0.0086	-0.0149
	(0.0054)	(0.0093)	(0.0070)	(0.0077)	(0.0196)	(0.0466)
exposure ages 12-18	-0.0148	0.0100	-0.0131	-0.0270	0.0616*	-0.0475
	(0.0100)	(0.0131)	(0.0158)	(0.0167)	(0.0349)	(0.0555)
Fixed-effects						
birth-decade	Yes	Yes	Yes	Yes	Yes	Yes
age-at-migration	Yes	Yes	Yes	Yes	Yes	Yes
household	No	Yes	No	Yes	No	Yes
Fit statistics						
Observations	71,987	71,987	77,934	77,934	3,154	3,154
R^2	0.12478	0.83267	0.07362	0.82714	0.14136	0.85020
Within R^2	0.10310	0.00807	0.05552	0.00209	0.09941	0.01214

Table E.3: Regional Childhood Exposure Effect by Religion, Overall Δ_{odb}
Young Individuals Matched to (Biological) Parents

The table reports LS estimates. The dependent variable is an indicator variable that takes the value of one for 14-25 years old individuals with completed (at least) primary education, cohabiting with their bilogical parent(s), who did not complete primary schooling (upward educational IM). Columns (1)-(2) report specifications for Christians, columns (3)-(4) for Muslims, and columns (5)-(6) for Africans adhering to traditional religions. In all specifications, destination minus origin differences in educational upward IM is estimated using non-movers of all religious affiliations. The control variables comprise a linear origin-average-IM (calculated for the birth-cohort relevant to the individual among non-movers) term, age-at-move indicator variables, birth-decade x destination indicators interacted with destination-minus-origin differences in upward IM, all of which are not reported, and two linear terms for destination-minus-origin differences in the relevant birth- cohort-non-mover average IM for moves taking place when the child moves, ages 1-11 and 12-18. The specifications in columns (2), (4), and (6) also include household fixed effects. In all specifications, we only look at children matched to biological parents. Double clustered at the origin and at the destination district standard errors are reported in parentheses. ***,** ,*, denote significance at 1%, 5%, and 10% levels, respectively, in a two-sided test of the null hypothesis of exposure effects equal to zero, using the t-statistic. Test statistics are not adjusted for multiple comparisons.

E.3 Gender Differences

SI Tables E.4-E.5 explore gender differences in regional childhood exposure effects for Christians (columns (1)-(2), Muslims (columns (3)-(4)) and Animists (columns (5)-(6)) employing the piece-wise linear specification of Chetty and Hendren (2018).

Two patterns emerge. First, regional childhood exposure effects are somewhat larger for both Christian boys and girls, as compared to Muslim boys and girls. Second, there are no major differences between Christian boys and girls nor between Muslim boys and girls.

Dependent Variable:	upward IM					
	Christian		Muslim		Traditional	
Model:	(1)	(2)	(3)	(4)	(5)	(6)
Variables						
exposure ages 1-11	0.0197***	0.0317***	0.0095	0.0149*	0.0095	0.0319
	(0.0051)	(0.0094)	(0.0064)	(0.0083)	(0.0182)	(0.0478)
exposure ages 12-18	-0.0041	0.0082	-0.0074	-0.0161	0.0290	-0.0946
	(0.0103)	(0.0164)	(0.0239)	(0.0120)	(0.0341)	(0.0651)
Fixed-effects						
birth-decade	Yes	Yes	Yes	Yes	Yes	Yes
age-at-migration	Yes	Yes	Yes	Yes	Yes	Yes
household	No	Yes	No	Yes	No	Yes
Fit statistics						
Observations	70,442	70,442	66,900	66,900	3,437	3,437
R^2	0.10114	0.87836	0.07225	0.87221	0.15702	0.90300
Within R^2	0.07857	0.00920	0.05383	0.00421	0.11062	0.03307

Table E.4: Regional Childhood Exposure Effects by Religion for Boys, Overall Δ_{odb}

The table reports LS estimates. The dependent variable is an indicator variable that takes the value of one for 14-25 years old boys with completed (at least) primary education, cohabiting with previous generation member(s), usually parents, who did not complete primary schooling (upward educational IM). Columns (1)-(2) report specifications for Christians, columns (3)-(4) for Muslims, and columns (5)-(6) for Africans adhering to traditional religions. In all specifications, destination minus origin differences in educational upward IM is estimated using non-movers of all religious affiliations. The control variables comprise a linear origin-average-IM (calculated for the birth-cohort relevant to the individual among non-movers) term, age-at-move indicator variables, birth-decade x destination indicators interacted with destination-minus-origin differences in upward IM, all of which are not reported, and two linear terms for destination-minus-origin differences in the relevant birth-cohort-non-mover average IM for moves taking place when the child moves, ages 1-11 and 12-18. The specifications in columns (2), (4), and (6) also include household fixed effects. Double clustered at the origin and at the destination district standard errors are reported in parentheses. ***,** ,*, denote significance at 1%, 5%, and 10% levels, respectively, in a two-sided test of the null hypothesis of exposure effects equal to zero, using the t-statistic. Test statistics are not adjusted for multiple comparisons.

Dependent Variable:	upward IM					
	Christian		Muslim		Traditional	
Model:	(1)	(2)	(3)	(4)	(5)	(6)
Variables						
exposure ages 1-11	0.0260***	0.0229*	0.0241***	0.0160	0.0124	0.0053
	(0.0065)	(0.0130)	(0.0065)	(0.0117)	(0.0181)	(0.0561)
exposure ages 12-18	-0.0087	0.0027	-0.0025	-0.0115	-0.0414	-0.1506***
	(0.0096)	(0.0133)	(0.0133)	(0.0140)	(0.0331)	(0.0472)
Fixed-effects						
birth-decade	Yes	Yes	Yes	Yes	Yes	Yes
age-at-migration	Yes	Yes	Yes	Yes	Yes	Yes
household	No	Yes	No	Yes	No	Yes
Fit statistics						
Observations	70,913	70,913	61,315	61,315	3,679	3,679
R^2	0.14981	0.91294	0.16211	0.92271	0.19003	0.93365
Within R^2	0.11745	0.01111	0.10813	0.00971	0.15009	0.03734

Table E.5: Regional Childhood Exposure Effects by Religion for Girls, Overall Δ_{odb}

The table reports LS estimates. The dependent variable is an indicator variable that takes the value of one for 14-25 years old girls with completed (at least) primary education, cohabiting with previous generation member(s), usually parents, who did not complete primary schooling (upward educational IM). Columns (1)-(2) report specifications for Christians, columns (3)-(4) for Muslims, and columns (5)-(6) for Africans adhering to traditional religions. In all specifications, destination minus origin differences in educational upward IM is estimated using non-movers of all religious affiliations. The control variables comprise a linear origin-average-IM (calculated for the birth-cohort relevant to the individual among non-movers) term, age-at-move indicator variables, birth-decade x destination indicators interacted with destination-minus-origin differences in upward IM, all of which are not reported, and two linear terms for destination-minus-origin differences in the relevant birth-cohort-non-mover average IM for moves taking place when the child moves, ages 1-11 and 12-18. The specifications in columns (2), (4), and (6) also include household fixed effects. Double clustered at the origin and at the destination district standard errors are reported in parentheses. ***,** ,*, denote significance at 1%, 5%, and 10% levels, respectively, in a two-sided test of the null hypothesis of exposure effects equal to zero, using the t-statistic. Test statistics are not adjusted for multiple comparisons.

F Variable Sources and Definitions

This Supplementary Information (SI) Section gives variable definitions and sources for all regional correlates of educational intergenerational mobility (IM).

F.1 At-Independence Features

ln(population density 1950) The natural logarithm of mean population density in the district in 1950; the latter is computed, as the within-district zonal statistic of a raster provided by Klein Goldewijk et al. (2010), which we resample to a resolution of 30 arc-seconds prior computing the statistic.

urban share (born < 1960) The share of the (non-migrant) district population born prior to 1960 classified as urban at the time of the census. IPUMS census data.

agri. empl share (born < 1960) The share of the (non-migrant) district population born prior to 1960 and working in agriculture at the time of the census. IPUMS census data.

manuf.-serv. empl share (born < 1960) The share of the (non-migrant) district population born prior to 1960 and working in manufacturing and services at the time of the census. IPUMS census data.

F.2 Geographical and Location Features

ln(distance to the capital) The natural logarithm of the geodesic distance from the district centroid to the national capital. Computed using GIS software.

ln(distance to the border) The natural logarithm of the geodesic distance from the district centroid to the closest point on the national border. Computed using GIS software.

ln(distance to the coast) The natural logarithm of the geodesic distance from the district centroid to the closest point on the coastline. Computed using GIS software.

ln(1+malaria stability) The natural logarithm of 1 + mean stability of malaria transmission in the district. The latter variable is computed, using GIS software, as the within-district zonal statistic of a raster provided by Kiszewski et al. (2004), which we re-sample to a resolution of 30 arc-seconds prior to computing the statistic.

ln(1+agricultural suitability) The natural logarithm of 1 + mean agricultural suitability in the district. The latter variable is computed, using GIS software, as the within-district zonal statistic of a raster provided by Ramankutty et al. (2002), which we re-sample to a resolution of 30 arc-seconds prior to computing the statistic.

ln(terrain ruggedness) The natural logarithm of terrain ruggedness. The latter is computed using cell-level data on elevation at 30 arc-second resolution from the U.S. Geological Survey (1996). Given the grid cell data, picture a 3×3 block of 9 cells and let $e_{r,c}$ be the elevation of the cell in row r, column c of the grid. Following Nunn and Puga (2012), we compute ruggedness as $\sqrt{\sum_{i=r-1^{r+1}} \sum_{i=c-1}^{c+1} (e_{i,j} - e_{r,c})^2}$; the square root of the sum of all squared differences in elevation between the middle cell and the surrounding 8 cells.

oil An indicator equal to 1 if the district is intersected by an oil field, and zero otherwise. Data on oil fields come from Lujala et al. (2007)

diamond mine An indicator equal to 1 if the district is intersected by a diamond mine, and zero other-

wise. Data come from Lujala et al. (2005)

F.3 Historical Features

ln(distance to the railroad) The natural logarithm of the geodesic distance from the district centroid to the closest point on a colonial railroad. Computed using GIS software. Data on colonial railroads come from Jedwab and Moradi (2016).

ln(distance to road) The natural logarithm of the geodesic distance from the district centroid to the closest point on a colonial road. Computed using GIS software. Data on colonial roads come from Jedwab and Storeygard (2022).

ln(distance to Catholic mission) The natural logarithm of the geodesic distance from the district centroid to the closest Catholic Mission. Computed using GIS software. Data on missions come from Nunn (2010).

ln(distance to Protestant mission) The natural logarithm of the geodesic distance from the district centroid to the closest Protestant Mission. Computed using GIS software. Data on missions come from Nunn (2010), Cagé and Rueda (2016).

ln(distance to precolonial state) The natural logarithm of the geodesic distance from the district centroid to the closest pre-colonial state. Data on pre-colonial states are obtained by combining maps of pre-colonial ethnic homelands with the levels of jurisdictional hierarchy beyond the local community level in Murdock (1967). Societies with 3 or 4 levels are classified as states.

F.4 Religious Composition

Fractionalization One minus Herfindahl-Hirschman index of religious concentration (sum of the three main religion shares squared). The index reflects the likelihood that two randomly chosen individuals of any age in a given administrative unit will not adhere to the same faith. Source: IPUMS.

Own Religion Share The share of each religion in a district's population, estimated separately for Muslims, Christians, and Animists of the same birth cohort. As IPUMS reports person weights we take the share of the sum of person weights for each religious group to the sum of all person weights in a district-cohort. Source: IPUMS.

SI References

David Card. The Causal Effect of Education on Earnings. In *Handbook of Labor Economics*, volume 3, Part A, pages 1801–1863. Elsevier, 1999.

Alwyn Young. The African Growth Miracle. *Journal of Political Economy*, 120(4):696–739, August 2012. ISSN 0022-3808. doi: 10.1086/668501.

Francesco Caselli, Jacopo Ponticelli, and Federico Rossi. A new data set on mincerian returns. *Technology differences over space and time*, pages 108–118, 2014.

George Psacharopoulos. Returns to Investment in Education: A Global Update. *World Development*, 22 (9):1325–1343, September 1994. ISSN 0305-750X. doi: 10.1016/0305-750X(94)90007-8.

Girmay Tsegay Kiross, Catherine Chojenta, Daniel Barker, Tenaw Yimer Tiruye, and Deborah Loxton. The effect of maternal education on infant mortality in ethiopia: A systematic review and meta-analysis. *PLOS One*, 14(7):1–8, 2019.

Dessalegn Melesse, Martin K Mutua, Allysha Choudhury, Yohannes D Wado, Cheikh M Faye, Sarah Neal, and Ties Boerma. Adolescent sexual and reproductive health in sub-saharan africa: who is left behind? *BMJ Global Health*, 5(1):1–12, 2020.

Tom Vogl. Fertility and the education of african parents and children. NBER Working Papaer 30474, September 2022.

Esther Duflo, Pascaline Dupas, and Michael Kremer. Education, hiv, and early fertility: Experimental evidence from kenya. *American Economic Review*, 105(9):2767–2797, 2015.

Nathan Nunn and Diego Puga. Ruggedness: The Blessing of Bad Geography in Africa. *The Review of Economics and Statistics*, 94(1):20–36, 2012.

Robert J. Barro and Jong Wha Lee. A New Data Set of Educational Attainment in the World, 1950–2010. *Journal of Development Economics*, 104(C):184–198, 2013.

Timothy Besley and Torsten Persson. State Capacity, Conflict, and Development. *Econometrica*, 78(1): 1–34, 2010.

Nicola Gennaioli and Ilia Rainer. The Modern Impact of Precolonial Centralization in Africa. *Journal of Economic Growth*, 12(3):185–234, 2007.

Raj Chetty and Nathaniel Hendren. The Impacts of Neighborhoods on Intergenerational Mobility II: County-Level Estimates. *The Quarterly Journal of Economics*, 133(3):1163–1228, August 2018. ISSN 0033-5533. doi: 10.1093/qje/qjy006.

Remi Jedwab and Alexander Moradi. The Permanent Effects of Transportation Revolutions in Poor Countries: Evidence from Africa. *The Review of Economics and Statistics*, 98(2):268–284, 2016.

Remi Jedwab and Adam Storeygard. The Average and Heterogeneous Effects of Transportation Investments: Evidence from Sub-Saharan Africa. *Journal of the European Economic Association*, 20:1–38, 2022.

Nathan Nunn. Religious Conversion in Colonial Africa. *American Economic Review*, 100(2):147–152, May 2010. ISSN 0002-8282. doi: 10.1257/aer.100.2.147.

George Peter Murdock. Ethnographic Atlas: A Summary. *Ethnology*, 6(2):109–236, 1967. ISSN 0014-1828. doi: 10.2307/3772751.

Kees Klein Goldewijk, Arthur Beusen, and Peter Janssen. Long-Term Dynamic Modeling of Global Population and Built-up Area in a Spatially Explicit Way: HYDE 3.1. *The Holocene*, 20(4):565–573, June 2010. ISSN 0959-6836, 1477-0911. doi: 10.1177/0959683609356587.

Anthony Kiszewski, Andrew Mellinger, Andrew Spielman, Pia Malaney, Sonia Ehrlich, and Jeffrey Sachs. A Global Index Representing the Stability of Malaria Transmission. *American Journal of Tropical Medicine and Hygiene*, 70(5):486–98, 2004.

Navin Ramankutty, Jonathan Foley, John Norman, and Kevin McSweeney. The Global Distribution of Cultivable Lands: Current Patterns and Sensitivity to Possible Climate Change. *Global Ecology and Biogeography*, 11(5):377–392, 2002.

U.S. Geological Survey. GTOPO30. *Sioux Falls, SD: U.S. Geological Survey Center for Earth Resources Observation and Science*, 1996.

Päivi Lujala, Jan Ketil Rød, and Nadia Thieme. Fighting over Oil: Introducing A New Dataset. *Conflict Management and Peace Science*, 24(3):239–256., 2007.

Päivi Lujala, Nils Petter Gleditsch, and Elisabeth Gilmore. A Diamond Curse? Civil War and a Lootable Resource. *Journal of Conflict Resolution*, 49(4):538–562, 2005.
