# Supplementary material for: Religion and educational mobility in Africa
Source: Nature. 2023 May 17;618(7963):134–43. doi: 10.1038/s41586-023-06051-2 (PMC10232358; doi:10.1038/s41586-023-06051-2)
Supplement: Supplementary file 2 — Reporting Summary [file 41586_2023_6051_MOESM2_ESM.pdf]

nature portfolio

Corresponding author(s): *Stelios Michalopoulos (Brown University)*

Last updated by author(s): *2023- 01- 24*

Reporting Summary

Nature Portfolio wishes to improve the reproducibility of the work that we publish. This form provides structure for consistency and transparency in reporting. For further information on Nature Portfolio policies, see our Editorial Policies and the Editorial Policy Checklist.

Statistics

For all statistical analyses, confirm that the following items are present in the figure legend, table legend, main text, or Methods section.

n/a	Confirmed	
☐	☒	The exact sample size (*n*) for each experimental group/condition, given as a discrete number and unit of measurement
☒	☐	A statement on whether measurements were taken from distinct samples or whether the same sample was measured repeatedly
☐	☒	The statistical test(s) used AND whether they are one- or two-sided<br>*Only common tests should be described solely by name; describe more complex techniques in the Methods section.*
☐	☒	A description of all covariates tested
☐	☒	A description of any assumptions or corrections, such as tests of normality and adjustment for multiple comparisons
☐	☒	A full description of the statistical parameters including central tendency (e.g. means) or other basic estimates (e.g. regression coefficient) AND variation (e.g. standard deviation) or associated estimates of uncertainty (e.g. confidence intervals)
☐	☒	For null hypothesis testing, the test statistic (e.g. *F*, *t*, *r*) with confidence intervals, effect sizes, degrees of freedom and *P* value noted<br>*Give P values as exact values whenever suitable.*
☒	☐	For Bayesian analysis, information on the choice of priors and Markov chain Monte Carlo settings
☒	☐	For hierarchical and complex designs, identification of the appropriate level for tests and full reporting of outcomes
☒	☐	Estimates of effect sizes (e.g. Cohen's *d*, Pearson's *r*), indicating how they were calculated

Our web collection on statistics for biologists contains articles on many of the points above.

Software and code

Policy information about availability of computer code

Data collection	We use individual-level data from IPUMS International, which collects, cleans, and harmonizes Census data from various African countries. Researchers were not involved in data collection.
Data analysis	The code used to construct and analyze the data was written in R v.4.2.2 and Python v.3.11.

For manuscripts utilizing custom algorithms or software that are central to the research but not yet described in published literature, software must be made available to editors and reviewers. We strongly encourage code deposition in a community repository (e.g. GitHub). See the Nature Portfolio guidelines for submitting code & software for further information.

Data

Policy information about availability of data

All manuscripts must include a data availability statement. This statement should provide the following information, where applicable:

- Accession codes, unique identifiers, or web links for publicly available datasets
- A description of any restrictions on data availability
- For clinical datasets or third party data, please ensure that the statement adheres to our policy

The replication code and data files are available on https://github.com/imreligionafrica/imreligionafrica The individual level data from IPUMS International are available here: https://international.ipums.org/international/. here

Human research participants

Policy information about studies involving human research participants and Sex and Gender in Research.

Reporting on sex and gender

The main analysis does not distinguish by sex. In the Supplementary Information, we report some results separately for girls and boys. The underlying information on sex comes from IPUMS International, which in turn collects and harmonizes Census data collected by national statistical agencies. Primary data collection, recruitment, and consent was done by the national statistical agencies conducting the Censuses.

Population characteristics

Young individuals, aged 14-18 years and 14-25 years, who cohabitate with at least one older generation member in the household, typically a biological parent.

Recruitment

The paper conducts secondary data analysis only. Primary data collection, recruitment, and consent was done by the national statistical agencies and this is described in depth at the respective technical reports, alongside the manuals in IPUMS International.

Ethics Oversight

Because our analyses are based on secondary, de-identified, publicly available data, we do not have an IRB waiver.

Note that full information on the approval of the study protocol must also be provided in the manuscript.

Field-specific reporting

Please select the one below that is the best fit for your research. If you are not sure, read the appropriate sections before making your selection.

☐ Life sciences ☒ Behavioural & social sciences ☐ Ecological, evolutionary & environmental sciences

For a reference copy of the document with all sections, see nature.com/documents/nr-reporting-summary-flat.pdf

Life sciences study design

All studies must disclose on these points even when the disclosure is negative.

Sample size

Data exclusions

Replication

Randomization

Blinding

Behavioural & social sciences study design

All studies must disclose on these points even when the disclosure is negative.

Study description

We compile measures of absolute intergenerational mobility in educational attainment for Africans adhering to different religions across African countries and regions and analyze the features shaping differences across faith in educational mobility.

Research sample

Individual level data of intergenerational mobility in education based on matched children-parents educational attainment data from 21 African countries, available via IPUMS International. Data is disaggregated by religion, country, regions, gender, rural-urban, and schooling level (completed primary or higher versus non-completed primary). For most countries, IPUMS gives a representative 10% sample.

Sampling strategy

We use all observations from IPUMS International that meet the sample criteria; namely, 14-18 year old and 14-25 year old individuals who cohabitate with at least one older generation member in the household with available information on education and religious affiliation. IPUMS International uses mainly 10% sample from 21 African countries. See Supplementary Information for details, please.

Data collection	Researchers were not involved in data collection. All data are retrieved from IPUMS - International, which in turn collects and harmonizes Census data and provides to the public representative, typically 10%, samples. To access the data, the prospective user may submit an electronic authorization form providing name, electronic address, and institutional affiliation here: https://international.ipums.org/international/.
Timing	We use all Censuses from African countries with information on religious affiliation since independence. Most Censuses were conducted in the 1990s and 2000s. The earliest Census is in 1970 and the latest in 2016. See please the Supplementary Information for details.
Data exclusions	We did not exclude any individuals who met the Research Sample criteria.
Non-participation	N/A
Randomization	The data is observational and there is no random variation. We discuss how our correlational results should be interpreted.

Ecological, evolutionary & environmental sciences study design

All studies must disclose on these points even when the disclosure is negative.

Study description	
Research sample	
Sampling strategy	
Data collection	
Timing and spatial scale	
Data exclusions	
Reproducibility	
Randomization	
Blinding	

Did the study involve field work? ☐ Yes ☑ No

Field work, collection and transport

Field conditions	
Location	
Access & import/export	
Disturbance	

Reporting for specific materials, systems and methods

We require information from authors about some types of materials, experimental systems and methods used in many studies. Here, indicate whether each material, system or method listed is relevant to your study. If you are not sure if a list item applies to your research, read the appropriate section before selecting a response.

Materials & experimental systems

n/a	Involved in the study	
☑	☐	Antibodies
☑	☐	Eukaryotic cell lines
☑	☐	Palaeontology and archaeology
☑	☐	Animals and other organisms
☑	☐	Clinical data
☑	☐	Dual use research of concern

Methods

n/a	Involved in the study	
☑	☐	ChIP-seq
☑	☐	Flow cytometry
☑	☐	MRI-based neuroimaging

Antibodies

Antibodies used

Validation

Eukaryotic cell lines

Policy information about cell lines and Sex and Gender in Research

Cell line source(s)

Authentication

Mycoplasma contamination

Commonly misidentified lines
(See ICLAC register)

Palaeontology and Archaeology

Specimen provenance

Specimen deposition

Dating methods

☐ Tick this box to confirm that the raw and calibrated dates are available in the paper or in Supplementary Information.

Ethics oversight

Note that full information on the approval of the study protocol must also be provided in the manuscript.

Animals and other research organisms

Policy information about studies involving animals; ARRIVE guidelines recommended for reporting animal research, and Sex and Gender in Research

Laboratory animals *For laboratory animals, report species, strain and age OR state that the study did not involve laboratory animals.*
